# Changes in patient conceptualization of asthma and asthma self‐management at key timepoints across the COVID‐19 pandemic: A qualitative study

**DOI:** 10.1111/bjhp.70108

**Published:** 2026-08-25

**Authors:** Aphra Kite, Daniela Ghio, Chris Keyworth, Tracy Epton

**Affiliations:** ^1^ Manchester Centre for Health Psychology University of Manchester Manchester UK; ^2^ School of Psychology University of Leeds Leeds UK

**Keywords:** asthma, COVID‐19, pandemic, qualitative, Transdiagnostic model

## Abstract

**Background:**

The COVID‐19 public health emergency had significant impacts on people living with long‐term health conditions. The specific impacts of COVID‐19 on people with asthma across key stages of the pandemic are less certain. This study therefore aimed to explore the experiences of people with asthma across the pandemic as risk levels changed in a retrospective cross‐sectional study.

**Methods:**

Semi‐structured interviews (*N* = 20), conducted at one timepoint, asked about experiences covering pre‐COVID, initial outbreak/lockdown, restrictions lifting, key‐stages salient to them (e.g., vaccination) and longer‐term. Data were analysed using thematic‐analysis.

**Findings:**

There were three themes. ‘*Doing my own risk assessment*’ was an active process using pre‐existing/acquired knowledge to determine risk and subsequent asthma‐management/COVID‐19 behaviours at all stages. ‘*Asthma from background to foreground*’ highlighted how asthma that was mostly previously unobtrusive was suddenly a ‘pre‐existing condition’ leading to prolonged stress and anxiety. ‘*Place in society*’ describes how the participants were included/excluded by society due to safety concerns from the initial outbreak onwards, which was influenced by government, friends, family and neighbours, the media, health care systems and even themselves.

**Discussion:**

Findings highlight how some people with asthma experienced the pandemic as a threat to mortality, with evidence of both poor and good adjustment to their asthma management, as per the Transdiagnostic model. When there are future rapid changes in contexts affecting long term conditions, government agencies should use the present study's findings to (a) guide decision‐making, (b) inform public health messaging aimed at targeting at‐risk populations and (c) provide support for at‐risk populations.

**Statement of Contribution**

**What is already known?**

- Poor adjustment to Covid‐19 in people with asthma included: increased loneliness and fear; decreased mental health.
- Good adjustment included: increased adherence to medication; becoming more health conscious
- Most previous studies are limited by exploring one stage of the pandemic rather than changes in adjustment over time.

**What does this study add?**

- Applies the Transdiagnostic Model of Adjustment to change in asthma management and perceptions from the start of the pandemic to post‐vaccination (2020–2022)
- Identification of critical events: identity changes; vaccine availability; changing status of asthma
- Perceptual shift from being protected to marginalized from lifted restrictions and public attitude changes

Asthma is a chronic inflammatory pulmonary disease (Strine et al., 2004) that induces symptoms such as a tight chest, coughing, wheezing and breathlessness (Asthma UK, 2024). It affects around 262 million people worldwide (WHO, 2024), 4.3 million in the UK (Mukherjee et al., 2016). Poor asthma symptom control negatively affects perceived quality of life in multiple domains, such as social and physical functioning and bodily pain (Correia de Sousa et al., 2013; Goldney et al., 2003). Exploring the experiences of patients with asthma is necessary during respiratory disease pandemics, as anxiety and stress exacerbate asthma symptoms (Murray & O'Neill, 2018). This research could inform tailored clinical support services (Juniper, 2005); public health messaging to at‐risk groups and inform governments on how their leadership and actions affected people with asthma during the pandemic.

COVID‐19, an infectious respiratory disease, was declared a pandemic on 11 March 2020 (WHO, 2020a), at which point the World Health Organization (WHO) labelled people with moderate and severe asthma as clinically vulnerable to severe symptoms if infected (Assaf et al., 2023; WHO, 2020b). In the early stages of the pandemic the belief that people with asthma were at increased risk of contracting COVID‐19 and experiencing more severe symptoms was based on evidence from other respiratory diseases; this belief about increased risk was revised after further research (Assaf et al., 2023). The Transdiagnostic Model of Adjustment (TMA; Moss‐Morris, 2013) states threats to mortality, such as that experienced by people with asthma in the early stages of the COVID‐19 pandemic, are *key critical events*. The model proposes that *key critical events* and *possible ongoing illness stressors* disrupt *emotional equilibrium and current quality of life*. These are moderated by *personal background factors* (e.g., values and life goals), *illness specific factors* (e.g., nature of symptoms) and *background social and environmental factors* (e.g., availability of health care). Successful adjustment is related to cognitive factors (e.g., good self‐efficacy, sense of control) and behavioural factors (e.g., coping through problem focused strategies); these lead to less distress and impact of illness on life; good illness management and high positive affect. Poor adjustment is related to other cognitive (e.g., high perceived stress, helplessness) and behavioural factors (e.g., coping through avoidance); these lead to disproportionate distress and impact on life; poor illness management and low positive affect.

There is evidence that experiences of the COVID‐19 pandemic resulted in poor adjustment to asthma. Surveys and interviews found that wellbeing (i.e., ‘a positive state experienced by individuals…’; WHO, 2021, p.10) was negatively influenced. People with asthma reported decreases in mental health such as increased anxiety (Higbee et al., 2021; Philip, Cumella, et al., 2020; Santillo et al., 2023), stress (Pedrozo‐Pupo & Campo‐Arias, 2020) and depression (Higbee et al., 2021; Pedrozo‐Pupo & Campo‐Arias, 2020). They reported increased loneliness (Philip, Cumella, et al., 2020) and insomnia (Pedrozo‐Pupo & Campo‐Arias, 2020) that can lead to declines in mental health. Throughout the pandemic there were also reports of increased risk perceptions (i.e., an individual's perceived susceptibility to a threat that includes cognitions and /or affective components) such worry (Higbee et al., 2021; Santillo et al., 2023), fear (Philip, Lonergan, et al., 2020), perceived vulnerability (Santillo et al., 2023); perceived severity due to asthma complications (Santillo et al., 2023); and concerns about health (Philip, Cumella, et al., 2020). There were also background social and environmental factors that influenced the negative impact of COVID‐19 on adjustment such as disruptions to health care (e.g., appointment cancellations; lack of asthma reviews; change to appointment delivery mode) (Philip, Cumella, et al., 2020; Santillo et al., 2023). Other health protective behaviours may have been negatively influenced due to the pandemic. For example, there were reports that some people with asthma were reducing their physical activity (Rogers et al., 2020); possibly for fear of it worsening their symptoms, requiring health care services and increasing the chances of COVID‐19 exposure (Tyson et al., 2022). This is concerning as evidence suggests that physical activity acts as a protective factor against asthma development (Eijkemans et al., 2012) and is beneficial for health in general (Kokkinos, 2012; Rhodes et al., 2017).

In contrast to the factors related poor adjustment, there is also some evidence suggesting that the pandemic may have affected asthma management behaviours suggesting good adjustment. The pandemic prompted some asthma patients to take their diagnosis more seriously to become more health conscious generally (e.g., keeping fit) (Santillo et al., 2023; Tyson et al., 2022); to increase use of their self‐management strategies (e.g., avoiding triggers; brushing up on inhaler techniques; monitoring peak flow; Philip, Cumella, et al., 2020; Santillo et al., 2023) and seek more information about their risk (Santillo et al., 2023). Furthermore, medication adherence improved as more asthma medication was prescribed and taken (Dhruve et al., 2022; Santillo et al., 2023).

The adjustment of asthma patients during the pandemic remains under‐researched with most research focusing on the initial stages (March – May 2020; Dhruve et al., 2022; Higbee et al., 2021; Pedrozo‐Pupo & Campo‐Arias, 2020; Philip, Cumella, et al., 2020; Rogers et al., 2020; Tyson et al., 2022) or the latter stages of the pandemic (2021–2022; Santillo et al., 2023). Moreover, there are few studies that examine change in adjustment in asthma patients over multiple key stages of the pandemic as messaging and risk levels changed. The aim of the present research was to understand asthma patients' retrospective experiences of the pandemic and explore wellbeing and behaviour with cross‐sectional interviews covering key stages of the pandemic (from March 2020 to Autumn 2021).

## METHODS

### Participants

Opportunity sampling was used to recruit 20 participants who met the inclusion criteria of: 18 years old or over; a self‐reported diagnosis of asthma; and living in the UK at the time of the COVID‐19 pandemic. All potential participants who approached us were eligible, which we confirmed via email correspondence, and were included in the study. There were no dropouts. The study design enabled in‐depth exploration of dynamics in health‐related experiences, increasing the information contribution of each participant. Seventeen participants were included in the initial sample, reflecting a considered judgement of adequacy in relation to the study design and aims during the analysis. We included a further three additional participants as they had already been recruited. These additions contributed to an opportunity to further develop our analysis across the different time points. Consistent with reflective thematic analysis, sample size was not determined by saturation (Braun & Clarke, 2021) but was driven by our team's ongoing, reflexive consideration of the richness, relevancy, and explanatory potential of the dataset, especially at these different time points. The study was advertised via Twitter (X) and Facebook. An advertisement was also sent via a daily update email to students and staff working within a university in the Northwest of England. Data were collected between September 2021 and June 2022.

### Materials

#### Demographics

A demographic questionnaire included items on age, gender, ethnicity, and education level.

#### Asthma characteristics

Asthma characteristics were gathered using two questionnaires. The 7‐item Asthma Control Questionnaire (ACQ, Juniper et al., 2006; adapted to 6 items for this study^1^) measures the extent to which current asthma treatment is minimizing symptoms over the previous week (0 = *no impairment in asthma control*; 7 = *severe impairment*). For example, one question asks, ‘During the past week how were your asthma symptoms when you woke up in the morning?’. The 32‐item Asthma Quality of Life Questionnaire (AQLQ, Juniper et al., 1999) measures perceived quality of life across 4 domains: activity limitation, symptoms, emotional function and environmental. Answers are given via a 7‐point scale, with higher scores indicating better quality of life.

#### Topic guide

The topic guide was developed to address all topics pertaining to the research aims. The topic guide was designed to retrospectively proceed through several key stages across COVID‐19, in chronological order (see Table 1 for a COVID‐19 timeline; Brown et al., 2021; Gov.UK, 2020; Gov.UK, 2021; Guardian, 2020; Runswick‐Cole, 2020), which were selected due to the differing levels of restrictions, and therefore the perceived risk, that were in place during the pandemic at each time. The stages were: prior to the pandemic (S0); beginning of the outbreak/lockdown (March 2020; S1); lockdown lifting with some restrictions in place (July 2020; S2); and all restrictions lifted/‘freedom day’ (July 2021; S3). A final section asked questions pertaining to the vaccine (December 2020 onwards) and longer‐term experiences (S4) as well as experiences of contracting COVID‐19 (if applicable). The topic guide was piloted before the study and amended according to feedback (See supplementary materials for full Topic Guide). It covered information received; changes in behaviour (including asthma management, health behaviours); and thoughts, feelings and experiences around asthma (including wellbeing and physical health).

**TABLE 1:** COVID‐19 timeline.

Study time	Date	General population	People with asthma
S0	Pre March 2020	No pandemic	
S1	11 March 2020	COVID‐19 pandemic declared	
	23 March 2020	Full lockdown in United Kingdom	
	End March 2020		Asked to shield, if severe asthma, and aid provided (e.g., food & medication deliveries)
	May 2020		Some removed from shielding list
S2	July 2020	Lockdown lifted	
	August 2020		Shielding paused
	December 2020	Vaccinations available for prioritized	Priority for some with severe asthma
S3	July 2021	Restrictions lifted ‘freedom day’	
S4	Autumn 2021	Vaccinations available for lower risk	

### Procedure

Interested participants were emailed the participant information sheet and a suitable date and time was arranged for the online interview (conducted on Zoom, audio recorded and autotranscribed with no field notes taken). The participants did not meet the interviewer (AK; Female) before the interview; the participant information sheet advised them that she was an MSc student who was an inexperienced interviewer conducting the research for her dissertation under the supervision of experienced qualitative researchers. The participants were not made explicitly aware that the interviewer had asthma although this was often implicit from the interaction during the interview. All participants were interviewed without anyone other than the interviewer and interviewee present. At the interview consent was obtained and the questionnaires were completed verbally. Prior to the interview, participants were given a definition of asthma management for the purposes of clarity. The participants were then interviewed by AW using the topic guide. Participants were thanked for their time. Each participant took part in a single interview and did not comment further on the transcripts/findings.

### Reflexivity statement

The first author is a female with asthma and experienced living with asthma in the UK for the duration of the pandemic (AK). This may have impacted the data as participants may have recognized a shared understanding of pandemic experiences. The data analysis was conducted with the research team (AW; TE; CK; DG) who have specific expertise in behaviour change during pandemics which led to an interest in what happens when information is evolving and changing. A member of the research team (DG) has expertise in the management of long‐term conditions, and this may have focused the analysis on changes to conceptualization and self‐concept. These experiences, expertise and interests may have influenced the analysis and shaped the findings.

### Analysis

Because of the uniqueness of the data set covering different stages of the pandemic we used a hybrid framework (Gale et al., 2013) and thematic analysis (Braun & Clarke, 2019) to explore the themes over multiple stages of the pandemic. We first performed a thematic analysis on the data and then mapped the data supporting each theme onto the stages of the pandemic (see supplementary materials). An inductive, realist approach was taken during analysis, whereby the accounts given were assumed to be an accurate reflection of the participants' experiences. Braun and Clarke's (2019) guidance for thematic analysis was followed to produce the results: Data familiarization was achieved by reading and re‐reading each transcript. Initial codes were compiled, by one coder (AK), by reading each transcript and labelling information relevant to the research question with codes line by line using NVivo software. These codes were discussed with the team which led to themes compiled by grouping together associated concepts. The themes were then reviewed by checking against codes, extracts and transcripts to validate the pattern; then defined and named (all the research team). This process ensured confirmability and transferability of findings. Prior to the final task of writing up the themes, they were mapped (i.e., Framework analysis), by one researcher (TE) to allow for a different member to familiarize themselves with the organized data, against each stage of the pandemic explored (S0 prior to the pandemic; S1 start of the outbreak/lockdown; S2 lifting of lockdown with some restrictions; S3 complete lifting of restrictions/ freedom day; S4 when vaccines had been developed). Each quote presented includes the stage of pandemic for reference.

### Ethical approval

Ethical approval for the study was granted by a University Research Ethics Committee (reference: 2021‐11163‐20625).

## RESULTS

Twenty participants were recruited. Most participants were female (80%). Mean age was 38.75 years (range: 23–54; *SD *= 9.81). The mean score for the ACQ was 1.17 (range: .00–2.42; *SD* = .58), showing good control and for the AQLQ it was 5.74 (range: 4.00–6.69; *SD* = .83), indicating a fairly high quality of life at the time of interview. (See Table S1).

Three key themes were developed from the data: ‘*Doing own risk assessment*’; ‘*Asthma: background to foreground*’ and ‘*Place in society: left out and left behind*’. Table 2 shows the themes within each stage of the pandemic (Tables S2–S4 in the supplementary materials show all the quotes for each theme).

**TABLE 2:**
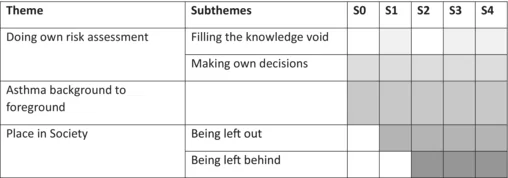
Thematic map of stages of the pandemic and themes.

*Note*: S0 = pre‐COVID; S1 = beginning of the outbreak/lockdown (March 2020); S2 = lockdown lifting with some restrictions in place (July 2020); S3 = all restrictions lifted/‘freedom day’ (July 2021); S4 = vaccine (December 2020 onwards)/longer‐term experiences/contracting COVID‐19. Shading = theme mentioned at that stage of the pandemic.

### Theme 1: Doing own risk assessment

This theme explores an active process using pre‐existing/acquired knowledge to determine risk and subsequent asthma‐management and COVID‐19 behaviours used at all stages of the pandemic. There are two subthemes: ‘*Filling the knowledge void*’ and ‘*Making own decisions*’.

#### Filling the knowledge void

During the initial outbreak/lockdown participants reported a dearth of advice and information from governments and health organizations about COVID‐19, generally and about COVID and asthma specifically. As the pandemic progressed, there was conflicting or changing information and advice as scientific knowledge developed and was disseminated.

And he [GP] said as long as you keep up with your asthma medication, and you're sensible you should be okay. But then, fairly soon after the official lockdown, I had a phone appointment with the asthma nurse. And she said, be exceptionally careful, because this is a respiratory virus, you've got a respiratory disease, even if you're not officially told to shield I would do so. (Julia; S1)

At the initial outbreak/lockdown participants perceived a lack of help from government sources or from asthma organizations so some participants were using their own specialist knowledge, from their professional backgrounds (e.g., medical student, biological background; official guidance obtained from their job in HR, historian), to determine what they should do to protect themselves.

I mean, when people sort of doing crazy things like not sitting on benches … it seemed to me like from having a biological background and that kind of behaviour was, was not necessary (Molly; S1)

Other people either actively avoided gaining new knowledge at the beginning of the outbreak by avoiding the news to reduce anxiety or passively avoided it as the pandemic progressed when it wasn't foregrounded in the news.

I guess kind of with all it the not being in the news anymore, you're not seeing the daily death counts like you were before, so you do sort of start to think about it a bit less (Amy; S3)

Other participants, without any perceived relevant specialist knowledge, used scientific or health‐related credible sources (e.g., published academic papers; WHO, Asthma helplines; Asthma UK). Some did not seek further knowledge but used common sense both in the initial phases and when restrictions were lifted. For the participants, they felt that it posed a serious threat for them, even when others viewed it as less threatening as the pandemic progressed.

[Friend was saying] ‘Oh they're hysterical over here you know, it's ridiculous, you know, it's only like a flu’. And I was saying ‘it's not like a flu they wouldn't close the whole fucking economy down for fucking flu’. (Chris; S3)

Most participants also sought out knowledge about the vaccine. This included information about if they were prioritized for getting the vaccine early, which vaccine to have, vaccine effectiveness; how the vaccine worked and vaccine safety. This information was from credible sources; (i.e., Asthma UK; JVCI/ government; WHO; Independent Sage; NHS webpages; academic articles; GPs); news outlets; their own knowledge (e.g., from their lived current experience working from a pharmacology company) and personal contacts.

I read the, the primary, the primary articles, and of AstraZeneca, and the Pfizer and I decided at that point that I wanted Pfizer […] I kept checking the government JVCI guidance, erm because I wanted to know what priority. I was in, and I wanted to know how quickly I was gonna get it. (Matt; S4)

#### Making own decisions

People made their own decisions about asthma management (both prior to and during COVID‐19); COVID‐19 prevention behaviours and vaccine uptake did not necessarily stem from medical or public health advice but by “*doing my own risk assessment*” (Chris, S1).

Pre COVID‐19 participants varied in adherence to asthma management advice with some people strictly adherent to medications and advice (e.g., after a severe event). Some were not fully following advice (e.g., not carrying inhalers), taking alternative medications (e.g., ordered cheaply from India; home remedies) or increasing medications when they felt it was needed (e.g., when fell ill; when near triggers).

I tend to if I'm having a really bad attack, I'll tend to have a teaspoon of turmeric and boiling water (Daisy, S0)

The initial outbreak/lockdown affected asthma management in various ways. Some participants made no changes to their asthma management as they thought it was unnecessary as it was managed well (e.g., as some triggers were avoided). Others became more adherent due to concerns about contracting COVID‐19 (e.g., regular peak‐flows, taking preventers) although one stopped once they felt safer (e.g., reduced preventer use after initial increase). Others did extra because of their fear of COVID‐19 (e.g., bought pulse oximeter, gave up smoking, stocked up on inhalers/medications/home remedies). However, the lockdown also had negative effects on asthma management, despite understanding the risks, as they failed to follow medical advice due to breaking habits during lockdown or because the risk of contracting COVID‐19 outweighed the risks of asthma.

… eventually it was lunchtime and I realized, oh no I haven't even brushed my teeth yet and therefore I haven't taken my inhaler (Francesca, S1)

During the period of some restrictions lifting, people still weighed up their risks. Some carried on as they had done during lockdown as their asthma was well controlled; others made more effort as they felt they needed more protection after being de‐prioritized from getting the vaccine or because they wanted to avoid hospitalization. Others found their management continued to be less successful than pre COVID‐19 despite their best efforts.

I knew that actually my asthma's not controlled, I need to get it sorted because I need to protect myself if I'm not gonna be prioritised for a booster. I mean, I can't, I can't be living like suboptimal (Hadley, S2)

People also made their own decisions about which COVID‐19 prevention behaviours they did based on their own risk assessment which occurred throughout the pandemic. People who felt vulnerable due to their asthma reported a suite of protective behaviours. This included (a) completely avoiding contact with potentially infectious people (e.g., early lockdown and shielding even if not advised); (b) behaviours that facilitated shielding (e.g., practical social support; food deliveries; prescription deliveries); (c) behaviours that avoided the likelihood of coming into contact with people (e.g., going places at less busy times; driving rather than public transport; shielding after advice was lifted); and (d) behaviours that stopped the spread of particles (e.g., physical distancing; mask wearing; wiping down objects; using hand sanitizer; washing hands; limiting number of people in groups).

I just kind of missed out on shielding but to my mind I kind of counted myself within that, because I thought well, I it's only just by accident that I'm not in that group, erm so I did take it quite seriously at the start … And you know, we did really try our best to, my husband was doing the shopping rather than me going out, erm, that kind of thing, so yeah (Amy, S1)

It was terrifying. And er I'm sure you've heard this a lot. The so‐called Freedom Day was lockdown day again for me (Julia, S3)

In addition to the standard COVID‐19 protection behaviours some participants also weighed up the risks of allowing service people into the house; doing other health behaviours such as physical activity or seeking emergency or standard medical care.

I don't think so. but I didn't go out cycling, because I was concerned about if I had an accident, that actually that would be a burden on the NHS or I could put myself at risk. (Hadley, S1)

So I'm like again in my head, Dooms Day thinking, I'm thinking yeah if there's 30 people in that GP surgery one might have it, well sod's law, I might get it. And I haven't got a vaccine, so it's gonna, it's going to topple me over quite so yeah I was very much like, to be honest you know you said about the asthma review, they wanted me to come for face to face and I was like nah (David, S2)

For most people whether they had been vaccinated and/or were up to date on their boosters affected their decisions around work, socializing and COVID‐19 protective behaviours. A few people did not reduce their COVID‐19 protective behaviours, were still cautious or were performing some protective behaviours as they were still concerned about their asthma because of the overburdened health service.

So, I'd already made the decision that I wasn't coming out until my vaccine, so I was completely staying in the house (Matt, S2)

I think as soon as I got the first one I really did become quite slack, at sort of protecting myself. (Molly, S4)

### Theme 2: Asthma – background to foreground ‘it's only mild or moderate until it's not’

This theme highlights how asthma that was mostly previously unobtrusive was suddenly foregrounded leading to prolonged stress and anxiety. Pre‐COVID‐19, many people did not really think about their asthma; it was in the background and managing it was habitual.

I rarely ever think about it and the inhalers just become part of my brushing my teeth routine and it's just it's just something I do and I often forget that this is medicine… (Francesca; S0)

Asthma was only salient when it was uncontrolled or there was a severe incident (e.g., severe asthma attack, hospitalization) but some were aware that asthma could become more severe. Indeed, contracting COVID‐19 also made asthma more salient.

And this is one of the things that annoys me so much when you define it as 'oh it's mild or moderate or severe', well it's only mild or moderate until it's not (Julia, S0)

The pandemic brought asthma to the foreground for many people with the realization that asthma was a ‘*pre‐existing condition*’ (Amy, S2) with an accompanying change in illness perceptions. This was especially apparent during the initial outbreak/lockdown but did continue over the stages of pandemic and in the longer‐term.

It [asthma] was always quite like just an average thing and then I guess it was like being classed as severe asthma and then it was like you need to shield, it was a bit like, it changed it did change my diagnosis in my head. (Louise, S1)

Although some were less concerned as they didn't consider themselves at risk (e.g., they only had mild asthma; it was well controlled); whereas others felt ‘*not completely vulnerable but you're not normal either*’ (Joanna, S1).

I felt okay about it at that point, cos it was still like pretty controlled and I wasn't doing much that would make it worse at the time. (Millie, S1)

For some people getting the vaccine bought asthma to the foreground as they weighed up the risks from asthma and COVID‐19 and vaccine risks; recognizing they had it early due to their asthma or because they needed the vaccine to reduce their vulnerability.

Yeah. just remember hearing like the negative effects of how poorly it [the vaccine] made people and that worried me about getting it and then me getting poorly off it … because I knew I was already at risk with my asthma, and I didn't want to risk for, like, heighten the risk… I knew my risk was still there of getting it [COVID] but it wasn't as much as what it would be if I didn't get the vaccine. (Millie, S4)

Some participants still had their asthma foregrounded even after the vaccine and/or the pandemic risk had subsided.

Even with the injections part of me goes, well, if a bit of hay fever mixes with it, I think I'd end up at the hospital. So, you know, even though I've been jabbed and boosted I think it's in my head that I'd probably end up in hospital (Annie, S4)

### Theme 3: Place in society – Protected and liberated vs. left out and left behind

This theme describes the extent to which participants felt they were included/excluded by society, and their affective reactions, at different stages of the pandemic from the initial outbreak, through restrictions lifting and removal; vaccine rollout and life in the long‐term. Inclusion/exclusion covers the physical environment (i.e., from shielding, locking down), the social environment, health care and society as a whole. Those responsible for the extent of inclusion/exclusion were the participant (e.g., self‐excluding themselves and their family), government, media, health care services, friends, family and neighbours. The level of inclusion in society was related to perceptions of fear and safety; those who were fearful were excluded; and those who felt safe were included. Exclusion by someone else led to anger and frustration.

#### Physical environment

After the initial shock and worry surrounding the outbreak, many people were relieved to be away from people as they were advised by the government to lock down or shield as they wanted to be protected from society and by society. Inclusion occurred at all stages of the pandemic when they felt that other people were ‘*being really respectful about sitting far apart, wearing masks*’ (Joanna, S2) and enacting other COVID‐19 preventative behaviours.

I know I was relieved that I wouldn't have, I wouldn't have to be out there with people mixing and, you know, and that kind of thing (Olivia, S1)

Many participants experienced fear due to safety concerns that often led to further self‐exclusion such as voluntarily shielding, locking down early and isolating themselves from family members to protect themselves from the severe risk. Over time some people were still choosing to self‐exclude and remain physically apart from society. This was especially apparent when the government lifted all restrictions as ‘*Freedom Day was lockdown day again*’ (Julia, S3) as some people were terrified as they were no longer protected by the government and society.

I didn't go near him [husband who was keyworker]. We stayed metres apart and we slept in two separate bedrooms. (Shauna, S1)

#### Social environment

Lockdown and shielding provided time for people to connect online with family, current friends and old friends. People also distracted themselves from the social isolation by finding activities they could do alone. As restrictions were lifted and after freedom day, people some people ‘*definitely took full advantage*’ (Helen, S2). People felt grateful to be able to socialize and ‘*there was a little sense of liberation*’ (Francesca, S2.); although for most this was with a sense of caution (e.g., avoiding crowded places, avoiding risky activities).

I did see more people but in the very careful situations, and more generally, I thought, well, if I can avoid going to anywhere that's crowded or inside or I don't have to that I'm going to (Julia, S2)

Some people experienced exclusion in the initial lockdown as ‘*all of a sudden your life became very very insular*’ (Daisy, S1). As lockdown and restrictions lifted, the people who were concerned about safety (e.g., from contracting COVID‐19 due to risky behaviours of others) were socially excluded as they didn't start up activities they had done previously (e.g., going to the gym, going into shops; socializing with friends) or limited what they did take part in (e.g., only outdoor parent and baby activities). Some people excluded their family members from society (e.g., by keeping their children off school; (restricting family members activities) that led to guilt. Others were left behind by their friends or the social environment had changed (e.g., their local friends had moved away) by the time they felt safe to go out. One person reflected about how the ‘*Rule of 6*’^2^ excluded as ‘*I didn't like it, either way, whether it was me that was being excluded or whether we had to exclude somebody else*’ (Molly, S2).

But my social life really took a dip at that point. Reason being, everybody else was going out and had the rules of six and you know, they weren't in a lockdown anymore. So it was very, I thought, you know we'll forget about him now, so that, yeah, that's kind of what happened around that time (Matt, S2)

#### Health care

Some people experienced inclusion through good health care that accommodated their needs. Most people felt more protected, relieved, and able to enter society again once they had had their vaccine, although most proceeded with caution.

thought, as soon as I'd been injected I was like, thank goodness, I've made it. I've actually made it. Erm because I was just scared about, you know, when you see in the news, they'll let the bodies pile high and things and it's like they're talking about me. (Matt, S4)

Others felt they were excluded from health care because asthma services declined which led to ‘*it's just that feeling of like abandonment really*’ (Molly, long‐term). Some did not feel safe accessing medical treatment at all stages of the pandemic. Others felt that their lives were worth less than others as they were disposable due to their pre‐existing condition. The de‐prioritization of people with asthma for the vaccine caused the most anger and frustration (towards government bodies) and the fight to get the vaccine was regarded as very stressful; this was compounded with fear when they had the first vaccination early but there was a long wait for the booster that left them without protection.

So I'm like again in my head, Dooms Day thinking, I'm thinking yeah if there's 30 people in that GP surgery one might have it, well sod's law, I might get it. (David, S2)

#### Society as a whole

Some participants felt protected, but with caveats, as ‘*we were being looked after as a population. Not necessarily asthmatics but the population as a whole*’ (Hadley, S1).

At times participants felt ‘*abandoned, I guess, and anxious again*’ (Hadley, S4) (e.g., after vaccine de‐prioritization), ‘*invalidated*’ (Matt, S1) (e.g., not immediately being on shielding list) or discriminated against (e.g., stigma from wearing masks) by governments and society. The issues with the Government were ‘*basically that the government that we have, just wasn't trustworthy enough to be able to feel safe*’ (Chris, S3). People also felt that there was a lack of understanding about the plight of people with asthma in general society and even with friends. Conversely, people also started to feel they didn't understand the behaviours of the public and felt negatively about other people because of their attitudes towards COVID‐19 prevention.

with certain friends it was the case of, will you come to the pub? Well I don't want to. Why? because there's a fucking pandemic going on. (Chris, S3)

people judge you they think, Why are you bothering with 2 metre crap? Why are you wearing a mask outdoors if you're meeting a group of people? (Stephen, S2)

The lack of interest and empathy from various sources was also a source of frustration and anger. This came from friends who did not understand the safety concerns of someone with asthma and the media who were not interested in the de‐prioritization around the vaccine.

and she was like going on saying she's so relieved to get the vaccine, and the lack of empathy from my best friend was unbelievable, because she was sorted (Hadley, S4)

We've tried and tried and tried to get the media to take up what's happened. How asthmatic were excluded, so many were excluded. (Olivia, S4)

## DISCUSSION

The aim of the present research was to understand asthma patients' experiences of the pandemic and explore wellbeing and behaviour across key stages of the pandemic. Thematically analysing the data resulted in three key themes: ‘*Doing own risk assessment*’; ‘*Asthma: background to foreground*’ and ‘*Place in society: left out and left behind*’ which fit within the Transdiagnostic Model of Adjustment (Moss‐Morris, 2013) that is used to explain the findings below.

There were several events during the pandemic that seemed to be *key critical events* that *disrupted emotional equilibrium and quality of life* as per the TMA. The announcement of the COVID‐19 pandemic and the initial statement that people with asthma were at high risk was a threat to mortality. There was a corresponding change in identity, as illustrated in Theme 2: *Asthma from background to foreground*, whereby people with asthma were seen as having a pre‐existing condition that was a risk factor for poorer COVID‐19. The availability of vaccines was a positive critical event; however, the downgrading of asthma as a risk factor and people's removal from the vaccine priority risk was a negative critical event.

The above critical events were fed into by *illness specific factors* such as pre‐COVID‐19 asthma related incidents; prior severe medical incidents increased the level of emotional disruption and mild or lack of incidents was related to lower emotional disruption (see Theme 2: *Asthma from background to foreground*). The *background social and environmental factor* that influences the impact of the key critical event was the restricted availability of routine asthma care (Theme 3: *Place in society: left out and left behind*). This was also evident in the literature as some people were not receiving asthma reviews or were only receiving these remotely, which were regarded as inadequate (Santillo et al., 2023). There was evidence of the *disrupted emotional equilibrium and quality of life* in line with prior findings (Higbee et al., 2021; Philip, Cumella, et al., 2020; Santillo et al., 2023); the current study identified worry, fear and panic, particularly at the start of the pandemic.

The findings of this study reflect components of both successful adjustment (leading to good illness management, less distress, high positive affect and less impact on life) and adjustment difficulties (leading to poor illness management, high distress, low positive affect and greater impact on life). Successful adjustment was influenced by cognitive factors such as an increased sense of control by participants *doing own risk assessment* (Theme 1). People were *Making their own decisions* (Theme 1.2) such as using their own specialist knowledge to understand their level of risk. The weighing up of risk was also evident in the extant literature as a response to the lack of information whereby people used sources such as Asthma UK (Santillo et al., 2023). There were variations in perceived social support in those who felt they were *protected & liberated* but not those who felt they were *left out and left behind* (Theme 3). This was in relation to regulations (e.g., feeling protected by lockdowns, mask mandates in early stages; feeling excluded when these were lifted); the extent to which others were performing behaviours (e.g., feeling protected when people performed protective behaviours; feeling excluded when people didn't perform those behaviours) and availability of health care (e.g., feeling protected when health care was available; feeling excluded when health care was not available). This lack of inclusion was also reflected in the literature as people felt the restrictions had been lifted prematurely (Santillo et al., 2023).

Behavioural factors related to successful adjustment were the adherences to asthma medication and self‐management through *making own decisions* (Theme 1.2) as people were taking more care about asthma management as a protective factor against COVID‐19. Management strategies such as stocking up on medication, avoiding triggers, adjusting medications as needed, and monitoring peak flow were also mentioned in the literature (Dhruve et al., 2022; Philip, Lonergan, et al., 2020; Santillo et al., 2023). Performing COVID‐19 behaviours based on *doing own risk assessment* (Theme 1) that was regarding the participants' perceived risk due to their asthma. Those participants who felt most at risk performed COVID‐19 behaviours that were above and beyond those recommended (e.g., locking down earlier and for longer; wearing masks after the mandate had lifted). This was also reflected in other studies whereby high risk perception was related to performance of COVID‐19 prevention behaviours and low risk perception was related to a reduction in that performance (Santillo et al., 2023). Problem focused strategies were used to facilitate good asthma management (e.g., Theme 1.1: *filling the knowledge void*) mentions participants seeking information from credible sources about vaccines and COVID‐19 (also see Santillo et al., 2023). People also sought out social support to facilitate their lockdown and shielding (e.g., shopping deliveries).

Adjustment difficulties were illustrated by both cognitive and behavioural factors. Cognitive factors included high perceived stress whereby participants felt abandoned by their governments and health care providers; there was fear about contracting COVID‐19 and anger, frustration and helplessness when lockdowns ended leaving them *feeling left out and left behind* (Theme 3). This level of concern was highlighted in other studies where people who had not had an asthma review became increasingly worried about their health care and illness (Santillo et al., 2023) and perceptions of inadequate national responses (Philip, Lonergan, et al., 2020).

Behavioural factors related to poor adjustment were mainly avoidance‐related coping. As people were fearful for their health because of their asthma, they avoided the news to reduce stress and they avoided health care and recreational activities to reduce the risk of contracting COVID‐19. Other behavioural factors that inadvertently occurred were the breaking of asthma medication habits as their routine changed.

Based on these findings, there are practical implications to consider around supporting and communicating with those with long term conditions during pandemics. This study highlighted poor communication around the lack of information, conflicting information and changing information that caused elevated distress among participants. Although this is always likely to be the case for novel health risks and with rapidly changing scientific knowledge about those novel health risks there are ways to reduce the level of the distress through better communication strategies actions (Epton & the BPS COVID‐19 behavioural science and disease prevention taskforce, 2020; Ghio et al., 2021). First, framing messages to use clear and appropriate language to emphasize the efficacy of recommended actions (Epton & the BPS COVID‐19 behavioural science and disease prevention taskforce, 2020; Ghio et al., 2021) could have helped to reduce the feelings of panic experienced early in the pandemic. Second, ensuring unified messaging including identifying and explaining inconsistencies from other sources (Epton & the BPS COVID‐19 behavioural science and disease prevention taskforce, 2020; Ghio et al., 2021) to reduce the uncertainty people felt when receiving conflicting advice (in one instance even from the same GP practice). Third, addressing uncertainty and changing information quickly and transparently (Epton & the BPS COVID‐19 behavioural science and disease prevention taskforce, 2020; Ghio et al., 2021) would likely reduce the anger and frustration with perceived delays in information and likely overestimation of risk from doing their own research (Jackson et al., 2021). Also, communicating about changing risks and priorities around shielding and vaccination, and explaining the reasons behind those decisions, may have reduced the distress felt. For example, those patients with well‐controlled symptoms were not as high risk as initially thought (Adir et al., 2021; Mendes et al., 2021; Sunjaya et al., 2022), though it is unclear as to whether this became widespread knowledge.

The adjustment difficulties related to the disruption to in person health care during the pandemic highlighted the need for digital tools and interventions (e.g., electronic monitoring devices, messaging based interventions, mobile apps) for asthma management (Caponnetto et al., 2024; Chan et al., 2022). In addition, findings around people striving to improve their asthma self‐management provide health professionals with a unique opportunity to converse with patients about further improvements to their asthma management and lifestyle behaviours more generally (Tyson et al., 2022); the pandemic has possibly created a teachable moment as it seems that patients may be more motivated to manage their condition compared to pre‐pandemic. This was also highlighted in other studies where asthma reviews with their primary care providers were sought, as a chance to discuss management during the pandemic (Santillo et al., 2023).

Where patients are likely to experience emotional disruption and a reduction in quality of life from pandemics leading to poor adjustment, appropriate psychological support should be signposted and provided to maintain emotional equilibrium. For example, signposting for psychological support could accompany shielding letters (Fisher et al., 2021). Ideally, an integrated care model of physical and mental health support should be provided so referral to mental health pathways would be timely (Isaacs & Mitchell, 2024).

The main strength of the present study is that framing the topic guide longitudinally, with specific stages of the pandemic as contexts changed, was a novel approach to the literature and thus adds value to existing literature. The study had a gender breakdown that reflected the prevalence of asthma in the adult population (NICE, 2025). The study also covered a mid‐point of the pandemic where there is a dearth of research. The present study has limitations that should be noted. First, participants were asked to recall their experiences retrospectively. Although this was unavoidable given that the stages of the pandemic had already passed at the time of the study, asking participants to rely on memory for their responses may have led to slight biases in reporting or accidental omission of interesting information (Snelgrove & Havitz, 2010). However, retrospective recall tends to be more accurate when the topic of recollection relates to salient events (Ericsson & Simon, 1980). Given that COVID‐19 was an unprecedented and therefore highly salient event, it seems that recollection is likely to be accurate. In future pandemics, research should be conducted longitudinally to explore different stages of the pandemic. Second, all participants in the study were white, and the majority white British. It is possible that people with asthma from different ethnic backgrounds had different experiences with the topics covered in the present study and had interesting insights to provide (Burgess et al., 2022). Moreover, only two participants in the present study had been labelled as clinically extremely vulnerable and were advised to shield, meaning the majority had mild or moderate asthma. Although results were still valuable and insightful, the lack of diversity in asthma severity may have limited the breadth of experiences. Although it is noteworthy that even those with mild and moderate asthma underwent a reconceptualization of their health condition and acted to improve their self‐management. Future research should explore the experiences of a more diverse sample. Third, the Transdiagnostic Model of Adjustment was used in the interpretation of results but not used to shape the topic guide so specific questions about each factor were not included. Although this approach ensured that responses were not restricted by the model, this could have resulted in some factors being missed.

This study provides novel insights into how people with asthma experienced and adapted to the changing risks, uncertainties and social contexts associated with the COVID‐19 pandemic. Viewing participants' experiences through the lens of the Transdiagnostic Model of Adjustment proved particularly useful in identifying and demonstrating how key pandemic‐related events – including the initial classification of asthma as a risk factor for severe COVID‐19, changing public health guidance, vaccine rollout and the removal of restrictions – acted as critical events that disrupted adjustment and prompted ongoing reassessment of risk. The findings illustrate the interaction of personal factors (e.g., prior experiences and perceptions of vulnerability), illness‐specific factors (e.g., asthma severity and symptom history) and social and environmental factors (e.g., health care access, public health messaging and societal attitudes) which shaped both adaptive and maladaptive responses. For example, participants frequently engaged in proactive risk assessment, information seeking and enhanced self‐management behaviours that supported adaptation, while others experienced heightened anxiety, avoidance, social exclusion, disrupted health care access and feelings of being left behind as the pandemic evolved. These findings demonstrate that adjustment is not static but is shaped by changing contexts, evolving knowledge and shifting perceptions of risk throughout a public health crisis. Future pandemic preparedness and response planning should therefore recognize the dynamic nature of adjustment among people living with long‐term conditions. Clear, transparent and responsive communication; continuity of health care provision; and timely practical and psychological support are likely to be essential for promoting adaptive adjustment and reducing the unintended negative consequences of future public health emergencies.

## AUTHOR CONTRIBUTIONS

**Aphra Kite:** Conceptualization; investigation; methodology; resources; writing – review and editing; writing – original draft; formal analysis. **Daniela Ghio:** Formal analysis; writing – review and editing. **Chris Keyworth:** Conceptualization; methodology; resources; supervision; writing – review and editing. **Tracy Epton:** Conceptualization; investigation; methodology; writing – review and editing; formal analysis; project administration; supervision; data curation; resources.

## Supporting information


**Table S1.** Participant characteristics.
**Table S2.** Theme 1: doing my own risk assessment.
**Table S3.** Theme 2: Asthma background to foreground “it's only mild and moderate until it's not”.
**Table S4.** Theme 3: Place in society.

## Data Availability

The full dataset is not available due to ethical issues. However, a full set of quotes supporting each theme is included in the supplementary materials.

## Footnotes

^1^
We excluded the last question as the ethics committee suggested that people would not have access to a peak‐flow meter.

^2^
The ‘Rule of 6’ was a UK government regulation restricting the number of people you could meet outside to 6 people in total.

## References

[bjhp70108-bib-0001] Adir, Y. , Saliba, W. , Beurnier, A. , & Humbert, M. (2021). Asthma and COVID‐19: An update. European Respiratory Review, 30(162), 210152. 10.1183/16000617.0152-2021 34911694 PMC8674937

[bjhp70108-bib-0002] Assaf, S. , Stenberg, H. , Jesenak, M. , Tarasevych, S. P. , Hanania, N. A. , & Diamant, Z. (2023). Asthma in the era of COVID‐19. Respiratory Medicine, 218, 10737. 10.1016/j.rmed.2023.107373 37567514

[bjhp70108-bib-0003] Asthma UK . (2024). What is asthma? Asthma + Lung UK. https://www.asthma.org.uk/advice/understanding‐asthma/what‐is‐asthma/

[bjhp70108-bib-0004] Braun, V. , & Clarke, V. (2019). Reflecting on reflexive thematic analysis. Qualitative Research in Sport, Exercise and Health, 11(4), 589–597. 10.1080/2159676X.2019.1628806

[bjhp70108-bib-0005] Braun, V. , & Clarke, V. (2021). To saturate or not to saturate? Questioning data saturation as a useful concept for thematic analysis and sample‐size rationales. Qualitative Research in Sport, Exercise and Health, 13(2), 201–216. 10.1080/2159676X.2019.1704846

[bjhp70108-bib-0006] Brown, J. , Kirk‐Wade, E. , Baker, C. , & Barber, S. (2021). Coronavirus: A history of lockdown laws in England. House of Commons Library. https://commonslibrary.parliament.uk/research‐briefings/cbp‐9068/

[bjhp70108-bib-0007] Burgess, R. A. , Kanu, N. , Matthews, T. , Mukotekwa, O. , Smith‐Gul, A. , Yusuf, I. , Lamptey, I. , McCauley, N. , Wilson, R. , Pirisola, M. , & Gul, M. (2022). Exploring experiences and impact of the COVID‐19 pandemic on young racially minoritised people in the United Kingdom: A qualitative study. PLoS One, 17(5), e0266504. 10.1371/journal.pone.0266504 35507595 PMC9067664

[bjhp70108-bib-0008] Caponnetto, P. , Prezzavento, G. C. , Casu, M. , Polosa, R. , & Quattropani, M. C. (2024). Psychological factors, digital health technologies, and best asthma management as three fundamental components in modern care: A narrative review. Applied Sciences, 14, 3365. 10.3390/app14083365

[bjhp70108-bib-0009] Chan, A. , De Simoni, A. , Wileman, V. , Holliday, L. , Newby, C. J. , Chisari, C. , Ali, S. , Zhu, N. , Padakanti, P. , Pinprachanan, V. , Ting, V. , & Griffiths, C. J. (2022). Digital interventions to improve adherence to maintenance medication in asthma. Cochrane Database of Systematic Reviews, 6, CD013030. 10.1002/14651858.CD013030.pub2 35691614 PMC9188849

[bjhp70108-bib-0010] Correia de Sousa, J. , Pina, A. , Cruz, A. M. , Quelhas, A. , Almada‐Lobo, F. , Cabrita, J. , Oliveira, P. , & Yaphe, J. (2013). Asthma control, quality of life, and the role of patient enablement: A cross‐sectional observational study. Primary Care Respiratory Journal: Journal of the General Practice Airways Group, 22, 181–187. 10.4104/pcrj.2013.00037 PMC6442785 23603870

[bjhp70108-bib-0011] Dhruve, H. , d'Ancona, G. , Holmes, S. , Dhariwal, J. , Nanzer, A. M. , & Jackson, D. J. (2022). Prescribing patterns and treatment adherence in patients with asthma during the COVID‐19 pandemic. The Journal of Allergy and Clinical Immunology: In Practice, 10(1), 100–107. 10.1016/j.jaip.2021.09.032 34610490 PMC8487166

[bjhp70108-bib-0012] Eijkemans, M. , Mommers, M. , Draaisma, J. M. T. , Thijs, C. , & Prins, M. H. (2012). Physical activity and asthma: A systematic review and meta‐analysis. PLoS One, 7, e50775. 10.1371/journal.pone.0050775 23284646 PMC3527462

[bjhp70108-bib-0013] Epton, T. , & the BPS COVID‐19 behavioural science and disease prevention taskforce . (2020). Delivering effective public health campaigns during COVID‐19. British Psychological Society. https://cms.bps.org.uk/sites/default/files/2022‐06/Delivering%20effective%20public%20health%20campaigns%20during%20Covid‐19.pdf

[bjhp70108-bib-0014] Ericsson, K. A. , & Simon, H. A. (1980). Verbal reports as data. Psychological Review, 87, 215–251. 10.1037/0033-295X.87.3.215

[bjhp70108-bib-0015] Fisher, A. , Roberts, A. , McKinlay, A. R. , Fancourt, D. , & Burton, A. (2021). The impact of the COVID‐19 pandemic on mental health and well‐being of people living with a long‐term physical health condition: A qualitative study. BMC Public Health, 21, 1801. 10.1186/s12889-021-11751-3 34620136 PMC8496145

[bjhp70108-bib-0016] Gale, N. K. , Heath, G. , Cameron, E. , Rashid, S. , & Redwood, S. (2013). Using the framework method for the analysis of qualitative data in multi‐disciplinary health research. BMC Medical Research Methodology, 13, 117. 10.1186/1471-2288-13-117 24047204 PMC3848812

[bjhp70108-bib-0017] Ghio, D. , Lawes‐Wickwar, S. , Tang, M. Y. , Epton, T. , Howlett, N. , Jenkinson, E. , Stanescu, S. , Westbrook, J. , Kassianos, A. P. , Watson, D. , Sutherland, L. , Stanulewicz, N. , Guest, E. , Scanlan, D. , Carr, N. , Chater, A. , Hotham, S. , Thorneloe, R. , Armitage, C. J. , … Keyworth, C. (2021). What influences people's responses to public health messages for managing risks and preventing infectious diseases? A rapid systematic review of the evidence and recommendations. BMJ Open, 11, e048750. 10.1136/bmjopen-2021-048750 PMC8587350 34764167

[bjhp70108-bib-0018] Goldney, R. D. , Ruffin, R. , Wilson, D. H. , & Fisher, L. J. (2003). Asthma symptoms associated with depression and lower quality of life: A population survey. Medical Journal of Australia, 178, 437–441. 10.5694/j.1326-5377.2003.tb05285.x 12720509

[bjhp70108-bib-0019] Gov.UK . (2020). Major new measures to protect people at highest risk from coronavirus UK Government Retrieved August 15, 2024, from https://www.gov.uk/government/news/major‐new‐measures‐to‐protect‐people‐at‐highest‐risk‐from‐coronavirus. (Accessed 15 August 2024)

[bjhp70108-bib-0020] Gov.UK . (2021). COVID‐19 vaccination first phase priority groups. Retrieved August 15, 2024, from https://www.gov.uk/government/publications/covid‐19‐vaccination‐care‐home‐and‐healthcare‐settings‐posters/covid‐19‐vaccination‐first‐phase‐priority‐groups

[bjhp70108-bib-0021] Guardian . (2020). Text message tells vulnerable people they are dropped from shielding list Guardian News & Media Limited Retrieved August 15, 2024, from https://www.theguardian.com/world/2020/may/27/phone‐texts‐notify‐cancer‐transplant‐and‐asthma‐patients‐they‐are‐off‐shielding‐list

[bjhp70108-bib-0022] Higbee, D. H. , Nava, G. W. , Kwong, A. S. , Dodd, J. W. , & Granell, R. (2021). The impact of asthma on mental health and wellbeing during COVID‐19 lockdown. European Respiratory Journal, 58(1), 2004497. 10.1183/13993003.04497-2020 33766952 PMC7996219

[bjhp70108-bib-0023] Isaacs, A. N. , & Mitchell, E. K. L. (2024). Mental health integrated care models in primary care and factors that contribute to their effective implementation: A scoping review. International Journal of Mental Health Systems, 18, 5. 10.1186/s13033-024-00625-x 38331913 PMC10854062

[bjhp70108-bib-0024] Jackson, T. , Mcclatchey, K. , Chan, A. , Morgan, N. , & Pinnock, H. (2021). Exploring the psychological impact of the COVID‐19 pandemic on adults with asthma: A qualitative study. European Respiratory Journal, 58, PA3563.

[bjhp70108-bib-0025] Juniper, E. F. (2005). Assessing asthma quality of life: Its role in clinical practice. Breathe, 1(3), 192–204. 10.1183/18106838.0103.192

[bjhp70108-bib-0026] Juniper, E. F. , Bousquet, J. , Abetz, L. , Bateman, E. D. , & Goal Committee . (2006). Identifying ‘well‐controlled’ and ‘not well‐controlled’ asthma using the asthma control questionnaire. Respiratory Medicine, 100(4), 616–621. 10.1016/j.rmed.2005.08.012 16226443

[bjhp70108-bib-0027] Juniper, E. F. , Buist, A. S. , Cox, F. M. , Ferrie, P. J. , & King, D. R. (1999). Validation of a standardized version of the asthma quality of life questionnaire. Chest, 115(5), 1265–1270. 10.1378/chest.115.5.1265 10334138

[bjhp70108-bib-0028] Kokkinos, P. (2012). Physical activity, health benefits, and mortality risk. International Scholarly Research Notices, 2012, 1–14. 10.5402/2012/718789

[bjhp70108-bib-0029] Mendes, N. F. , Jara, C. P. , Mansour, E. , Araújo, E. P. , & Velloso, L. A. (2021). Asthma and COVID‐19: A systematic review. Allergy, Asthma & Clinical Immunology, 17(1), 1–12. 10.1186/s13223-020-00509-y PMC7787409 33407838

[bjhp70108-bib-0030] Moss‐Morris, R. (2013). Adjusting to chronic illness: Time for a unified theory. British Journal of Health Psychology, 18, 681–686. 10.1111/bjhp.12072 24118260

[bjhp70108-bib-0031] Mukherjee, M. , Stoddart, A. , Gupta, R. P. , Nwaru, B. I. , Farr, A. , Heaven, M. , Fitzsimmons, D. , Bandyopadhyay, A. , Aftab, C. , Simpson, C. R. , Lyons, R. A. , Fischbacher, C. , Dibben, C. , Shields, M. D. , Phillips, C. J. , Strachan, D. P. , Davies, G. A. , McKinstry, B. , & Sheikh, A. (2016). The epidemiology, healthcare and societal burden and costs of asthma in the UK and its member nations: Analyses of standalone and linked national databases. BMC Medicine, 14, 113. 10.1186/s12916-016-0657-8 27568881 PMC5002970

[bjhp70108-bib-0032] Murray, B. , & O'Neill, M. (2018). Supporting self‐management of asthma through patient education. British Journal of Nursing, 27, 396–401. 10.12968/bjon.2018.27.7.396 29634337

[bjhp70108-bib-0033] NICE . (2025). Asthma: how common is it National Institute for Health and Care Excellence Retrieved December 8, 2025, from https://cks.nice.org.uk/topics/asthma/background‐information/prevalence/

[bjhp70108-bib-0034] Pedrozo‐Pupo, J. C. , & Campo‐Arias, A. (2020). Depression, perceived stress related to COVID, post‐traumatic stress, and insomnia among asthma and COPD patients during the COVID‐19 pandemic. Chronic Respiratory Disease, 17, 1479973120962800. 10.1177/1479973120962800 33000648 PMC7533953

[bjhp70108-bib-0035] Philip, K. E. , Cumella, A. , Farrington‐Douglas, J. , Laffan, M. , & Hopkinson, N. S. (2020). Respiratory patient experience of measures to reduce risk of COVID‐19: Findings from a descriptive cross‐sectional UK wide survey. BMJ Open, 10, e040951. 10.1136/bmjopen-2020-040951 PMC7482474 32912958

[bjhp70108-bib-0036] Philip, K. E. , Lonergan, B. , Cumella, A. , Farrington‐Douglas, J. , Laffan, M. , & Hopkinson, N. S. (2020). COVID‐19 related concerns of people with long‐term respiratory conditions: A qualitative study. BMC Pulmonary Medicine, 20, 1–10. 10.1186/s12890-020-01363-9 33298023 PMC7724437

[bjhp70108-bib-0038] Rhodes, R. E. , Janssen, I. , Bredin, S. S. , Warburton, D. E. , & Bauman, A. (2017). Physical activity: Health impact, prevalence, correlates and interventions. Psychology & Health, 32(8), 942–975. 10.1080/08870446.2017.1325486 28554222

[bjhp70108-bib-0039] Rogers, N. T. , Waterlow, N. R. , Brindle, H. , Enria, L. , Eggo, R. M. , Lees, S. , & Roberts, C. H. (2020). Behavioral change towards reduced intensity physical activity is disproportionately prevalent among adults with serious health issues or self‐perception of high risk during the UK COVID‐19 lockdown. Frontiers in Public Health, 8, 575091. 10.3389/fpubh.2020.575091 33102424 PMC7554527

[bjhp70108-bib-0040] Runswick‐Cole, K. (2020). A (brief) history of shielding University of Sheffield Retrieved August 15, 2024, from https://www.sheffield.ac.uk/ihuman/news/brief‐history‐shielding

[bjhp70108-bib-0041] Santillo, M. , Tonkin‐Crine, S. , Wang, K. , Butler, C. C. , & Wanat, M. (2023). Management of asthma in primary care in the changing context of the COVID‐19 pandemic: A qualitative longitudinal study with patients. British Journal of General Practice, 30, e903–e914. 10.3399/BJGP.2022.0581 PMC10355814 37429732

[bjhp70108-bib-0042] Snelgrove, R. , & Havitz, M. E. (2010). Looking back in time: The pitfalls and potential of retrospective methods in leisure studies. Leisure Sciences, 32(4), 337–351. 10.1080/01490400.2010.488199

[bjhp70108-bib-0043] Strine, T. W. , Ford, E. S. , Balluz, L. , Chapman, D. P. , & Mokdad, A. H. (2004). Risk behaviors and health‐related quality of life among adults with asthma: The role of mental health status. Chest, 126, 1849–1854. 10.1378/chest.126.6.1849 15596683

[bjhp70108-bib-0044] Sunjaya, A. P. , Allida, S. M. , Di Tanna, G. L. , & Jenkins, C. R. (2022). Asthma and COVID‐19 risk: A systematic review and meta‐analysis. European Respiratory Journal, 59(3), 2004451. 10.1183/13993003.04451-2020 PMC8361304 34385278

[bjhp70108-bib-0045] Tyson, L. , Hardeman, W. , Stratton, G. , Wilson, A. M. , & Semlyen, J. (2022). The effects of social distancing and self‐isolation during the COVID‐19 pandemic on adults diagnosed with asthma: A qualitative study. Journal of Health Psychology, 27, 1408–1420. 10.1177/13591053211012766 33947267 PMC9036148

[bjhp70108-bib-0046] World Health Organization . (2020a). WHO Director‐General's opening remarks at the media briefing on COVID‐19 ‐ 11 March 2020. WHO. https://www.who.int/director‐general/speeches/detail/who‐director‐general‐s‐opening‐remarks‐at‐the‐media‐briefing‐on‐covid‐19‐‐‐11‐march‐2020

[bjhp70108-bib-0047] World Health Organization . (2020b). COVID‐19: Vulnerable and high‐risk groups. WHO. https://www.who.int/westernpacific/emergencies/covid‐19/information/high‐risk‐groups

[bjhp70108-bib-0048] World Health Organization . (2021). Health promotion glossary of terms 2021. WHO. https://iris.who.int/server/api/core/bitstreams/96da8799‐4938‐4d66‐b171‐04770ed4b243/content

[bjhp70108-bib-0049] World Health Organization . (2024). Asthma. WHO. https://www.who.int/news‐room/fact‐sheets/detail/asthma

