# Supplementary material for: Changes in patient conceptualization of asthma and asthma self‐management at key timepoints across the COVID‐19 pandemic: A qualitative study
Source: Br J Health Psychol. 2026 Aug 25;31(3):e70108. doi: 10.1111/bjhp.70108 (PMC13507615; doi:10.1111/bjhp.70108)
Supplement: Supplementary file 1 [file BJHP-31-0-s001.docx]

**Topic Guide**

How did asthma patients feel at different timepoints over COVID-19 as the government changed the level of restrictions?

**Topic Guide (will be amended iteratively)**.

Intro to the research and interview session

“I really appreciate you meeting with me, thank you. I will start recording in a moment but feel free to turn you camera off if that would make you feel comfortable. You can also choose not to answer any questions, or if you need to take a short break at any point let me know and I will pause the recording until you’re ready to carry on” I’ll then take them through the consent questions, the Asthma Control Questionnaire and the Asthma Quality of Life Questionnaire.”

“We’re interested in your asthma management (which is any behaviours you might carry out, such as taking an inhaler, or equally any behaviours you might avoid, like smoking, in an attempt to try and control your asthma symptoms. We’re interested in your asthma management, how you felt and how you acted over different key timepoints. Pre-covid, full lockdown with people shielding or being high risk, lockdown lifting with some restrictions still in place, and other restrictions lifting”

- **Pre-COVID**
  - Can you start by telling me what you typically did to try and manage your asthma before COVID-19, in terms of medication, accessing healthcare services, check-ups etc
  - Prompts
    - How knowledgeable did you feel you were about your asthma and how to control it?
    - How did well managed do you feel your asthma was before COVID-19?
    - How did you feel about your asthma diagnosis before COVID?
- **Lockdown restrictions/people being labelled as high risk/some people being asked to shield**
  - Can you tell me a bit about the advice or information that was given to you by the government at the beginning of pandemic, around the time of the first lockdown? (i.e., around lockdown time) (e.g., shielding; high risk; other recommended behaviours mask wearing / distancing)
  - Prompts:
    - How did you feel about that?
    - What changes did you make in your life (e.g. with managing your asthma)?
    - What effect did that have on your physical or mental wellbeing?
    - How did you feel about your asthma at this point?
- **Lockdown lifting, WFH not mandatory, living but with restrictions**
  - Could you tell me a bit about how life was for you when lockdown was lifted, when we were allowed to leave the house again but with certain restrictions were still in place e.g. masks, social distancing
  - Prompts:
    - How did that make you feel?
    - What changes did you make in your life/to your asthma management?
    - What effect did these changes in restrictions have on you physically?/mentally?
- Other restrictions lifting e.g. masks, no social distancing, no rule of 6 etc
  - Tell me about how your life changed/how your life was for you at this point when restrictions were fully lifted
  - Prompts:
    - How was that for you?
    - How did you feel?
    - What changes did you make regarding your asthma management?
    - How were your experiences with your asthma at this point?
    - What effect did these changes in restrictions have on you?
- Vaccination
  - Can you tell me a bit about the advice and information you remember re the vaccine?
  - Prompts:
    - What changes did you make in your life because you did/ did not have the vaccine (and regarding your asthma management?)
    - What effect did this information and these changes have on your physical and mental wellbeing?
    - How did you feel following your decision to get or not to get the vaccine?
    - Did your asthma factor into your decision making here?
- COVID-19
  - To your knowledge, did you ever catch COVID-19?
  - Prompts
    - How was your experience?
    - How did you manage your asthma at that point?
    - How did you feel towards your asthma whilst you had the virus?

Table S1: Participant Characteristics

| ID number | Age | Gender | Ethnicity | Education | Co-morbidities | AQLQ score | ACQ score |
| --- | --- | --- | --- | --- | --- | --- | --- |
| Amy | 35 | Female | White British | Degree | None | 5.50 | 1.29 |
| Matt | 23 | Male | White British | Degree | Hyperthyroidism | 6.86 | 1.30 |
| Chris | 50 | Male | White British | Degree | Allergies, anxiety, depression | 5.97 | 1.57 |
| Millie | 27 | Female | White British | Degree | Asperger’s | 5.15 | 1.43 |
| Julia | 42 | Female | White British | PhD | Eczema  Nut allergy  PTSD | 5.03 | 1.57 |
| Joanna | 44 | Female | White Other | MSc | None | 5.34 | 0.71 |
| Susan | 54 | Female | White Other | PhD | Depression | 5.66 | 1.14 |
| Molly | 42 | Female | White British | PhD | None | 6.43 | 0.57 |
| Hadley | 46 | Female | White British | PhD | None | 6.03 | 1.71 |
| David | 40 | Male | White British | Degree |  | 4.36 | 1.43 |
| Francesca | 31 | Female | White Other | PhD | None | 6.66 | 0.00 |
| Olivia | 45 | Female | White British | A levels |  | 5.25 | 1.14 |
| Daisy | 55 | Female | White British | Degree | Diabetic | 5.91 | 1.21 |
| Shauna | 45 | Female | White British | PhD | Eczema | 4.00 | 2.42 |
| Robin | 29 | Female | White British | Degree | Eczema | 6.83 | 1.26 |
| Sophie | 44 | Female | White British | Degree |  | 6.47 | 0.00 |
| Stephen | 38 | Male | White Scottish | MSc | Osteopenia | 6.69 | 0.85 |
| Louise | 23 | Female | White/Black Caribbean | MSc | None | 5.34 | 1.43 |
| Annie | 37 | Female | White British | Degree | None | 4.80 | 1.71 |
| Helen | 25 | Female | White British | A levels | None | 6.43 | 0.57 |

Table S2

Theme 1: doing my own risk assessment

|  | Pre-Covid | S1 initial breakdown / lockdown | S2 Some restrictions lifting | S3 All restrictions lifting/ Freedom day | S4 Vaccine and other occasions |
| --- | --- | --- | --- | --- | --- |
| Filling the knowledge void |  | so I called my doctor and he gave me the asthma helpline. Erm that was the only advice I got (Robin)  I was worried. erm I mean, I think because we didn't know so much about the transmission of covid. The thought of getting groceries, and it being on surfaces and things like that. (Olivia)  And he [GP] said as long as you keep up with your asthma medication, and you're sensible you should be okay. But then, fairly soon after the official lockdown, I had a phone appointment with the asthma nurse. And she said, be exceptionally careful, because this is a respiratory virus, you've got a respiratory disease, even if you're not officially told to shield I would do so (Julia)  I remember googling erm asthma.org, like is there any information on there. Erm but I remember feeling very stressed and very panicked because we were kind of like secluded from work, and it, say somebody might have a heart condition and me with asthma like, you're both high risk but when I look back at it now like I wasn't, there just wasn't the information there to, to actually say you'll be okay (Robin)  because I work for the MHR. I tend to to, see a lot of the official advice channels and then follow a lot of GP academics and things with that on medical researchers on social media. And that's for very much a case of well this is up there, this is peer reviewed information. This is scientifically proven. This is real. Erm the, I don't remember any specific advice coming from my GP Practice, because the bar to be classed shielding from asthma was, initially, set, extremely high (Stephen)  It was confusing really confusing. It was not clear at all. You know, I kind of used my own, you know I hate to do this but you know the common sense thing, I thought well look you know this is a, it's a flu it’s a respiratory thing, you know, I'm not going to fare well with asthma (Chris)  We’re both academics and so we, we really, we read up on the science and we follow the guidance and were just very cautious (Francesca)  I had to limit what I watched on the news, because that would set me off panicking and then that would set my asthma off (Shauna) |  | I guess kind of with all it the not being in the news anymore, you're not seeing the daily death counts like you were before, so you do sort of start to think about it a bit less (Amy)  [Friend was saying] “Oh they’re hysterical over here you know, it's ridiculous, you know, it’s only like a flu”. And I was saying it's not like a flu they wouldn’t close the whole fucking economy down for fucking flu. (Chris) | where they monitor people's risks to work out what the rest of groups were and I actually had a go on that I found a link to it. and it said, if I put my details in and said I had asthma, it actually put me at less risk than if I put my details in, and said I didn't have asthma so it was actually saying because i've got asthma i'm at less risk of dying of Covid and I I've can't, I can't see how that could be true. I don't see how if you've already got a respiratory condition, and you catch your respiratory disease, you can be less likely to die of it than say, my husband, who's healthy (Sophie)  Yes, so I read the, the primary, the primary articles, and of AstraZeneca, and the Pfizer and I decided at that point that I wanted Pfizer (Matt)  I certainly read bits and pieces in The Guardian, you know the BBC website. But yeah, it's fairly effective, (Joanna)  well my boyfriend works for Astra Zeneca so that, I knew quite a lot about that. And I was following it really closely in the news and looking at Oxford and trying to figure out all the different kinds of vaccines and how they work and got quite into sort of all the mechanisms that they were targeting and stuff so erm so, yeah, I was fully into reading kind of up on what it all meant and, just waiting to get vaccinated really (Molly)  just remember hearing all like bad stuff about the vaccines, and that worried me about getting it but I was looking online, and I think it's the NHS, and the doctors erm provided like links to the vaccines to look at as well (Millie)  I did do a lot of reading on what the guidance was, because I think, at the start there was there was some confusion about what, who would be included who had asthma and who wouldn't be so, I remember sort of checking asthma UK’s Twitter feed and and reading the guidance that kind of thing to try and work out if I would be in that group or not that was getting it early, and being quite relieved erm that I was (Amy)  I followed World Health Organization on er Twitter. Things like that, Independence sage I was following (Chris)  looking at lancet reports and things like that. I suppose cos I’ve done a science degree and stuff I'm used to reading sciencey sort of stuff (Shauna)  so I kept checking the government jcvi guidance, erm because I wanted to know what priority. I was in, and I wanted to know how quickly I was gona get it. And I remember I think I, I kept umming and ahhing with myself about whether I was, I was in like group three or group seven, and it was, it was just not a nice experience but then I found out that actually you know you'd get offered it just by your GP practice and you'd get a, you get a text and, and that completely got rid of my fears to be honest and I found that out through like BBC News that, that's how I’d get offered it (Matt)  I looked on the NHS web pages, I was reading the newspaper, pretty carefully I was also erm reading explainer articles and things like the conversation (Susan)  well my boyfriend works for Astra Zeneca so that, I knew quite a lot about that. And I was following it really closely in the news and looking at Oxford and trying to figure out all the different kinds of vaccines and how they work and got quite into sort of all the mechanisms that they were targeting and stuff so erm so, yeah, I was fully into reading kind of up on what it all meant and, just waiting to get vaccinated really (Molly)  So I did a bit of research, to be fair, I mean, I’m ex NHS, I worked for the NHS for about 10 years, so my Facebook feed is a lot of NHS friends who were then, to be fair, very pro so they're going like you know vaccines are coming, you know. (David)  Erm, I remember reading about all the different vaccines in the guardian (Daisy)  although I was very glad it was Pfizer that I got it because my friends sister used to work for pfizer and she did say at the start of all this, If I could take any one I would take Pfizer’s (Annie)  I mean, I used to work for a pharmaceutical company so I’m quite comfortable with all the processes (Hadley) |
| Making own decisions | **Asthma Management**  And so ended up in hospital and intensive care, had a cardiac arrest. … Erm so really quite unwell so I was kind of coming off that, recovering from that, I’d become a lot more religious with taking my medication (Matt)  So I always carry my inhaler I take my medications, exactly as prescribed. If I catch a cold, for example I increased my medications to try and keep it under control. (Olivia)  I try and carry my blue inhaler with me i'll be honest sometimes in the summer when the weather is nice and i've not got a cold or anything sometimes the inhaler does get left behind (Sophie)  there's things I avoid but even things that will set me off like bonfires, I know that if I take an extra couples of puffs of blue first, I took my kids to a bonfire thing last year, erm I just took a couple of blue puffs first, and I know I'll be okay if I do that I keep doing inhaler on me, so it's never really stopped me doing anything (Sophie)  I tend to if I'm having a really bad attack, I'll tend to have a teaspoon of turmeric and boiling water (Daisy)  So, I was relying a lot on salbutamol. I wasn't actually getting it from the GP which is really naughty, because my husband works for British Airways and so would go to India quite a lot. And because they were kind of being really strict and monitoring my use and saying you need to have an asthma review, I used to travel a lot for work as well myself it was really inconvenient so he'd actually go and get inhalers of salbutamol when he was in India cos it was like 90p per inhaler as well which is really you know (Hadley) | **Asthma Management**  Yeah, I was definitely more conscious of using my erm preventer inhaler and also using the, the peak flow meter which I've never really used (Amy)  It was terrifying I was in the clinically vulnerable group. But when you looked at like the list of medications I was having 800 of Symbicort, if I'd been on a 1,000 with a montelukast tablets as well, I'd have been shielding so I was only just under the shielding level. So I basically shielded because I just thought look that's so close, you know, and it was so scary (Sophie)  I was obviously taking my reliever inhaler, I’ve got like a oh, what's it called it it’s pulse oximeter, so I bought that during lockdown, just to keep on on my oxygen levels if I did get covid (Amy)  Even when I was recovering [from major medical incident], you know, I'd be like I you know I can social smoke a little bit here and there it's not really a big deal, but since coronavirus hit I was like, No, you can't. You can't smoke a cigarette again, so I think that’s the only other thing I changed in terms of my asthma management (Matt)  Er So, I, I really and I also made sure that I had, enough inhalers. And I did ask the pharmacy if they were going to deliver. And I said, I got bad asthma and they said right we'll put you on the delivery list (Julia)  No, I just carried on doing what I you know was told to do [asthma management], what I knew I’d do, you know. No no changes really, you know  (Chris)  I've been told you have to do your peak flow every, twice a day every day now, asthmatics have to. (Joanna)  And so I just started taking my preventer. Erm, and, erm, and just making sure I was being really careful… I mean I didn't have asthma at the time and when I took it it was making my lungs feel quite strange. I think that i probably used to have quite a high, and quite strong inhaler, preventer, and erm it just made me feel really bad so I stopped taking actually, after a few weeks because I just became slightly more confident that, you know, asthma wasn't a problem for me maybe and it's in my past kind of thing and erm i thought it wasn't quite as chronic or as bad as other people’s so maybe I'd be okay (Molly)  keeping up with my inhalers, that sort of thing (Helen)  I stocked up on, my fostair (Hadley)  So that was quite good there wasn't, like again, I was a bit, a bit cautious thinking again like if people are stopping working from home is there going to be a supply chain shortage. So I did sort of like, you know, I've got, you know, maybe 70 puffs on this inhaler, I’m gonna order another one just in case, because I don't want to be down to zero, start seizing up a bit, and then need it and then go ah it’s a week, I'll be honest, there's one of my inhalers if you get to zero and you may be not looking at the dial. You sort of, you take it and then like one or two days, you’re like oh blooming heck what’s going on and there’s that pressure there, and then you're looking it’s dialled out I’ve ran out, so I kind of don't want to get in that situation, of erm not having any so yeah probably quite rigid on that and having a stock which probably doesn't either GP. Yeah, I'm not stockpiling like I said just to have like one. It was the mainly the preventative one it was like, I always want one and a half decent amount left and then one in the cupboard sort of thing and then I don't know if you have like your blue inhalers I have one in the car, one of my gym bag, one in my laptop bag. Cos you never know (David)  I just stuck with what I knew. Yeah, that was kind of working (Francesca)  And I think I have to admit I don't know when it started but I, I became less consistent at taking my inhaler in the morning, just because my morning routine had changed, you know my, I was trying to take the preventer every time, like just before I brush my teeth before of course, going to the office. I will definitely have a fixed time when I brush my teeth and then that's when I will take my inhaler but sometimes working from home I would just, you know, just have a morning because I was lazy and then had a meeting first and then I might just have a quick toast and eventually it was lunchtime and I realized, oh no I haven't even brushed my teeth yet and therefore I haven't taken my inhaler and erm probably for the first year and a half of the pandemic, I've been very very inconsistent, taking my inhaler in the morning, so I always do it in the evening bed because I hate going to bed without brushing my teeth. But I would say probably four days a week I would, at least four days a week I would forget to take it in the morning, so I actually was worse about complying with my medicine (Francesca)  I just carried on, yeah I carried on using my inhalers as I always have done (Olivia)  Because I'm in the most vulnerable category so I think a lot of the aspects that have tapped into my nerves as well so I would always make sure that there was turmeric [home remedy that opens up airways] in the, in the cupboard as well so yeah I'm just trying to rationalize it really (Daisy)  I mean, I think it was all right. I always liked to know that there was a spare inhaler upstairs it's sort off… And that time when I had run out, I really really panicked. You know normally there’s sort of a cupboard full and it's fine (Daisy)  it wasn't drastic [asthma triggered by anxiety] but then I was even if I'd have had an asthma attack, I wouldn't have gone to the hospital anyways because I'm scared of medication anyway so going in the middle of a covid epidemic isn't gonna work so I just kind of rode it out and just took a lot of the blue inhaler (Shauna)  I always have to open the windows quite so knowing that I would have to stay indoors triggers the dust allergies. I remember going to boots and just stocking up on loads of antihistamines (Robin)  As usual, I got the peak flow back out and you know when you do like the erm, when you record it and just made sure that that was like the extra precaution (Robin)  Erm probably at the time of lockdown probably just taking my inhalers. Erm and then, because I live with my friend who smokes as well. She was like I won’t smoke like near you and things erm that was probably it at that point (Louise)  And I must admit, I got really scared, and I did have to say to myself very firmly, If my peak flow goes below a certain level. I have just got to phone an ambulance regardless. Because I'm just, I'm more likely to have a catastrophic result from my asthma more than anything. ... Gradually, I got a bit better, erm by sort of mid morning it was, it was under control. That really scared me and I phoned the, the asthma nurse on the Tuesday. And she read me the riot act and said, Don't ever do that again she said you've just got to call ambulance, she said if you really don't want to go into hospital because you're so worried, they will give you a nebulizer at home … it was the combination of, I didn't want to catch covid and hospital seemed the best place to catch covid (Julia)  **COVID Prevention**  I think the guidance was if you'd been hospitalized within 12 months of the first lockdown, and for me it was, I think it was like 13 months at that point. So it was, I just kind of missed out on shielding but to my mind I kind of counted myself within that, because I thought well, I it's only just by accident that i'm not in that group, erm so I did take it quite seriously at the start … And you know, we did really try our best to, my husband was doing the shopping rather than me going out, erm, that kind of thing, so yeah (Amy)  And although the kids, my son, could have gone to school as a key worker child and my other son could have gone to nursery because it was open, erm we kept them at home for all of that lockdown really I think just to try and minimize the risk of the kids bringing it back to the house (Amy)  You know erm, and it was actually, I think was Friday. Yeah, Friday the 13th of March was when I started shielding. And at that point, it wasn't it wasn't a thing, but I just locked myself away in my bedroom (Matt)  And when I come down on the train, I couldn't get, couldn't get hand sanitizer anywhere. Cos everybody had got it all. So I remember having to like Dettol wipes and use them as like hand sanitizer or something, and I was wearing a mask at that point as well, which got some really funny looks from people erm but, because nobody wore masks at that point (Matt)  And it felt really quite odd. I mean, I was already considering just not going down erm to university. erm because I was worried that, you know, I'd pick it up, but I sort of made some changes erm with my behaviours. But then I went from, you know, just being cautious to completely locking down with within like a day, as soon as, it was actually as soon as I got back home from university. I was like, right I'm not leaving my room now. And my parents were supportive of that (Matt)  see my parents, so I would, you know they'd be downstairs and I’d speak to them on the phone, as opposed to, you know, actually seeing them face to face. Erm and then when I was getting deliveries in and things for food you know I've got a little mini fridge for my room, all that kind of thing. And every time that we got a grocery delivery, it was wiped down, and that’s something that I still do now. And even though I know covid’s not transmitted that way, I'm still like well I've not really had any infections in the last 2 years so I'm going to keep it that way (Matt)  erm definitely I think it's given me a better insight for being able to make my own decisions and choices, and informed choices about how I should manage the pandemic. erm I think other people, other people in the severe asthma community that I've spoken to, erm some of them thought, you know that what I was doing was excessive. But I knew that from what I knew about how healthcare is managed how they were going to be overwhelmed erm speaking to colleagues who were qualified and saying, it's really bad, erm I thought, you know, I'm just gonna make my own, I’m gonna do what I want to do, you know, and that was that was actually nice in a way because it gave me the control. Because you know, with the pandemic there were a lot of things where, just, you didn't know what was happening, one day to the next (Matt)  I very quickly with, erm, I mean I don’t really support the conservatives anyway, but I mean with the public health messaging that was going out I was like, this is wrong, a lot of this is wrong. And it really angered me. And I thought, you know what, I'm going to let the political side of the pandemic just just go on and I'm not even going to think about that, and I'm just gonna do exactly what I know, I need to do, from my own knowledge of a respiratory virus being transmitted, you know what can I do to stop that. So, you know, I just put in my own fuse, and they were often a lot more strict than what the government guidance was so that meant, I didn't even have to think about the government guidelines because I knew that I was never going to break them (Matt)  But at the very beginning, it was just a case of me saying to myself you know I'm going to have to avoid situations, that, you know, where people can pass it on to me (Chris)  to be honest, we locked down a week before the government's lockdown. My, me and my partner, we just decided when we weren't going to go anywhere we weren't going to see anybody we were just going to keep it, because we knew that it was headed somewhere that was you know that was going to go out of control. (Chris)  And as soon as this, you know soon as the pandemic started I thought there's no way I'm going in the gym because that's going to be a classic place that you're gonna get it, simple as that. So I give up my gym membership (Chris)  Well, I pretty much kept away from everyone as much as I could, and I walked like, when we did get out for walks, we live near a beach so we’d walk on the beach where no one was. I spent most of my time at home otherwise (Millie)  I’d sanitise my hands a lot! (Millie)  Well, I decided, I locked myself down about week to 10 days before the official lockdown. I was getting very nervous, I have to go to Aberystwyth erm I did it by train once, very, I think must be the first one, very early March, and I was really nervous. So the next time a few days later I drove. And I was just looking around this big art centre there on campus which is shared by the community and I was looking around thinking, Do you know what, I don't want to be anywhere near this number of people anymore. And I did one more supermarket shop, which terrified me (Julia)  Oh, well, at first it was a real, real nailbiter because although I'd done some stocking up, I couldn't get a delivery slot for love nor money. I thought I am not going to the supermarket, I live on my own… I did ask her [neighbour] a couple of times asked her to get me some essentials. And then I found a farm shop, just outside town that also doubled up as sort of did general things and they were expanding their range. So I managed to get quite a big delivery from them .. Erm and then eventually a few weeks and I managed to get a delivery slot from, I can't remember who it was, I think it's Sainsbury’s. Er and then it got slightly easier to get deliveries. (Julia)  I was, I was going out, as it got lighter and the weather got better I was going out for walks at half past five in the morning to avoid people. And I was not going into town unless it was absolutely – Shrewsbury’s quite a small town but I live in a suburb of it, so I was only going in exceptionally early in the morning on a Sunday. And I was being extremely limited, I was seeing my neighbour, as I say over the fence but we were standing a good eight to 10 feet away from each other. And I was just, I was, luckily I was able to work from home. So, and I was driving anywhere that wasn’t walkable (Julia)  Well I knew I wasn't quite eligible for shielding. I did check the advice at the time. I was just like, just not quite in that category, which is fine. I understand, but it did make things a little tricky because I'm, because one thing I felt, especially given that I'm a single parent. My son is slightly autistic but not enough to disabled. Right, it's only, it’s mild. So he wasn't going to go to school because medically he was being treated as a normal child for that purpose. So I felt that I probably did feel like shielding maybe would be a good idea but obviously I wasn’t eligible. So, But luckily for me. And so I couldn't get delivery from supermarkets because I wasn’t eligible for supermarket deliveries. But I could have a veg box I just got in before they closed for new business because they said they had enough. Erm and luckily for me the local [inaudible] group said, Yes, we agree you do need help, and they gave me somebody to go and do some shopping and pick up my prescriptions for me (Joanna)  Another thing that did happen, the bathroom light went. And I had this absolute agonising decision to make, do I call my electrician (Joanna)  I fell down in the kitchen hurt my head, because of the back pain, probably almost certainly was damaged from having fallen down the stairs the night before. And, I thought, in a normal situation I would have sought out medical advice. I would have got somebody to go, I would have either got a taxi or got someone to drive me to the walk-in clinic to see a medical professional because I knew I had been injured and I probably should get checked out. But because we were in lockdown, I thought personally, I don't want to go. Unless I absolutely have to… And from what I’ve heard about covid, it wasn’t worth the risk. I thought I’d wait and see if I got any, if things got worse. But as it happened I was fine. but I was so afraid of covid that I could not do it. I could not do it. Unless I was absolutely desperate (Joanna)  I bought masks for when I did leave the house, I was very careful about checking my peak flow and being sensitive to any symptoms, but nothing else, in particular (Susan)  And certainly, at the very early stages, I was, kind of you know totally paranoid washing my hands like keeping the mask on, keeping away from people and it did seem apocalyptic at that point. And so I did feel much safer at home I think (Molly)  erm, a little bit uneasy because at that point, you sort of don’t know where you stand or, or what sort of precautions you should be taking. Erm it was all quite a new thing wasn’t it so it was a bit.. a bit strange to be in the dark about it but.. yeah I just followed the general advice (Helen)  I didn’t leave my village for at least two months. Sent my husband shopping. Yeah, those kind of things. I mean, he was still traveling. So, he did two trips during the first lockdown so you know, we kind of kept away from each other in the house and make sure he hadn’t picked anything up (Hadley)  I don’t think so. but I didn't go out cycling, because I was concerned about if I had an accident, that actually that would be a burden on the NHS or I could put myself at risk. I mean I cycle quite a lot. . But yeah, I kind of stopped doing that. (Hadley)  I said to my boss going like, er I'm pretty nervous about this, being an asthmatic. Can I, we were allowed to work from home like one day a month, but you're not allowed to sort of take the mick out of it, it's sort of like, if you have a washing machine or you car serviced or something like that. So I'd sort of said like, I’d prefer if I could have a week at home and my boss was like, yeah, it's fine you know you're not abusing it, you, it's a legit reason about you (David)  So, if I'm honest like back then, my best mates who were sort of my age and fit and healthy, were doing my shopping and for me and a few things like that so I, I became a bit of a, bit of a recluse sort of thing. So for couple of months of, you know, like my sister was doing my shopping or my best mate, and things like that so I probably did my own risk assessment and just thought I do not, if I get a chest infection it wipes me out, I don't want something that we don't have a cure for or any treatment for, so I probably was quite cautious, to be honest (David)  And I think I did my own risk assessment and just said, I don't want to be going anywhere near the office. Cause my lungs are rubbish and I don't want, you know I don't want to take the risk sort of thing. So I think probably at all stages I was probably more cautious than the government was advised and if I'm honest (David)  So apart from not going out, apart from, you know, exercise, like, trying to get some exercise every once in a while, we erm, we did like put away anything that came into the house like the mail we left it on the side to kind of decontaminate and we were extra careful in, when we went shopping, about what we brought back.. actually now, you know that I think about it, my husband went and did the grocery shopping for the most, like for the first lockdown period or maybe even the first four months or something of erm, of the pandemic just because he, you know he didn't want to expose me, you also you weren't allowed to go like, to the supermarket with two people it was, you know, yeah, they asked you to do it just one and then he was like well I'm not the one most at risk, so let me go. So yeah, we would, we stuck to all of the rules (Francesca)  And on top of that we were just careful about touching things. And wore maybe a mask, you know at that time you didn't wear masks but we were really trying to avoid people maybe even more than was recommended. (Francesca)  Yeah, so I remember before lockdown, erm I suggested I started working from home. erm that was probably the week before lockdown. erm I started to receive all the government letters, erm I was in the shielding category (Daisy)  I remember [my daughter] and I we went for a few walks in the woods, but it was so busy. I remember the first time we went. Ermm It was before, it was before masks, but I still ended up wearing this scarf around my face (Daisy)  Yeah, I remember. It must have been when [my other daughter] was home as well and the girls popped into the coop and I literally just stood outside, just absolutely adhering to social distancing (Daisy)  And I remember you couldn't buy the hand gels so I just sent off for a bottle of alcohol and some aloe vera gel. And some teatree, so I just made my own (Daisy)  oh god it was ridiculous, we'd watch this program on the telly how to keep your house clean, and I literally was bleaching everything. you know bleaching the handles bleaching inside cupboards ruining clothes because everything seemed to be splattered with bleach. I finally calmed down (Daisy)  I was lucky that I was able to get shopping slots, you know, in Sainsbury's because it's, it sort of pinged that I was in the vulnerable category, well you had to register. But I remember things came in in plastic It was like oh my gosh my hands and yet, it’s funny when you're in the thick of it you don't realize how you did change (Daisy)  Erm very cautious in the garden, especially when erm our neighbour’s daughter would try to jump over the fence, it’s like no no no, we still have to, you know, and as I said feeling quite cross when they would have a house full of people it was like, What are you doing, so really being careful (Daisy)  So, I was in, in and out of a lot of schools. erm, and I went into work into school to do my teaching role on the Monday, and the head teacher, just said Polly you've got asthma, I think you should go home. The guidance coming out said that people have with asthma shouldn’t be coming in. And obviously, she obviously didn't want me as well going between schools and then bringing it into our school that was kind of her motive, erm but she was like you've got asthma go home and I went home that day and I didn't go out for four months (Shauna)  We got deliveries erm my husband had to go out he was an essential worker. erm so he went in and out, erm and he would come in, strip at the door leave his clothes for the washing machine by the door erm shower and everything before, before I came anywhere near him, and for the first part, probably about three months maybe he, I didn't go near him. We stayed metres apart and we slept in two separate bedrooms (Shauna)  anything that came in the house got washed down. There's still sanitizing bottles by each door. So, after sanitizing you come in (Shauna)  at the time erm my housemate got covid quite early erm, and then we were both sharing at the same time so I to decided to stay in my bedroom (Robin)  erm whilst in the lockdown erm I did a lot of online shopping. my parents did a lot for me as well. Erm but it was just really limiting contacts and again it can be quite lonely as well (Robin)  erm I’d say just avoiding unnecessary contact I live in the city centre as well so, it was kind of nice to see when I had to go for a walk, it was very empty so that was like, peace of mind, but yeah very, very tiring I’d say. just constantly trying to be aware of not, sort of like bumping into someone who may have covid. (Robin)  It was terrifying I was in the clinically vulnerable group. But when you looked at like the list of medications I was having 800 of Symbicort, if i'd been on a 1,000 with a montelukast tablets as well, I'd have been shielding so I was only just under the shielding level. So I basically shielded because I just thought look that's so close, you know, and it was so scary and the first lockdown we didn't go out, at all, the kids stayed home, too. we've got a garden fortunately, so they could play out there. But we, we shielded. My husband would very occasionally nip to the supermarket if we ran out of a few bits. But we did, you know, online deliveries where we could, and we stayed home. It was absolutely terrifying to start with. I was like if he came back from the supermarket, I was making him change his clothes when he got home, things like that, and you know and wiping down all the shopping to start with all the whole thing of it was, it was absolutely terrifying. You know it was. It was horrible that that first one. (Sophie)  yeah to start with we were doing a bit of that erm it was like my husband would come home from the shops and I was like right wash your hands now, and then put the shopping away, I was like right wash your hands again. You know it was all that extra levels of hygiene. I think we all had quite red raw hands at the start didn't we because of it. so yeah, definitely. We were doing all that kind of stuff (Sophie)  Yeah, I was, I was following the restrictions and I was exercising a lot more to be fair cos it was the only time I was allowed out of the house (Louise) | **Asthma Management**  I think it was just carrying on with with things a bit more, being more on the ball with using the preventer every day. (Amy)  I didn’t really make [changes to asthma management], cos again it was still really controlled at that point, I was just taking my regular inhaler (Millie)  yes, I was getting my medication delivered and I was always trying to ensure that I, I had a new prescription arrive before I had to, got very far into the second of the two inhalers I've had from previous one in stock (Julia)  still carried on as normal because my asthma was very stable. I think I did slightly up my preventer but that wasn’t on the advice of a GP that was actually a work colleague who also has asthma and said you really need to up the preventer, but that was still within my, that still used to be in my asthma plan, and actually, on to what’s only very slightly and it's actually to what’s actually my prescribed dose rather than let’s start with the low one that I’m supposed to be on from my action plan. If that makes sense (Joanna)    It was actually after the lockdown this time last year that I made a real conscious effort, and that was much due to the fact that actually asthmatics were being dropped from the priority list. And at that point I knew that actually my asthma’s not controlled, I need to get it sorted because I need to protect myself if I’m not gonna be prioritised for a booster. I mean, I can't, I can't be living like suboptimal (Hadley)  So, yeah, probably quite vigilant with the triggers like, like if it's sunny outside. great. If it's 40 degrees, be cautious. If it’s minus two degrees, snood on, hat and be cautious. Be a little bit more cautious that I might, I don't really want to go to the doctors and I don't want to go to hospital. So, probably quite, vigilant (David)  I think, because I was still having the regular check ups. Erm just making sure I remember taking, If I don't take my preventer, I sort of panic so I make sure I've always got a spare one with me. You know I’m sure it would be okay but I guess because you psychologically and physically rely on it (Daisy)  I had my asthma review, my reviews have continued all the way through, so I had my asthma review erm and the guidelines had changed that adults should have a steroid inhaler. And I was using it lot more so she said that's, that was the best course of action for me. I did try and do yoga a bit more breathe in more to try and calm me down. But that wasn't helping as much as they had been (Shauna)  Oh no it completely went everywhere [asthma management]. Erm I think because I was stressing about it, like one week took my inhaler, inhaler too much that I think I burnt my chest it was just, yeah, I had a really bad time. Erm, yeah, it was just, not very well managed but I think that came with the stress. Or I didn't know how to manage it during covid (Robin)  Yeah, just kept taking my inhalers, and booking for my asthma review, things like that. (Louise)  **Covid Prevention**  I didn't go to a pub for example for quite a long time after the lockdown ended because I just didn't particularly feel comfortable doing that, and didn't want to. So. Erm yeah, So we've definitely kind of erred on a side of caution as a household in terms of following the guidance, but kind of going a bit beyond the guidance as well a lot of the time (Amy)  So, erm this was around the time I actually started the antibody treatment. And erm I was thinking that I would actually put it off. Erm I was thinking about cancelling, you know, having to go to the hospital because I mean I lived in [place name] and the hospital, that was dealing with my asthma management was [place name] erm so quite, quite a big distance, erm and i was just a bit like I really don’t want to go to this hospital. Erm and so, I rang them up and actually you know one of the, one of the asthma nurses ended up ringing me back, erm one of the asthma nurses had actually seen me on EKMO. And she, she knew me quite well. Erm I had a very good relationship with them. erm, and she was being like you know you really do actually need this, you need this treatment. Erm so I did, I reluctantly agreed (Matt)  So, I'd already made the decision that I wasn't coming out until my vaccine, so I was completely staying in the house (Matt)  erm so that's where we started to get deliveries for groceries. So using like the priority order in on Tesco and things. We, just basically everything was, everything was done by delivery (Matt)  But, um, you know, we also would, would go out on drives as well. erm at that point, with like the windows completely, completely winded up things. And erm that was good. So, you know, we could really see some scenery, but we were still being very very cautious because we knew that my risk had not changed at all from March to July. July, August time, because we still didn’t have the vaccine at that point (Matt)  And yes, I was worried about it actually, because I was thinking Jesus, you know, you know if I start going out if I get this then my asthma’s not under control. I don't even have that, you know, but then we both made the decision, look you know we're not going to go out, if we don't have to, you know. So erm luckily we're both in a position where we can do that. And that's just what we did. And then as far as my asthma, yeah I was worried about my asthma at the time but I thought well I’ll get that under control but I'm not being kind of, I’m not having to go to work every day amongst people that might have the virus, you know (Chris)  erm sometimes I did have to like, brave it and wear a mask. And just like, distance as much as I can. Cos you’ve gotta get like, up close and personal in my job cos you do like personal care n things, so yeah (Millie)  so I had a birthday lunch outside with my brother and his partner. And I'd measured, I'd had my neighbour for coffee fairly soon after we, into the garden I hasten to add,  but before I did so I measured it, so I knew exactly how far away the chairs were and pointing them so that we weren’t facing each other directly (Julia)  erm I ambivalent, because looking back actually I was probably safer than I thought, but erm I was still wary, I thought not only did we lockdown far too late, and not holding off, but we released as, as has become the pattern we opened up too quickly and too much. I thought I’m not desperately happy about this. I did see more people but in the very careful situations, and more generally, I thought, well, if I can avoid going to anywhere that's crowded or inside or I don't have to that I’m going to (Julia)  The washing machine yes I had to have a new one. and I knew it, because the washing machine was so ancient that it had just gone. I paid extra for the delivery to leave it at the door, they couldn’t do it, so my delivery people they tried to socially distance. So they were very, they were good. They just came in the back way they didn’t mind. We tried to maintain social distance, I can’t remember if they were wearing masks but they did try to social distance. The fridge freezer guy was in a mask, gloves, he was totally Social distanced. The washing machine people, they did try to social distance, they made the effort and I was totally happy with that. Except they couldn't do the installation so I had to get a plumber, to come and do it. Who, I was a bit worried because the social distancing was terrible and he had no mask on. So I was worried. But, he was very quick, which is why I got him because I knew he knew exactly how to install it (Joanna)  Well I was, we went out for walks. I think I still had someone doing shopping for me to begin with. Erm and then she really encouraged me to try and do my own shopping so I decided I would go to the groceries, you know corner shop growth corner shop but I didn’t, it didn’t, I didn’t feel ready to go to a supermarket. I know different people feel safe in different places. And I was only going to the corner shop to get butter and things like that (Joanna)  Erm I had to take my car for its MOT. I by walked back from the garage, for a couple of miles. And I walked there again to go and pick the car up. I was not going to go on a bus. Absolutely not. I still haven't got the bus yet. (Joanna)  I was finding it scary because I knew cases was still high, it was all around and masks made me feel a bit safer, so I felt okay meeting outdoors a bit but it was still, I felt nervous and I didn't want to do things I mean I went into very rarely went into, into shops I went into the charity shops a few times, but only because I was like, actually I really want to take some, to take donations to the Oxfam shop. not because I particularly wanted to, you know and I wouldn't stay in and browse I’d just take my donations in and leave (Joanna)  because my son, so during November 2020 my soon was allowed to carry on doing his piano lessons because he's autistic, and he had, my son had an exam and I had to, which I just never normally do I had to drive, It's around the corner from [place name]. Now normally, I could just hop on the tube and go up there, but I thought there's no way I’m going on the tube, no way. I’m going to have to drive into the centre of town, which I’ve never, I hardly ever drive there. I’ve gotta work out, where on earth do I park, how do I do this. And I had to do it. Drive him up to town for his piano exam (Joanna)  It was, it was better, but we were, because I was still effectively working online at home. erm it was good in terms of not worrying about, you know, that I can't leave the house at all, but I was pretty slow to do anything more than going to the grocery or you know, unless it was absolutely necessary, leaving the house (Susan)  although there was once or twice where I went to a restaurant which was indoors, but it was with people who I knew had, were fine. So, yeah (Susan)  oh it was quite testing, I mean I was, I actually went back to work a couple of days every other week. erm, but I was keeping an eye on cases, making sure I was wearing a face mask, making sure there were limitations on how many people were in the office and it was like three or four so that felt, okay. I wasn't going shopping If I could help it, I’d still get my husband to do it. And not, not really going out and I haven't been to a pub inside. Since all of this, which is a long time. Yeah, I did go back to my cycling group, but we were in groups of six so that felt okay and seeing and having cups of tea outside. But not back to normal (Hadley)  So im like again in my head, Dooms Day thinking, I’m thinking yeah if there's 30 people in that GP surgery one might have it, well sod’s law, I might get it. And I haven’t got a vaccine, so it's gonna, it's going to topple me over quite so yeah I was very much like, to be honest you know you said about the asthma review, they wanted me to come for face to face and I was like nah, I think I rang back and just said, you’re alright, and they were like no no no just come in, like, no, you're alright. And they were like no, no you need to come in, and I was like do I really, do I really need to come in and like yeah, so it sounds bad but I was kind of like, do I really need to come in, to discuss the maintenance of my asthma that I've had for 39 years (David)  Yeah, again, again I'm probably doing my own risk assessment and go tighter. So I'm trying to think I'm trying to think. The end of the first lockdown I think was around my birthday, I think we were at a thousand cases a day and we decided, and I tentatively sort of said maybe we’ll go out for a meal for my birthday, there was me and like five others, but like, say you're allowed people in your house. I wasn't doing that. I would be like to my schoolmates let's, let's go for a walk around the park and we have an hour’s chat, maybe grab a coffee and walk, football was back on so you could socialize that way. Yeah, well, yeah maybe like if I was socializing it was always outdoors, like at my parents garden, they would come to me (David)  So, you know, so, a friend of mine would maybe you know, come down and sort of yeah we'd say go for a walk and keep a metre distance and things and just, yeah, I'd say, super cautious until I got those two jabs in my arm which sounds bad but it just, it was sort of like, I think it was, if it was anything else I'd be like, I can be like I could bat it off but it's a bit like, at the time, it was said asthmatic need to be cautious and yeah you just have that drummed into you. Yeah, so it was kind of like well, and I'll be I'll be honest I'm quite a, I'd say a cautious sensible person. I try and limit what I drink, I wouldn’t say I never let my hair down I'm quite a, I just like to be in control (David)  And there was no pressure or peer pressure, but when I was saying no to going ah let's go to the cinema. I'm not sat, sitting with 100 people randomers. And they were like, oh, what's your problem. What's your problem. And I am quite stubborn I was just like, Well, honestly you three, go, and I'll just stick, you know, miss this one out, and I stuck to my guns I probably, I wouldn’t say it affect, I wouldn't say it like, it didn't end any friendships, but it sort of put a strain cos you’re then slightly out of the group, three are off playing golf, three are off to the cinema, three are off to the pub. And I'm just going are we having a zoom call and it's a bit like oh we’re allowed And I’m a bit like yeah. So, yeah, probably, like again, own risk assessment was just like, not, not, it's just not worth it. No, no (David)  To be honest, it didn't change at all. Like, I think, like my household we were very hesitant to to trust what was going on and so we just we just kept to ourselves and continued to like stick to quite strict regimes I I don't remember us changing our routine, until, you know, much, much later on like speaking to Friends who’d say, oh we were out for a meal yesterday and we were like, no way like that, we hadn't even thought of whether that was acceptable like or safe enough or not. Erm, and, yeah, it's not only to shield me but also just generally to shield those who are vulnerable, we really took quite a like a strict line and we still limit our social contacts quite a lot which was quite easy because as I said, we haven't actually lived in Portsmouth for that long, so we can actually have a lot of contacts that we could miss. Erm but yeah we didn't go out, we didn't really go anywhere, apart from, you know, maybe for a walk when we, to a place where I was quiet but yeah (Francesca)  There was a time in the summer of 2020, we went abroad to see my in laws in Greece. Erm and that was maybe the one thing I was glad about like not, it wasn't Freedom Day, but it was the lockdown easing at the time. The first lockdown. That we could travel again and so we went we went to Greece because it's warm and you can spend all the time outside and you don't really feel, like it was very safe cases in Greece was super low they had like five cases a day or something at the time. And we were out in the countryside where there's nobody and, you know, still sitting far away from everybody. But it felt reasonably safe, and I was glad that we could go there (Francesca)  And, I mean, it was a concern. When we were allowed to reopen our business we did, but most of the time I stayed at home and my husband went and manned the fort. erm we started wearing masks, before they were mandatory. That was a bit of a concern you know having asthma and having something over your face. And I remember at the time I I sew, one of my hobbies is that I sew all my own clothes. And so I took up making masks. And to get used to them, you know that that concern of having that over your face while you're trying to breathe. And I remember, Five minutes wearing one then 10 minutes and then building it up to get used to it, so that I could on occasion go to our business and go and work and be comfortable wearing a mask. So that was, that was quite a big step in itself, you know, but even, even then I still, I still wasn't going there every day. Erm you know, just because we decided it would be safer for me to stay at home (Olivia)  Yeah, very much so I haven't, we haven't socialized at all, we've not been a dream for me or anything from since the start. So the only people we really mixed with were the other people who come into our business. And we had really strong strict restrictions on how many people can come in the mask wearing as well so you know we've, we've done everything we can to try and protect protect ourselves (Olivia)  I mean as soon as I could go swimming I did, because erm apparently the virus can't live in chlorine (Daisy)  you know, but erm yeah I'm becoming quite fixated with always having a mask … And I guess always having the hand gel as well (Daisy)  because it still was limiting I still work from home. I wasn't really going into school, I didn't have to go into school for my other job until September. erm so I started, and even then it was only in sporadically and it was one day a week if I was in (Shauna)  still wasn't going back to my, I did my dance class online. I felt then that I, from then on, really I've only really walked it through I've barely been able to dance it through, my yoga was online, so I just I was still, I still hardly ever went out (Shauna)  I do go out more now, and do things but at that point, I literally was dragged, literally dragged into the shop. dragged to do anything, we went to, and I wouldn't go near my mum and dad we, we've all been really strict in our family. We've, like if they came around, and we were planning for a day when it was sunny outside they only came in the house to use the toilet and I had everything cleaned. That's one thing that has, we were thinking, my asthma nurse, since covid started I clean everything, top to bottom all the time, and we think that's what's affecting, because I'm now using lots more chemicals (Shauna)  if we were going down to my mum and dad's, erm or to his parents or whatever, we would take the motorhome, so I had my own toilet. So I wouldn’t even need to go in their houses or anything, but if you stopped off on the motorway I used the toilet in the van (Shauna)  erm again, I just kept myself, kind of limited and didn't really go out with like my social group, unless it was outside (Robin)  the big thing for me was the kids went back to school at the end of that first lockdown for 3 weeks, or they had the opportunity to. And my, or some children did, and both my children were in year grades that were able to do that, and I kept them off. Erm because I was just terrified with them going back to school at that point (Sophie)  And then we had the summer holidays where we started to go out but we kept to outside things. everything was outside we didn't do anything indoors. We, we live quite near some meadows right behind our house, and we went walking around the meadows a lot, we walked every metre of those meadows. It was all that kind of stuff that we did but I still avoiding things like parks and play areas, because I didn't want to be in crowded spots. So it did, that summer holiday, we did a few things, but they were everything was outdoors we went to a castle but it had limited numbers, you had to check in track and trace you had to wear a mask. So every single thing we did was outside, fresh air. Erm absolutely nothing indoors. In fact, we still haven't done a restaurant or a cinema, or theatre, or anything like that. Even now (Sophie)  No, erm not really apart from just avoiding like people! Just trying not to be where people are gonna be breathing on me really (Sophie)  we'd meet we'd meet people in gardens or in public parks. I still didn't have people going into the house and whether that was legal or not I can't remember, but the fact is that we didn't. (Sophie)  we'd meet we'd meet people in gardens or in public parks. I still didn't have people going into the house and whether that was legal or not I can't remember, but the fact is that we didn't. (Stephen)  erm. I think just really limited our interactions with other people, any kind of mother and baby things that we went to, they were all the ones in parks and outdoors that. We kind of really just deselected anything that was indoors even if it was a rule of six in inverted commers. So. So yeah, there was. There was still that restriction. And I know that I found it hard my wife found it really hard just that sense of isolation and also that feeling that you were, that I was causing other people's lives to be more difficult because we had to do things to take into account my asthma (Stephen)  erm, I wouldn't say it changed much I guess just, I was more like on the ball with my vaccines. So like my flu jab I made sure I got that and then as well, (Louise)  it’s funny because by that point em and my partner were going well we could really do with going to the shops, but we use the strategy of going okay, we have some of your retail parks that were open till eight o'clock at night, so why don't we go quarter past seven. The last 40, 45 minutes is the quietest is going to be, you know, let's, and you know we were very judgmental, the same happened when we wanted to take our children to parks. You drive to it and you make a decision, if it’s too busy you just say I’m sorry guys we need to go somewhere else (Annie)  yeah the rule of six was good to get people in the garden. even when we were allowed people in the house we didn't but it was the summer, so that was not really a need to, if somebody had to use to the toilet that was fine. But we were much more hyper-sensitive to it. So before someone came, i made sure rather than going through the back of the house they came through the front of the house because the toilet was right next to it. So, the door handle was wiped on the bathroom door and when they leave the whole bathroom was cleaned, new towels. I’m not saying we do that now necessarily because we know much more about touch, but you know we were very kind of hyper sensitive to these things and within reason, you would try and keep visits short so you didn't need to do that (Annie)  yeah for social interaction. But we were limited because when people wanted to loads we just didn't want to do it. we were lucky my daughter did get to have her, her birthdays at the end of July. So we did manage to give her a little party in the garden but again it was select. It was my parents. It was my best friend with her two girls cos I knew they were kind of quite sensible, and it was my son's friend with his wee sister because they’re kind of similar ages. And that was, that was it, it was really hard. And again, all the adults were faced out, you know, and things like that. It was nice to have that again but it was in the garden and it was very, it was people I knew I could trust that were not going to be going about galivanting, I knew the situation we were in and if there was any doubt then I said we're not coming. (Annie)  when my husband had to return to work we did the same thing. Soon as he came home, everything in the washing machine he went for a shower at that point, and then we reduced it to just do a quick wash once the science came out but you just, you just, you don't know what’s clinging to your clothes and I still do it when they’re at school but im of the opinion that there’s no restrictions in primary schools. Washing their hands and a bit of sanitizer’s not going to help stop a bug in the air. So, just in case it clings to their clothes and then it's sitting in the wash basket or something (Annie) | **Asthma Management**  No my asthma management stayed the same (Joanna)  not really, I’ve just kept it the same, I just as I say I do use more of my salbutamol as a rule (Helen)  Again, unchanged, I was probably still quite loose with taking my inhaler in the morning but very irregularly, but I took it every night (Francesca)  **Covid Prevention**  So they were pulled the last three days because our kids were meant to be in school till 22nd, so I did pull them from the 17th, but it's that kind of thing. It's the change of the risk cos I think a lot of people who are worried it now, don’t adapt to the risk. (Annie)  Yeah, so I mean I I still wear a mask erm in shops, and if we go to a restaurant i'll wear a mask until I’m sort of sitting at the table, and that kind of thing. Erm. yeah. If I was good if I was if I went into a pub and it was really busy, I wouldn't feel very comfortable with that, going and sitting at a table somewhere I don't mind so much, erm I wouldn't generally choose to do something like that I don't think but if if a friend was organizing something, I probably wouldn't say oh no i'm not going I would go along with it, but it wouldn't be my you know, my first, my first kind of, I would certainly be aware of how busy somewhere, is, and if someone was really crowded I would feel a bit uncomfortable with it. (Amy)  Yeah, to an extent, and certainly early on, when you could first start going out. I did kind of say no to things because of because of that, and then kind of, I guess, relaxed a bit more as time went on. (Amy)  I’d have my own bottle of alcohol, and they'd have theirs, so you know, we weren’t sharing and things, or that I was using quite a lot of hand sanitizer. erm there was no comments about that, you know that that was just seen as completely normal. erm, and so it felt like you know just going, going back to pre pandemic life, to be honest. (Matt)  I mean the only thing that I haven't done, I used to go clubbing quite a lot. Erm and I don't think I'm ever gonna go clubbing again, erm so that’s the only thing that I’ve not really gone back to. But like, you know, eating in a restaurant or going to a pub. Erm I will, er I did do that a couple of times last year, but I'd always make sure it was outside, and nobody had a problem with that and I think it's just because it's the friendship circle I've got, that all felt so normal to me (Matt)  I did just because I live in Cambridge now, so erm there's loads of wide open spaces. Erm so, you know, the only risk is when we're going from my house to those wide open spaces, but you know if you get your timings right and you just walk on opposite sides of the road to people. It's not an issue (Matt)  it [covid avoidance behaviors] carried on, it carried on that are you know as far as, you know, we carried on what we did. You know, we didn't change (Chris)  Friends that I knew had started getting back together again going to pubs together and then I was saying sorry I'm not going (Chris)  Um, it did, it affected it negatively I'd say because erm with certain friends it was the case of, will you come to the pub? Well I don't want to. Why? because there's a fucking pandemic going on. (Chris)  you see I was trying to protect myself but I was also trying to protect my kids, you know we home-school basically so we were able to keep our kids away from things a lot, erm the we did it ourselves you know, so they just zoom called their friends who are also home-schooled you know so they kind of used to that type of thing (Chris)  Yeah, we just carried on as if we were in lockdown. You know, erm, that lasted a while really until we felt that we were you know we could go out and with masks on (Chris)  like, one of our clients, I work for a lady with cerebral palsy. And she got covid. So she like, we were back in like, even though the restrictions were all lifted, we had to go back to wearing full PPE and all the staff were testing (Millie)  We were in like masks a long time at work and just keeping my distance wherever I can. (Millie)  It was terrifying. And er I'm sure you've heard this a lot. The so-called Freedom Day was lockdown day again for me, I'd start doing click and collect from the shop, where I’d go in and talk to them because everyone was wearing masks. I did a click and collect in mid-August, after all of the restrictions gone. And I, I was horrified because there were people in there that, even one of the people in the members of staff wasn't wearing the mask. Luckily I was wearing a mask and I was in and out quite quickly. But I said to them, I'm not coming back, if I do a click and collect again, I’m going to wait outside to get it, actually I did this the other day, I said I will phone you when I'm outside, and you can get somebody to bring it out to me. And it really meant that, and I also did a click and collect from pets at home, because I couldn't get something I wanted online. And again, I was just so terrified by the complete lack of any, er even basic common sense. We're still in the middle of a pandemic. I did, I was absolutely horrified and terrified. And it has been hugely detrimental for my mental health, and my feelings of security. Why couldn't we of just kept some of the basic mitigation measures? (Julia)  And actually I went to Aberystwyth get stuff from my office couple of times. And the last time was in early September and I was saying to colleagues I’m, we had lunch outside, and nicely distanced and I said to them I feel much safer the side of the border where you've still got some of the basic mitigation measures in place. Actually the university were very good because they said erm that if, you know I've actually got a fellowship at the moment so I've bought out my all my teaching and admin which is the academic golden ticket (Julia)  I spent about two weeks barely leaving my, my garden, I’d occasionally go I I live only a couple of minutes from a recreation Ground. I’d occasionally go there, but I'd have a mask on, and only take it off when I was quite secure that there was nobody can come near me. And I wouldn't do a route that I've done before, because there are two really narrow pinch points. I thought I don't want to come face to face with somebody coughing and spluttering at me. Yeah, even if I'm wearing a mask. (Julia)  I had to have emergency repairs, I discovered that my living room floors were about to fall through, the people I bought the house from it had had a nice new floor put over a rotten woodworm infested floor, and then put a full ceiling up in the cellar so you couldn't tell it, and it was anyway so I had to have a builder and a carpenter in couple of weeks back but they’re the people I have to do renovations the house and they were very careful. They didn't tell me until they had been there three days they were taking a test every morning. And they were being very careful and they’d sealed off the bits of the house they were working in and they were wearing masks for anyone near each other but they’ve got to come back in early January to take the ceiling with the seller down which is underneath the room so it can get air circulating actually spoke to the builder this morning and said can I put you off and he said The problem is, we moved another job to fit you in and I'm not sure if we could fit you in again for quite a long time. So at the moment we’ve left it at, they will take a test every morning. They all wear FfP masks, when they first come in seal up the rest of the house and I will just be incredibly careful and stay away from them (Julia)  I mean I’m still not socializing a lot, but I did actually, last July, first time since the pandemic, I actually went out for a coffee. And admittedly I haven’t done it since, yet. And it was it was outdoors and it was a special reason, I and didn’t even know if I had been invited for coffee, I was giving a [inaudible] to an acquaintance of mine, I'd made patchwork felt and I was giving it to an acquaintance (Joanna)  I still had not got back to going out as much as I did before. Erm I am glad that we've been teaching on campus, and that feels relatively safe. (Susan)  I didn't do anything enormous like go to the Christmas markets or go to a big outdoor concert or a concert for that matter. Erm, I was still relatively cautious about, you know, erm, I'm happy to go to restaurants I'm happy to go see friends and erm sort of ordinary socializing. Erm but I'm not ready to be, I did travel to London and I wasn't thrilled with being on the train, or in a crowded train station. I was still more cautious about it. But I think, I don't know how much of that is worrying about my health and about asthma and how much of it is just that it felt weird after 18 months, to suddenly be in a big crowd again. So yeah (Susan)  erm yeah it was a welcomed change I still continue to wear a mask, erm for my own safety and other people (Helen)  I: you mentioned one of the main reasons why you wear a mask is to protect the people.  P: Yeah, because when you have someone vulnerable in your family and what have you, you sort of, I sort of think about them and think, Well, you know, what if that person has someone vulnerable in their family or what if they are the vulnerable person, I just would, you know, I would hate to be the person that passed anything on to them (Helen)  So this would have been. So to work out, isn't it This was when it was last summer, summer 2020. Summer 2021. Okay. either way, I didn’t change my behaviour that much, I was wearing a mask everywhere I go. I mean, I've been to a few restaurants, not many but would wear a mask to go into a restaurant or when getting up from the seat to go to the toilet. And at the supermarket, that behaviours continued (Hadley)  I mean I've been outside, but not inside, um, I mean, restaurants I’ve been to a couple of times but cancelled, like cancelled reservations when cases have been looking a bit uncertain. I mean at first I’d like to go to restaurants, erm after the last… yes, kind of May last year but that actually felt quite safe because new cases were low, but if you’re in a small like posh restaurant you know that actually, that's probably the safest time because we’d only just come out of a lockdown (Hadley)  Some of my friends were like if the pubs open on Monday, Sunday night they’re at the pub 12 o'clock on a Monday, straight on the pints, and I was like, no, no you’re alright. So now, again I'm trying to socialize I'm something that's go for a walk let's play golf let's have an outdoor tennis game let’s have.. And again, I'm the only one in the group, saying that and the others are going, well we're going to the pub, so you do feel a bit isolated. Like, yeah bit of an outsider or the one who's being a bit boring or a bit cautious but I was quite happy to do that, I'm a bit stubborn so I thought yeah (David)  It sounds mad, but whenever I socialize as soon as I come in I have a bit of mouthwash, I don't know if it's gonna do anything. But there's a bit of logic there saying well if I catch on day one, maybe I can sort of, you know but, yeah, we’re not talking lie Donald Trump but it's like I say that allegedly there's been some studies into like I say alcohol mouthwashes (David)  So I bought some from like Holland and Barrett and so think I just take like one or two tablets a day, and it just sort of said, people who have really bad covid, maybe had low vitamin D. My mind was like not particularly sunny in the UK. So yeah, just little things like that (David)  I think life did get a bit more… varied, let's put it that way. I remember wearing my mask, like being quite strict about wearing a mask, even outside, even when nobody else was wearing a mask. Erm, I continued to do that because it just seemed the right thing to do, it made me feel safe, but I also still, I still thought that it's the right thing to do to protect others, which I, what I, something that I found in find interesting is now with Omicron, it's a lot more about wearing good mask to protect yourself, whereas you know for the second half of 2020 and the first half of 2021 it was wear any mask, it doesn't matter, to protect others (Francesca)  But always having my mask with me I think that was kind of. Probably the biggest impact, or the biggest difference to a normal life (Francesca)  And erm we had at our business, we kept masks mandatory. We would not let anybody in for any reason if they wouldn't wear a mask. Erm, thankfully, our customers are amazing, and we haven't had a single problem with anybody. So, you know, everybody is respected, we've explained that I have asthma, erm and that we need our customers to wear the masks, and keep their distance and let people in with limited numbers (Olivia)  and it limited us, I mean, I mean we, we have groceries delivered. But obviously, you need to get a few bits and pieces in between, but we're really limited, when when during that time when masks weren’t mandatory, we did not go into the supermarket so we just managed with what we've got. It just felt like too much of a risk (Olivia)  mean I felt like I wouldn't ease my restrictions. So, I was, I would pop into town, I would go swimming. Have a poodle around etc. And, yeah, go to different places so my world began to expand a little bit more, but I would still keep distancing, erm still wear the mask still use the hand gels. You know, as I said in supermarkets still clean the little baskets. So, I guess my, my way of looking after me hasn't changed (Daisy)  I think it's not just about me it's everyone else isn’t it. I never just see it as me. Erm I test regularly like anybody especially seeing Dad in the care home. I'll do a quick test weekly, more and more like the event yesterday we had to do the test, show our results (Daisy)  Clean my hands regularly. Social distancing, but I think it's just amazing how I would say for most people, they believe it's gone. Cos like yesterday I was on the tram. I was absolutely, I mean me and my colleague were the only people wearing a mask and the tram was standing room only. You know, so, and we're still I think, the death rate is still like 1000 a week (Daisy)  Erm and there was also a point where one of the service users, who's also a staff member, refuses to wear a mask and someone wanted me to manage them and I just said no. I said I'm not going to do that (Daisy)  if the bar was too busy or anything I'll just go somewhere a lot quieter (Robin)  erm I remember avoiding a lot of erm parties, erm I was going home to see my parents quite a lot instead, and just avoiding social contact, again it was doing it in my own time rather than just taking my mask off the next day erm I started slowly going back to like yoga classes. And I remember walking into my yoga class and I was the only one wearing a mask and I was, I was just thinking like, am I just not the normal one? But yeah, just like easing my way back into it (Robin)  I knew it was something that I had to do just to actually like put myself back into the real world a little bit so I started going into like supermarkets erm actually taking my mask off like in a supermarket. Being surrounded by people, erm but again it was a lot easier because you had the information and people were talking about it a lot more compared to like the beginning (Robin)  Yeah and hand sanitiser, I’ve used so much hand sanitiser (Robin)  I don't think I still went into a shop at that point thought, I think my husband did. But I think we maybe went into the town centre once on, like a really random, quiet time of day, like a Tuesday morning or something (Sophie)  Well. It was good. it was good for us to be able to do stuff, for example, like we were finally able to take my daughter to go and see my parents, to go and have a small holiday, erm and to be able to get away from just our local area was great because we felt very closed in. very scared though cos a lot of my friends came down from the Scotland England game for the Euros football game, and pretty much all of them got Covid (Stephen)  We went, we went on holiday within, within the Uk. erm we went to Butlins on a family holiday, erm fortunately the weather was great so we didn’t, we did more stuff outside, you know so you'd be the only person wearing a mask, but erm and you start, initially you start to think well if you won’t wear a mask in a situation like that, you're likely to pick it up and that’s your own bloody fault then isn't it. It hasn't made me a better person, put it that way (Stephen)  No, I I don't have much of a social life, and didn’t then to be honest, erm did we meet people? We still were very much kind of meeting people outside in gardens. That sort of thing. My wife went to the theatre a couple of times. which I didn't feel comfortable doing but erm. I don't think much changed and unfortunately just with other people's world just going back to normal and that sort of thing. (Stephen)  erm yeah probably like the opposite way of what people would want because I just thought like , I've already had it and it was when Manchester was in top tier so I may as well socialise (Louise)  They had to make sure that if you didn't feel safe that they would have to take it into account and I think there was quite a lot to protect you at that point and it was a choice, and I still think most people in a similar situation to myself, still make those choices. You know they have chosen to go, no I'm not gonna go to the cinema, no im not going to a concert I mean, I happened to win free tickets from sky TV to go and see Steps, I was like, No, of course I'm not going to see them, I thought, there's no way I'm going. And interestingly, two days before it came out, we got an email saying you do not require to show proof of a vaccine before you enter the concert (Annie)  September, October that's why we went to the cinema, because it actually was looking like yeah, maybe, maybe there’s a light round the corner for people like myself who was hiding away, and yes, we didn't just go to the cinema I did check our six local cinemas, checked which was the quietest one, but it was still progress! (Annie) | **Asthma Management**  I've just checked and there are only 52% emergency ambulances in my area, arriving within 20 minutes. Erm and the average waits in an ambulance to get into a&e is an hour and a half. So, I really don't want my asthma to get to a stage with my asthma where I need emergency treatment because I might not (Julia)  **Covid Prevention**  I think that persistent fear of death and disability through having contracted Covid would be something that, you know that’s why I want to get it erm plus the fact that for this stage it started to come out that Covid actually affects multiple parts of your body and can do for a long period of time to come. So I wasn’t in any way hesitant about it (Stephen)  Oh absolutely, because I just knew that, that that's what was needed. I mean, I'm, yeah I am very cautious I will only take medication where I need to, not because I'm anti vacs or anything like that. But just cos I've had so many, like with my allergies and what have you in the past. I have, I have to be very careful what I take, and it has made me very cautious, I do have a phobia of doctors. but I know, I know that, I knew I needed it. So, it's you, you weigh up, all the things. (Shauna)  but because I’d been vaccinated early the first time my vaccination had been, I can't remember how long it had been then, but it it had been like 7 or 8 months and people were getting vaccinated who were slightly older than me but weren't high risk. And then, because I was under 40, I wasn't getting vaccinated for a long time, so that that was quite a worrying time, and I did cancel quite a lot, of social things then, I was basically unvaccinated because It'd been so long (Amy)  I think I had to call my GP because I wasn't that, I didn't get an invite I had to call them when they were doing Group 6, and said, you know, I haven’t heard from you and they said they'd missed me off the list or whatever so I was kind of proactive in checking if i'm getting it, and erm asking for it when I was eligible (Amy)  Yeah, it definitely made me want to have it because erm I know that the last virus I got triggered my asthma, and like I say, put me in, put me in hospital for 3 days. So, erm I was always going to take whatever I could, whatever I can to reduce the risk of that happening, although when I got covid, actually I was, I was quite all right with it. But you know, you don’t know do you, what's gonna happen (Amy)  Yeah, yeah, I think I certainly felt more confident erm to go out to sort of crowded places, and actually so like before I had the booster I didn't go on my works Christmas do because I didn't want to, that's obviously before Christmas last year, when it was all going up again. Erm I sort of chose not to have that because I hadn't been boosted. whereas if I’d had the booster I would have gone to it. Erm. So being vaccinated would have impacted on decision that I made at that point (Amy)  erm, I mean, I didn't have a difficulty to get in the first dose or the second dose but getting my third primary dose was an absolute nightmare. erm so, so they released the erm guidance to do it on the first of September, and health secretary at the time was like saying oh aren’t we brilliant, you can get your third dose – it took me six weeks to message, my GP, I spoke to my consultant I spoke to my asthma nurse, I spoke to vaccination centres. I then actually had to speak to the clinical commissioning group. erm to say to them, you know, I quite obviously meet these criteria I need my third primary dose. Erm and in the end the CCG turned around and said to me, like oh yeah just, just go and you’ll be able to get it. Erm and I got booked in via the government website for a booster. So when I turned up, I said erm, you know, I need to have this as a third primary dose here's the guidance, and the clinical erm person at the top of the clinical lead that day was like, we're not doing it for you as a third primary dose, you can get it as a booster and that’s it. And I was a bit like, Well, I was in two minds because I can either get dose now, but then I know that I'm never got a booster dose. Or I can turn away and get it as as a primary dose properly. And so I left, and I was really quite upset at that point, because I knew that I needed the extra protection (Matt)  So after my first vaccine I didn't make any change at all. I still kind of shielded in this flat. And then it was after the second vaccine, two weeks after I was like I've got protection now so this is where I can start meeting with friends. So that was the first time that I actually met with friends after about 9, 10 months of living on my own. And so yeah, it was nice seeing people. (Matt)  I put myself forward for trials, actually, erm when the vaccine was out I didn't get called up but you know I was there. (Chris)  erm, I didn't you know take the foot off the brake as far as you know, you know looking after ourselves, and making sure that we didn't go places we didn't really need to. But, um, I was excited because I thought well hopefully this one step to getting back to some sorts of normality, you know (Chris)  [after vaccine] Um, we started going out so you know we started shopping rather than getting deliveries (Chris)  I still felt quite wary. But I was still like, I was trying to get out and do more and, now I know I had that little bit of protection from having the vaccine (Millie)  I felt like safer that could do that. I knew I was still at risk, and I didn't sort of get like too close to people, and it was just nice to know that I was a little bit safer from having the vaccine and felt more protected, that I could go out and do them kind of things now. (Millie)  And it just drives me mad and again I did get, because I'd had my vaccination in group 6 I got my booster. And actually I had my booster and I thought well this is looking, it looks so good for protection. When I'm about two weeks past the booster I’ll start doing a bit more again (Julia)  I was meeting more people, outside and being careful, because I thought well vaccine does give me protection. It's not foolproof of course not, it never is, but it does seem to, to, and if I did catch it it would seem, hopefully make it a bit milder. And then with the booster, as I say, I thought that the results from the booster against even delta was so good. I was actually getting to the stage where I was thinking of doing slightly more, even maybe having a neighbour inside with windows open. And then omicron hits (Julia)  I felt like I could go out more, I think, also, I felt like again I could go see friends who were more vulnerable. Yeah, and my friend who was going through chemo was very vulnerable at that point, and it was a relief to be able to go see her and help her. So, I felt safer from other people but I also was less worried about catching it and taking it, and giving it to someone else (Susan)  I think as soon as I got the first one I really did become quite slack, at sort of protecting myself. (Molly)  erm I was little more confident about seeing friends and going out, but I wasn’t going, you know, I still, I was like I said I've not really, I’ve not gone to a pub, I've not really been to restaurants... I was happy to go into the supermarket. So I did that whilst, before my husband then got vaccinated, so I took over that responsibility (Hadley)  But now [after vaccination] I'm like, well, you know what, it’s so and so’s birthday so I'm gonna go out and then I'll have a couple of days indoors and then like hopefully I've not got it and so, it sounds, like I'm now so prioritizing social stuff, but for my own like, wellbeing whereas like I say before it was, there’s danger outside the house so stay indoors (David)  I feel like I could take more steps to being outside and sort of, getting involved in more activities and things if that makes sense (David)  Um, yeah we were, we were a bit more open to going out I think once or twice, we, we did go for maybe more than that but a few times when we did go out for a meal, previously, we didn't really, erm at the beginning we still stuck to sitting outside whenever possible but like once or twice, it wasn’t possible so we just sat inside and it was, it was okay. So that's something that we, and I remember it was in August, 2021, when we first went inside a place and sat down for a meal and it was super weird. But because of the vaccine, we thought it was, it was acceptable, and we wouldn't have done that without, you know both of us being vaccinated (Francesca)  Relief. Yeah, relief to have got it [vaccination] done. But It hasn’t changed my behavior, it hasn't, you know, even after the second one even after the booster my behavior hasn't changed. I'm still staying at home as much as possible, wearing what I'm wearing better masks now, you know, I'm still not going anywhere. Not, again not good going out for meals or doing anything else like that (Olivia)  You know [my daughter’s] had both jabs, [my other daughter] has, erm most people who I mix with will have the same jabs but it's like when we pop around to our friends for drinks maybe at the weekend, We won't go in their house will go in their garden. So I suppose socially you change the way things are (Daisy)  Ermm, I suppose I do actually [think about people’s vaccination status]. There was err, it was when [my neighbour] had his little Beer Festival, it was two years ago, and I was the only person who was inside everyone else was outside and close. So I suppose I'm not afraid to say, oh you know, I'll sort of stand away from this. Ermm, which I guess in some ways, you just have to be.Erm, but yeah. Not easy. I guess because im quite cuddly and very affectionate, that you sort of stop all that, which is, which is really really difficult [Daisy]  And the world seem to be getting back to normal and I wasn't ready for that yet. erm so, I found it difficult anyway so I got my second dose, because they brought it forward didn't they, so I thought all right I'm going to live so we did actually take my niece to Butlins. Because we thought, I, I knew it, we knew what we could do we knew that they had strict measures in place. And that was really, that was really good for me, it got me out, it made me go out and do things (Shauna)  Erm once you start learning if you’ve got it, it's okay, then you start socializing a little bit more and, yeah, it's kind of like disappeared, the kind of stress of people with asthma, even though it was lung related, but a lot more easier to socialize, erm after a few weeks but at the start it was still limiting contact and doing things outside (Robin)  erm still carried on wearing masks, like straight after. Erm I think I just use it as like an extra sort of thing that could say I that protected me against covid. Yeah it was quite positive, erm I mean the vaccine made me have like a little bit like of like flu like symptoms. But I thought, yeah just extra protection, I think, and confidence but yet still carried on wearing the mask (Robin)  And you know, I do things since I got vaccinated i'll go into shops a little bit more and that kind of stuff. I don't worry about shops as much now, I still wear my mask. Other people don’t wear theirs I’m still wearing mine I still avoid people. I'm like don't get near me thank you very much, and I pick really quiet times. I, you know I’ll nip over after the school run before anyone else gets in there. Erm I wouldn’t go over on a Saturday lunchtime, for instance (Sophie)  Oh, it's been a long time ago now did, did I meet more people? Did I go outdoors more? maybe I did in terms of going to restaurants and things like that, you did feel well, a bit more protected erm for a while, erm. I felt more relaxed about having my daughter in nursery so. that change has come simultaneous to covid so it's a bit of a case of how much of that’s related to covid how much of it’s related to my wee one. So erm, yeah, but I think that it was a feeling of relaxation a bit in terms of I had like some sort of defence system against it (Stephen)  So my children went back to school, my husband was back at work, I was still at home. So but by that that point, because I was shielding I’d had my first two jabs. So I had my first one in February and my second one that April. So, by then going back to school seemed less of a worry. (Annie)  Yeah. Yeah, i felt safer because the thought was you hopefully wouldn’t be hospitalised but we certainly were still pretty cautious, in what we did. I mean we still get home deliveries from Asda, that was we never cancelled that (Annie) |

Table S3

Theme 2: Asthma background to foreground “it’s only mild and moderate until it’s not”

|  | Pre-Covid | S1 initial breakdown / lockdown | S2 Some restrictions lifting | S3 All restrictions lifting/ Freedom day | S4 Vaccine and other occasions |
| --- | --- | --- | --- | --- | --- |
| Asthma background to foreground “it’s only mild and moderate until it’s not” | Before the pandemic erm I hadn’t had an asthma attack for some time which is, kind of plodding along I knew what to do if I got unwell, avoided situations where there’d be things like cigarette smoke or whatever that would set it off (Stephen)  So I would have said as much as I had asthma, it never really seemed to bother me that much, I had hay fever and things but I would manage it by taking antihistamines and things like that maybe increased my blue inhaler.... So up until the lockdown I had no problem knowing I had asthma in my life, because I barely would have, I would have left the house without an inhaler and that probably wasn't a good strategy, but I was well enough to not need it (Annie)  My asthma nurse said to me when I first got diagnosed (I only got diagnosed when I was 40) and she said that the aim is that you forget you've got it and you just live your life with it so well managed you don't remember you've got it. And you don't you know, so unless I happen to be out and come into contact with somebody who was smoking or something like that or a bonfire, bonfires are a nightmare, erm then I didn't even think I had it you know I didn't really, it wasn't front and centre of my life. It was just something I had and I got on my life with really (Sophie)  It is controllable and you know most of the time, not all of the time, and then it's in a pain the backside. You know, but when it is controlled I don't even have to think about it anymore... You know getting the diagnosis getting the medication, erm it didn't really bother me to be honest. It wasn't something I really thought about on a day to day basis. You know, you get into a routine with your meds, you just get up and take your puffs, you know. I don't think about it to be honest so it was never, it just wasn’t something I really thought about a lot of time (Sophie)  So yeah, it's very manageable and I've been dealing with that for since I was like, 14 So, yeah, going out, erm when like at uni and stuff I’d always have my inhaler in my bag, it was just the thing that you're doing, like as you’re putting on deodorant you’d just take your inhaler out. (Robin)  Probably a little more [think about asthma] than I had done a few years previous [after a hospitalising asthma attack]. I was aware and like I say, I'd never go anywhere without the blue, I always have new blue inhaler I’d And I was quite religious taking my medication. But yeah I was aware, I did know that if I didn’t take it it would get bad. So it was, I was quite very aware of it (Shauna)  No, I was diagnosed, many moons ago.  And you just learn to live with it. (Daisy)  I'm not sure I would say that just because, you know, when I noticed that The only thing I really have to remind myself of us to take my medicine. But otherwise, yeah, I rarely think about it and therefore it rarely impacts me or rarely have any symptoms and erm, and I think therefore, I don't really spend a lot of time educating myself about it because what I'm doing seems to work, and I'm happy with it.  (Francesca)  Yeah, I think so. I mean when I first. When I was first diagnosed with it. Is that, what that must have been half a year before the pandemic. I was, I was just frustrated that I have it now, but then as soon as I started taking the, the preventer inhaler and it was just, it just went away. I rarely ever think about it and the inhalers just become part of my brushing my teeth routine and it's just it's just something I do and I often forget that this is medicine because without it, it might be worse. (Francesca)  I don't think about it on a daily basis. The only thing I really have to remind myself of us to take my medicine.  (Francesca)  I'm not worrying about having an asthma attack, and you know, I've only recently put my blue inhaler back in my handbag but I didn't have it in there for two years because I just, I just really don't need it. So in that sense, I'm, I was pretty neutral about it but sometimes when I think about the long term impact it might have and what if it gets worse and kind of things that, that's that's frustrating and worrying but, yeah, not so much on a daily basis (Francesca)  so it [asthma] was almost something in the background that I didn’t really notice as such (Helen)  I mean I've lived with asthma since I was six and it was one of those things I can't just, it was always there and it just didn't even cross my mind that it was, that it could be better. I had a few exacerbations but I don't tend to get colds, which really helps ... And so I've been quite lucky in that respect in terms of chest infections and stuff. I kind of just got on with it. (Hadley)  I used to think about it constantly before I started to manage it and realize what was going on. Erm it was really frightening and would happen all the time and I didn’t really understand what was triggering it. And then once they'd got control of it, it didn't used to bother me at all. It was just annoying that I knew that I'd get it if I went running or something that it would limit how much activity I could do. But that's about the extent of it. (Molly)  I always relied on my blue inhaler as the kind of, you know, the kind of get out of jail card, really, erm which was probably quite stupid as well. And just, possibly just winged it for a long long time you know I’d had asthma attacks in the past but they kind of came infrequently. Erm you know I have been hospitalized in my adult life, but again that was, you know, fairly infrequently so I just kind of kept on with my inhalers and not really worried about it too much, you know, it was just one of them things (Chris)  before the pandemic [thinking about asthma], not particularly no, a lot more after the pandemic or during the pandemic (Amy)  I had usual childhood asthma, erm but then I've developed autoimmune asthma … in 2019, before so it was just like the pre pandemic. I'd not really taken my inhalers properly. I wasn't really taking it very seriously. And it was just one day out of the blue, that I woke up, and I couldn't breathe. And so ended up in hospital and intensive care, had a cardiac arrest… erm so, I’d become a lot more conscious about my asthma (Matt)  And you always think these things [autoimmune asthma; leading to cardiac arrest and coma] never happen to you, they’re never really severe they’re never you know, it’s just you being a bit you know of a hypochondriac really when you read about it and think you know that must be me. But it was that experience that really shaped the fact that, you know, actually I am vulnerable. And, you know, I do have very very severe asthma and, and it was it was a massive wake up call, really. (Matt)  Yeah, it was, it was constantly on my mind, you know, I was, I was just waiting for the next time that I’d wake up and I won't be able to breathe erm, and that time I might not be as lucky. So, I was really, really quite terrified to be honest. erm (Matt)  Yeah I felt okay about it [asthma] was just, go on my nerves sometimes but that's just asthma isn’t it. (Millie)  And this is one of the things that annoys me so much when you define it as oh it's mild or moderate or severe, well it's only mild or moderate until its not (Julia)  erm I knew it was something as I say I was diagnosed when I was two, so something I've always lived with. And I know I have to be careful in certain situations, yes I do have to remember to take my inhalers, and it's in the back of my mind is that it can be very dangerous. But most of the time I just get on with it, it's just part of my experience. So, I think before covid, something I was aware of was was sort of there in the background (Julia)  I mean I had much more problems when I was younger with it, where I had, and because I've always had asthma, I was just used to it. (Joanna)  Erm not a whole lot [thought about asthma], except for remembering to get my flu shot, erm be worried if I had forgotten to take my rescue inhaler. So, most of the time it's not a big deal, but it, there are just little things here and there, that, that, I think, Oh, I have to make sure I've done that (Susan) | So I suppose it wasn't as scary as it may have been. But once I got the call to say I officially was shielding of course your opinion then changes slightly at that point because you go oh, so I could die if I got this. It’s you going, I could genuinely get this and die so I suppose. We know more now And I think anyone could die and I think it's not always those with asthma. But I’m also sick of hearing in the news, asthmatics didn't die. For one because we’re all sensible enough to hide from it. (Annie)  I was really worried as I say of getting ill, of being disabled, of dying or that sort of thing. So I just like, it was very much a case of, If I get Covid I will be in a real trouble here. Yeah, it was pretty negative. (Stephen)  No, I mean at first yeah, I mean it was in the back of my mind, I kept thinking, you know, I hope I'm not one of these people that will end up on a, on a respirator, and I had, you know, there were instances where that I that was on my mind and I'd be extra careful. erm so yeah, it's a it was definitely on my mind. (Molly)  Yeah definitely it never would have occurred to me from a medical point of view that I was in danger of anything with my asthma, and erm it wasn't until the pandemic that I realized that that was puts me in a vulnerable category. erm yeah. I think the fact that I hadn't had it really badly for a long time like if I'd been in the situation that I was six years ago I'd be, I'd have been absolutely terrified. So I think that some part of me thought that it was all behind me, and you know I didn't have to worry about it anymore but obviously I knew that it was a possibility that I had some, some sort of vulnerability but yeah. (Molly)  Erm I think it was, I’m sure read somewhere it was to do with which inhalers you had. Like the preventer ones and which ones you had, like I’m sure the one I had it was classed as severe. Which I was shocked at as well, cos I didn’t think that mine was severe (Louise)  I almost felt like it changed because I felt like I'd always grown up just like I’ve got asthma, it was always quite like just an average thing and then I guess it was like being classed as severe asthma and then it was like you need to shield, it was a bit like, it changed it did change my diagnosis in my head. (Louise)  Really stressful erm I felt like it was almost a category of everybody high risk, erm and it was those obviously with different medical conditions that seemed like life threatening that were kind of treated erm sort of, a lot differently, erm sort of those who had to shield and. erm I'm very healthy apart from having asthma so it was a bit of a shock to realize that I am high risk like this could obviously attack the lungs or this could, it was quite scary, but very stressful, especially in a work environment as well. (Robin)  I remember at the start of the pandemic I really, sometimes I just didn't think I was going to survive (Daisy)  But then I think when you see the words on the paper you know you're in the most vulnerable category we're recommending you shield, that's when it really hits home (Daisy)  So I remember that I didn't, erm really thought about myself differently from the general population, because I have asthma also because I considered my asthma really mild, you know very well controlled, I never really think about it. Erm, I think maybe there's one reason why it was more present, like, why it was sometimes, I sometimes worried about it was because my partner worried about it a lot, like is he considered myself having moderate to like medium asthma. And therefore he was like, he was, he was very worried and he was maybe more, like, he tried to protect us more and also he cut back on his contacts and going out and, erm generally we're, we’re both academics and so we, we really, we read up on the science and we follow the guidance and were just very cautious. But yeah, at no point did I consider myself, well, I didn't really consider myself more at risk at the time, like I kind of knew about it but it like it didn't really hit. If that makes sense (Francesca)  It was occasionally, but it wasn't like, I wasn't super anxious or, I wasn't more anxious because I was asthmatic, I think I was, you know, just as anxious as everybody else was because we didn't really know what was going on and how to manage it, how long it was going to last and erm, how dangerous it was going to be and all these kind of things but not specifically thinking all if I get it, then I'm done. (Francesca)  I thought well I'm more vulnerable here I'm you know I'm, you know, I’m kind of targeted. You know, because you know people were saying I wouldn't get and it might just be a mild thing people but obviously if you have asthma or, you know, certain conditions and I suppose asthma was one of them as I remember, then you know you've got to be very very careful. So I remember about that time thinking Christ I could die of this if I got this it could finish me off. So that was always in the back of my mind (Chris)  It was… It was just strain, a real strain, because I didn't want to become hyper sensitive about it. You know, I didn't want to become, you know, kind of housebound. But at the same time I thought well hang on, I've got to take this seriously. You know, I can't, I can't mess about with this because we know so little about it. Erm so yeah it did affect, I mean I suffer from anxiety and depression anyway. And it just exasperated that really. At first it was a relief lockdown came in because there was no pressure to go anywhere no pressure to see anybody (Chris)  Yeah, it was. It was worrying, erm because I knew that the last virus I got had landed me in hospital on oxygen. I was quite aware of potentially, how it could end up, erm but at that, I was pregnant then, so I didn't know if the pregnancy had maybe impacted it more than anything else which it might have done (Amy)  I felt okay about it at that point, cos it was still like pretty controlled and I wasn't doing much that would make it worse at the time. (Millie)  It's scary, because I know how, like reading all the stuff about it, I knew how it would affect people with asthma more, because of the, because of your lungs and, it just worried me that I was going to get it cos I've got family members that are vulnerable as well. And I got worried and I didn’t wanna give it to them (Millie)  I was worried, especially after that attack. I mean I got more and more worried despite what the allergy specialist said, the more I heard about what covid did to people. And I thought, respiratory virus, with asthma. No, not a happy combination. Erm and then when I saw that, It's only severe asthma, which is a, an odd diagnosis anyway because there's no one definition of it, was only on official shielding list I thought this is insanity, erm asthma again is not being taken seriously. And then after the asthma attack erm at Easter. I thought, you know this really is quite a, quite an alarming thing to have, but made me more determined to not go anywhere near anyone, you know that might have covid. (Julia)  Quite a lot more than I would like to [thinking about asthma], because I've been told you have to do your peak flow every, twice a day every day now, asthmatics have to. Okay, I've got to do this, which then means I've got to, I have to think about it all the time I'm not living a normal lifestyle. I'm not even going out. I’m having to stay away from everyone, not just in lockdown that I can’t even pass somebody in the street. It was terrible. It was like, I'm thinking about it all the time and I don't want to I want to be able to forget it. Not that I’m thinking about it all the time but I’m thinking about it a lot (Joanna)  Worries, but it’s worries for the sort of what's going to happen when lockdown ends. Even if I’m safe at that moment, it’s not going to go on, it's things like wiping, wiping groceries, should you quarantine your post. Well I didn’t quarantine my post but you know. It’s the sort of mentality, and I know, I'm not high risk, but it's being, slightly vulnerable, it's being in that weird sort of in between situation. you're not completely vulnerable but you’re not normal either (Joanna)  Um, I wasn't sure whether it really put me at that much more risk because it is well controlled. Erm and I've always thought of myself as having moderate, but not severe asthma. Erm when I'm affected, If I'm affected badly, but the vast majority of the time I'm fine. Erm I was a little worried at first, but no more worried than I think anyone in their 50s would be. So, yeah, I think, the way it manifested for me was in terms of just be more cautious and being more appreciative of the lockdown rules, than, I think, other people may have been (Joanna)  I was very nervous. So just purely thinking well my asthma’s was not that bad but using that analogy that I gave you that if a chest infection can wipe me out for three weeks, I'm really nervous about catching this… I was probably very much thinking, I don't fancy it, because my asthma, if being honest, I'm quite a big a lad, so I'm sort of like that was against me so I was kind of very very much thinking, this isn't, yeah, this isn't a good time sort of well yeah (David)  Yeah, I think for me, like the mental side of, there's a, there's, is it the virus that's going to affect your lungs I’m a bit like woah yeah I do not wanna be getting that as an asthmatic, but I have been super cautious. (David) | I was worried about erm. Yeah because, actually I was because my asthma was kind of up and down because I hadn't got my medication right. And yes, I was worried about it actually, because I was thinking Jesus, you know, you know if I start going out if I get this then my asthma’s not under control. I don't even have that, you know, but then we both made the decision, look you know we're not going to go out, if we don't have to, you know. So erm luckily we're both in a position where we can do that. And that's just what we did. And then as far as my asthma, yeah I was worried about my asthma at the time but I thought well I’ll get that under control but I'm not being kind of, I’m not having to go to work every day amongst people that might have the virus, you know (Chris) | And that's what I was doing, I was protecting my kids as well I was thinking yes, okay, if I get it. It could be very very bad. But if kids get it could be very very bad too, you know. Erm, so, my friends didn't understand that to a certain degree because, you know, two of them specifically don't have kids, you know. Erm. So, life just carried on as normal for them. me, I said, You know, I just told them to be careful. Just be careful. You know, especially the guy who had asthma (Chris)  Very much so again [asthma on their mind] it’s like this leaves me so much more vulnerable than other people. (Stephen) | **Vaccine**  Yes. And actually, that's when I started to. I think it was around the time I got my second dose that I started to pay attention to the role of asthma in all of this, like I wasn't really before, up until that point, apart from reading, you know, people at risk probably have diabetes, asthma, or other chronic lung conditions, erm I didn't really think about asthma as a, as a factor but I got my, my first and second dose quite early on, I think in March and May, erm in 2021 ... that's when I that's when I realized that because of my asthma, I got given the shots early (Francesca)  Yeah. just remember hearing like the negative effects of how poorly it [the vaccine] made people and that worried me about getting it and then me getting poorly off it …Just for the protection, like for the protection of not getting covid and because I knew I was already at risk with my asthma, and I didn't want to risk for, like, heighten the risk… I knew my risk was still there of getting it [COVID] but it wasn't as much as what it would be if I didn't get the vaccine. (Millie)  Even with the injections part of me goes, well, if a bit of hay fever mixes with it, I think I’d end up at the hospital. So, you know, even though I've been jabbed and boosted I think it’s in my head that I’d probably end up in hospital (Annie)  I knew for a fact I was like, you know, I am waiting - I'd actually made the decision as soon as I’d closed that bedroom door er, in March, I'm not coming out until I’ve had a vaccine. I was like that, that's that that's my endpoint (Matt)  **Other – Contracting COVID-19**  I suppose, it was always on your mind again because I’d had it [COVID], part of me goes well, I was very lucky and I was classing that as and mild is obviously a very loose term certainly just now it’s a very fluid term, mild basically just means, now, not on a ventilator. And I think I was very lucky that I managed to manage at home and I wouldn't wish ‘mild’ on anyone given how mild felt to me and, as was I was just lucky that even just know my lung function tested really good, they’re saying it’s fine. they don’t have an issue with it so I'm just lucky that whatever has happened, I’ve been able to recover from it, survive from it. (Annie)  Some people said “I just want to go on and live my life” but I think you're still going “well that life could still be taken away at any point”. And it's not just about me being unwell I know it took me a long time after it [COVID-19] to feel much better, and that was mild. I know it took my, my friend who I possibly gave it to, my ex-neighbour, she ended up with heart palpitations after it so she had some problems as well. It also went from having no asthma into shielding asthma. And part me of me goes, if I got it again, I think I’d be hospitalised, I don't think I would not be hospitalized this time. (Annie)  The bad experience I had was the feeling of running out of breath when I was teaching. That was kind of a surprise because I hadn't had something like that happening, forever. And I, I just found it very alarming. I’d gotten very used to it, not being a problem. Then it was again. And also sort of realizing that I, you know, I had. I thought, okay I had covid, it's over. I’m double vaccinated I should be fine now But, yeah, but no there's still a little bit sort of clinging on (Susan)  **Other – long term**  Yeah, yeah, absolutely, I think if I hadn't, if I wasn't asthmatic, then I’d have still been careful don't get me wrong I you know I certainly wouldn't have been going about it, but I certainly wouldn't have been as worried about it (Chris)  I don't really feel like my asthma has made an impact on how I've experienced covid and the pandemic like there were a few moments where it was in the back of my mind and maybe if meet the [inaudible] like I changed I changed my behavior, a little bit just because I was aware of part because I have asthma, but no, overall I think, I think I just struggled with it in the same way that everybody else did. (Francesca)  I guess as time went on asthmatics kind of moved down the list of people that they were worried about and the kind of the guidance seemed to saying a bit more, that asthma was less of a worry, so I think, I probably relaxed a bit more with it. And then obviously you know by now it's it's now been what 3 years since I was in hospital. So it's been a lot longer. So it's less on my mind I suppose. (Amy) |

Table S4

Theme 3: Place in Society

BEING LEFT OUT IS ABOUT INCLUSION (E.G., ONLINE) EXCLUSION (EG SHIELDING; DEPRIORITISATION; FREEDOM DAY) FROM SOCIETY BY BOTH GOVN, FRIENDS, NEIGHBOURS

BEING LEFT BEHIND IS ABOUT HOW THEY FEEL WHEN PEOPLE MOVED ON AND THEY ARE STILL STUCK FROM RESTRICTIONS LIFTING ONWARDS

|  | S1 initial breakdown / lockdown | S2 Some restrictions lifting | S3 All restrictions lifting/ Freedom day | S4 Vaccine and other occasions |
| --- | --- | --- | --- | --- |
| Being left out | **Danger/fear vs safety/calm**  erm it did make me feel safer knowing that the people in my household erm was actually indoors and they wouldn't go out and come back come back with it. Erm but at the same time there is sort of the battle where I am obviously safe, but at the same time like I feel suffocated like you need fresh air, especially if you asthma as well. (Robin)  Erm and then when I saw that, it’s only severe asthma [that’s shielding], which is a, an odd diagnosis anyway because there's no one definition of it, was only on official shielding list I thought this is insanity, erm asthma again is not being taken seriously. And then after the asthma attack erm at Easter. I thought, you know this really is quite a, quite an alarming thing to have, but made me more determined to not go anywhere near anyone, you know that might have covid. (Julia)  And I think once we actually did shut down, and we could stay home after the first couple of weeks, where no one had really been near anyone, it was kind of like we're safe actually now for a little bit, you know, because we haven't seen anybody (Sophie)  I mean once we were locked down, I felt like, yeah, it was going to be absolutely, you know, as long as I was careful it would be fine. erm also, the students that I taught, they were pretty conscientious about things like masking and being careful. So, yeah (Susan)  It did feel like we were being looked after as a population. Not necessarily asthmatics but the population as a whole (Hadley)  The bar to be classed shielding from asthma was, initially, set, extremely high. You had to, if I remember rightly you had to have had multiple doses of steroids or hospital admission which didn't cover me, and I was quite scared by that, because I do have a poor immunity to erm viral infections, upper respiratory infections. So I I was very scared of it. Very nervous of other people's behaviour, erm. very concerned, not just of, not just a dying from it but of being left permanently disabled by it as well. I have had er, erm post-viral fatigue in the past that led to me, taking a year off of university. Erm. So it's, like it was very much a case of I know my body doesn't handle this stuff well and that the post viral fatigue was when I was 21, I’m a lot older and more decayed now so and I’ve got the little one to think of and my my wife to look after … (Stephen)  I remember the first time we went. Ermm It was before, it was before masks, but I still ended up wearing this scarf around my face but I remember feeling quite scared even to go out. (Daisy)  The woman next door just kept having loads of friends around. And, and that just made me so nervous. So it was almost like, I almost didn't want to sit in the garden anymore. (Daisy)  I was just so, so scared, that last trip to the supermarket, with people wandering about some people were clearly doing what I was doing, just trying to stop, stop for bits as quickly as possible. But it was before the real panic buying set in. erm and people were paying no attention there was no distancing. And I thought, I've written know I'm a medieval historian I know what plagues can do to populations. erm I was really, I'm really scared. Yeah, when we locked down, I actually thought, okay, it's going to be very bad for a while, but at least you're doing what needs to be done with a contagious disease, you're stopping the contagion, you’re flipping the contagion (Julia)  I know from when they treated me 2019, they really pulled out all the stops, you know there were a lot of times where they thought about just stopping and removing treatment erm but they still decided to continue. I knew that it will be less likely for them to really persevere, if I was, if I was to be able to be ill during the peak of the pandemic. So erm, it was quite terrifying in the sense of, you know, I knew that if I left that bedroom. And I caught it, that was it. (Matt)  I had an extra inhaler and I used that up but then I left it and had lots of asthma symptoms, and I didn't get medical help for it, because I was too scared they'd make me go to the doctors but actually I phoned, and I spoke to a lovely nurse, and we did it all over the phone, and then she sent the prescription through for me. And you know my husband, just nipped over and got it for me. But it put me off, and I think I left that flare up, and it took longer to clear up, because I left it too long. You know, because I didn't want to go to the doctor's, the thought of it was just, no way I can't do that. (Sophie)  But erm it wasn't, it wasn't drastic but then I was even if I'd have had an asthma attack, I wouldn't have gone to the hospital anyways because I'm scared of medication anyway so going in the middle of a covid epidemic isn't gonna work so I just kind of rode it out and just took a lot of the blue inhaler. (Shauna)  It's just so difficult to get an appointment with them and it was all becoming so I know I could have probably pushed it but I just felt like, I don't want to go into the doctors and it wasn't, It was not easy to access the facilities to ask for something different because then I would have to go into the doctor's surgery and all of this. And I just thought, it's not worth it. So, I just didn’t, took the risk really which was a bit stupid really thinking about it. (Molly)  yeah I mean, I mean partly it, I just didn't, I didn't want to engage the health services at that point, it was fear of them, of catching it there, or, you know, just being so difficult. I just couldn't get hold of my practice, it was just seemed impossible to make an appointment erm, dunno, it just seemed easier to just manage it my manage the fear myself and just, You know, hope that I didn't get it which sounds fairly risky (Molly)  And, you know, and I was really really worried that, you know, something might set me off in my room with like dust, because I know that really my only big trigger is dust, so I was like just constantly cleaning everything all the time. Cos I just really didn't want to end up in A&E because I knew that A&E they would say that they would keep me safe from, from coronavirus but they, they wouldn't to the level that I had been isolating, so erm was really concerned about that, but but luckily I never actually did end up going into A&E at all during the pandemic (Matt)  **Anger/frustration**  And that was it. And it was over a month later, that I finally got told, you need to shield. And I was like well I’ve been doing it now so. So you know, but yeah I think those early days of the pandemic were really, yeah I was, I was quite angry. I felt quite like like I say invalidated, and I knew what had happened. (Matt)  So I knew what was happening. I knew. And you know, I could see that people were developing acute respiratory distress syndrome, which is what I’d had. And, you know, they were talking about, erm, I was I was actually very angry with the government at the start as well, because because they kept focusing on, oh we need ventilators we need ventilators, and I was like, ventilators aren’t going to do anything, you know, erm people's lungs will be so damaged, we’ll need ECMO. Erm and we only have like 15 ECMO circuits in the country. Er Luckily that surged up to about 90. But that's still 90 for the whole population. It's not enough. Erm so I was really angry and, you know, they were saying you know we’re learning and all this, and you know, we now need to give them steroids and I'm like, you gave me steroids last year, like it's not a new thing. And you know, oh ‘we've learned how to prone people’, I was proned as well, you know, these were all things that were being delivered as, oh we're making, we're making strides we’re making progress. We’re not, you know. And that was a very frustrating thing, it was, it felt very very invalidated about my own experience. Erm and I also knew that I was very very vulnerable to it. Very vulnerable. And erm, you know, it was, it was what April, that I finally got a letter which said you are clinically extremely vulnerable. And I was a bit like, I already knew that (Matt)  yeah I can, I think its, also being that careful, I feel like as an asthmatic I’m being penalized for being careful. I’m careful with my asthma. I feel like, I’m lucky because my GPs so far been on my side but, I feel generally asthmatics who are careful are penalised for being good at self management. (Joanna)  **Stress vs relief**  You know, I can't, I can't mess about with this because we know so little about it. Erm so yeah it did affect, I mean I suffer from anxiety and depression anyway. And it just exasperated that really. At first it was a relief lockdown came in because there was no pressure to go anywhere no pressure to see anybody (Chris)  I know I was relieved that I wouldn't have, I wouldn't have to be out there with people mixing and, you know, and that kind of thing (Olivia)  Relieved actually [when lockdown announced]. In a way I was actually relieved, I was like, shut the front door. and I'm like we’re at home, it’s like not rushing around like headless chickens not, you can just be, it was actually in a way it was a relief… I mean okay I was I was still very, I think I was, very anxious before that, I was still very anxious in the first two weeks of lockdown. But it was gradually getting less. I was just like, in terms of being anxious and not being safe, I got much better after the two weeks into the lockdown. (Joanna)  **Loneliness vs companionship**  It was like all of a sudden your life became very very insular. You know, all I can say is thank God for the cats. (Daisy)  I'd say my mental health has suffered worse, because when we were in lockdown. There was very little chance of me catching it because it wasn't going anywhere. I wasn't going near my husband. And he’s the, there’s only us two in the house. Yeah, no, just yeah numb. Just numb. and then that in itself is a mental health issue, just to stop feeling. But yeah, physically, physically and mentally, numb. (Shauna)  My husband had to go out he was an essential worker. erm so he went in and out, erm and he would come in, strip at the door leave his clothes for the washing machine by the door erm shower and everything before, before I came anywhere near him, and for the first part, probably about three months maybe he, I didn't go near him. We stayed metres apart and we slept in two separate bedrooms. (Shauna)  Yeah, and I would watch the same episode of friends, at the same time so then we’d ring each other afterwards and talk about it. And we had our little, little friends quizzes and Harry Potter quizzes, and it was that, yeah it was little things like that that got me through. (Shauna)  Um, I tried to distract myself, like I did seek out more like virtual contact erm. I called friends that I haven't called in years and that kind of stuff. Erm, and otherwise just tried to find a good book or a good series that I could happily, you know, spend hours in a day just distracting myself. (Francesca)  I'm quite comfortable, sort of in my own company and stuff. So, like first couple of weeks was like ah great and I guess if you remember it was really warm weather wasn't it. so it was sort of like, we're going to be on our own for a couple of weeks, you know, I’ll get, get on top of the garden go out for a run, sort of quite content, and just like, it's like a mini challenge, I think, after that, it then started to dawn that it's going to be a while and, you know, like, like, I'd stopped playing football stopped going to the gym, stopped socialising and then it suddenly sort of caught up with me (David)  Yeah, I think the reality hit of kind of like, oh this could be a while … then I think I struggled with the lack of social cuz I live on my own. I think if you’re in a lockdown with your partner, or partner and kids, at least you’ve got that conversation, and so I did probably struggle with that, if I'm honest (David)  Yeah, it wasn't a huge problem, because we were so busy anyway, with having the kids at home full time. So we wouldn't have, have the energy to do anything anyway! I mean we had a lot of zoom calls with friends and things anyway, so yeah, didn't find it, a massive issue, not seeing people (Amy)  I'd already experienced what it was like erm to not be able to see people in person. And, and it was actually harder at that point [when been hospitalised pre-COVID] to get people to, you know, ring me and speak on FaceTime because they were all busy like doing that, like, yeah, with their lives. But then when the pandemic hit and everybody had to go into a lockdown, everybody was doing it remotely. So actually I found interestingly my social life improved a lot. Because it was, it was just you know everybody else was bored sat at home so they were like, you know that let's give him like a ring. So yeah, I felt a lot more included at that point, a lot more included. (Matt)  I don't know like, just, you couldn't do like simple things like going out for, just to do the shopping, you were limited in what you could do and, me I love getting out and about, and you couldn't do that really could you, you were very very limited on what you could actually do. (Millie)  So it was really hard with that those sort of family things that you just take for granted, really. And yeah, they were times in the lockdown where I just wanted nothing more than to just walk out house and go for a coffee with a friend. It’s like I love you family, but I just don't want to see you anymore! it's just like, oh, God, you people are getting on my nerves now! That was yeah, that was yeah, it was hard not seeing people I've got to say (Sophie)  erm whilst in the lockdown erm I did a lot of online shopping. my parents did a lot for me as well. Erm but it was just really limiting contacts and again it can be quite lonely as well. (Robin)  Oh. Well it was actually quite positive in some respects in terms of not having to commute to go to work like two hours, up to two hours each way erm you know and actually getting a bit more outside time, but that that so that was really positive kind of from a mental health perspective but it was just like that that fear, anxiety, of the unknown was really concerning I missed out on social connections just like having conversations and I still miss that know, colleagues, friends. (Hadley) | **Danger/fear vs safety/calm**  I remember feeling safer because the thing that always worried me the most was being on the bus going to campus, because that's when you sort of encounter the most people and they're the closest and the ventilation isn't great. And at that point, because everyone was being really respectful about sitting far apart, wearing masks. That felt much better. (Joanna)  So I’m like again in my head, Dooms Day thinking, I’m thinking yeah if there's 30 people in that GP surgery one might have it, well sod’s law, I might get it. And I haven’t got a vaccine, so it's gonna, it's going to topple me over … so it sounds bad but I was kind of like, do I really need to come in, to discuss the maintenance of my asthma that I've had for 39 years... I really didn't want to take the risk it sounds bad, I was quite happy to just say let's just ring me on the phone or on WhatsApp. And I'll tell you it's fine. but erm yeah, yeah so probably yeah very cautious, I don't know, like say I think the lack, the lack of sleep, and then like I say having asthma, probably wasn't a good combination sort of thing. (David)  Yeah that's right yeah and thinking back then you just reminded me it was. I mean, where I walk my dogs, I walk by a canal at one stage, and there were people who were running by and just coming past and you know breathing fast, and it was like back away from me now, you know nobody knew how, you know, how you could get it, you know, how you know close people had to be to you or you know whether there was a kind of a viral cloud behind people running, you know, erm so that's possibly why I didn't start running outdoors, you know, to the best of my, from what I can remember that, you know (Chris)  You know and I do still struggle with that especially around the social distancing, and you know people not being too close to you so that's still quite a thing for me. And I still now, that when I was off to the gym on Sunday when I was off swimming down the road, I still sort of crossed over the road to avoid. So I don't know if that's ever going to change actually (Daisy)  It scared me, I wanted to do it so much, cos I wanted to see like my friends and my family and but it did frighten me about getting it and catching the virus and it was like with work as well. cos we like switch, cos with the job I’m in, there are like handovers and a switch for people so there were people coming in and out, so it just worried me if they like were to bring it in.  (Millie)  erm I didn't worry about it from a risk standpoint that didn't bother me at all, because I'd been vaccinated, and I didn't see, I think I'd become extremely cocky if I'm honest, I'd started like, to me, because I hadn't caught it yet, I just couldn't believe I hadn't, it had gone on for so long that I thought if anybody's going to have got it it would have been me and I haven't so I just thought, now that I haven’t got it, I don't feel the risk is there anymore, so I wasn't being particularly careful. But it certainly made me kind of more socially anxious, which I wasn't really expecting but apart from that, I didn’t see a risk in my health anyway yeah (Molly)  Well, I was worried, but it was sort of a, it was a background worry. It was a, I can't forget it. I can’t forget it. But I wasn't. It was just… strange. I mean, I can’t really explain it. But, at the time though I felt, well, we know that there will be a vaccine at some point, and it might be coming sooner than we, soon we hope. So that was quite comforting. And then sort of just hoping, hoping to count as vulnerable and get your, getting it sooner rather than later. Hoping (Joanna)  we were very very cautious and we thought that the lockdown restrictions were being eased far too quickly. Um, it just seemed that, you know, whilst we didn't really know anything about what was going on we just seem to be going poorly, it was just, you knew that they were just trying to edge edge edge all the time and not give people a chance to kind of stay safe, you know, it was just a feeling that there was a pushing all the time to kind of ease restrictions ASAP. Erm, and people's health was kind of left on the sidelines you know that's how I felt I just felt extremely nervous about it you know. but erm thinking back, it was. I was probably right to feel nervous actually (Chris)  I remember queuing at the butchers for meat - Ah, and everyone in the world decided to sort of come up to you and touch you and it's like, you don’t realize. And then there was a woman who used to work in the co-op completely denying, there was anything there was anything wrong. So I just felt as though every time I left the house, you'd have a constant battle with someone like, I work with the NHS, this is real. and they’re standing there with no masks on. (Daisy)  **Anger/frustration**  I guess it was just. It was just the concern that if I did get it, how you know how I would be with it, and kind of the awareness that I could potentially, you know, when you see all that the comments about pre-existing conditions, and it kind of, the media was kind of giving the impression that the people that had existing conditions were gonna die anyway. and whereas you know for me i'm 35 i've got 2 young kids as far as i'm concerned asthma isn't going to impact on my life expectancy at all or not significantly, but but knowing that actually, if I did die, I would just be classed as having some, having a preexisting condition, and you know it’s not, not that important really. So that that was sort of quite, not not nice to read that in the news, so so regularly (Amy)  I was angry because I didn't think it was appropriate either erm, you know I was quite angry, that the government had opened up so quickly. Erm, and I knew that what was going to happen was cases were gonna spike. erm whether it was in a week or twos time, whether that was in a couple of months time. I don't know but I knew for a fact that they would come up again. And because it just makes sense, you know the way that viruses, get transmitted so I was just waiting for it. I was waiting for it, and you know everybody was saying to me, you’re being so pessimistic, blah blah blah. I'm like, I'm not being pessimistic I'm just being realistic. (Matt)  **Stress vs relief**  I think it was was it like March April May, was super into fitness, and then I think like I say the mental health then took over. And it was a bit like, just struggling to see the end of it, it sounded bad but it's a bit like, it's like a long stressful situation, you know, like if you’ve got a month of revising for a big exam but then at least it's over, you just like, it's never ending. (David)  **Loneliness vs companionship**  erm good to be honest, erm having things open obviously good for the mental health, you know getting to see friends again erm I felt like my asthma was under better control erm and yeah I felt a bit more at ease with it you know covid was a bit more familiar, so I felt happy going out and seeing my friends and what have you (Helen)  erm yeah I would say so to be honest, I think after all that time away from friends and what have you its nice to get back together when we could, so erm yeah I definitely took full advantage (Helen)  erm not not massively I don't think, it was hard obviously you know seeing when the kids are missing their friends. Erm, that that was kind of the worst bit, I guess, and like not being able to have birthday parties and things like that. I think you kind of felt more for them, because it was harder for them to adapt to it and cope with it, I think, than it was for us. erm, yeah (Amy)  they they came up for a weekend and we met, I found somewhere that was so remote I knew they wouldn't, there wouldn't be many other people around. And we met and we had a picnic. And then we both put masks on, and we hugged and it hit me far more than I realized because I haven't, apart from having my vaccinations, I haven't touched another human being, since early March, previous year (Julia) | **Danger/fear vs safety/calm**  It’s like look, this hasn't gone away and suddenly we’re all free to mingle so it was sort of quite scary. But there's the recognition that well this is just how it's actually gonna be now, they're not gonna go back to restrictions and lockdowns, because that was the point really when the government started to basically ignore the science in my view because it wasn't convenient for them anymore. Erm. So. So that was that was quite scary really as well. We hadn’t really altered much as to what we were doing except that at that point my daughter kind of started nursery as well. There was a limit to what we could provide for her at home at that point (Stephen)  But that was in June, and then in July when freedom day really opened up and everything, you know, people just stopped, it was over the pandemic. Like no the pandemic is not over. erm so I felt in more danger at that point, than I had done a couple of months before, and I was like you know what, what what's going to happen now is that just gonna be a variant, because that's what, that's what viruses do. Erm and because it's spreading through a partially vaccinated population, it's going to evade immunity… I felt more limited once freedom day happened, that's when I started to feel more anxious, and I actually started to invest in FFP3 three masks erm whenever I went out. erm so, they’re really, really expensive, but I just thought, if it’s good enough, if it's if it's good enough for me to use down if I was working on a covid ICU, then that's good enough for me to go out the house. so I just yeah, absolutely always would wear a mask whenever I'm out the front door now. (Matt)  And so, what the rest of society was doing seemed bizarre to me I just couldn't comprehend erm why, why it was happening and why it was safe, erm I didn't feel safe enough, like, I thought they were going too far, I understand the reasons behind it and why politically and economically all of this has to happen. Erm, but from a safety perspective I didn't think we were there (Francesca)  You were back in a bit of control, I think in lockdown potentially a lot of people would be, you know, at work and would be forced to do things that would put you in danger. And that's why the completely removing of shielding or whatever, the fact that it doesn't even look like the conservatives are putting that back in, is a worry because there was protections in place to make sure that you could get a supermarket slot, that your work you know had to follow more stricter rules, more stricter guidance. They had to make sure that if you didn't feel safe that they would have to take it into account and I think there was quite a lot to protect you at that point and it was a choice, and I still think most people in a similar situation to myself, still make those choices. You know they have chosen to go, no I'm not gonna go to the cinema, no I’m not going to a concert I mean, I happened to win free tickets from sky TV to go and see Steps, I was like, No, of course I'm not going to see them, I thought, there's no way I'm going. And interestingly, two days before it came out, we got an email saying you do not require to show proof of a vaccine before you enter the concert (Annie)  She was coughing like a trooper sitting there. And I just thought, maybe you shouldn't have got on public transport. You know, it's very. I think people can be inherently very selfish. Erm and there was also a point where one of the service users, who's also a staff member, refuses to wear a mask and someone wanted me to manage them and I just said no. I said I'm not going to do that. And, and that was all a bit scary. (Daisy)  When freedom day came, that was a nightmare. I just felt lost. That's, that set me back. People weren’t wearing masks, people couldn't be bothered with social distancing erm it was just a bit all to them. And, yeah, that, even though I had my two vaccines, I wasn't, I didn't cope with that very well at all (Shauna)  And then I'm in a new job now and it happens up there where I am and it's just so like panicky that, because the kids had got it at work and then all the staff had got it as well. So it was like I wanted to go to work but then at the same time I didn’t cos I was panicking in case I got it (Millie)  It was terrifying. And er I'm sure you've heard this a lot. The so-called Freedom Day was lockdown day again for me, I'd start doing click and collect from the shop, where I’d go in and talk to them because everyone was wearing masks. I did a click and collect in mid-August, after all of the restrictions gone. And I, I was horrified because there were people in there that, even one of the people in the members of staff wasn't wearing the mask. Luckily I was wearing a mask and I was in and out quite quickly. But I said to them, I'm not coming back, if I do a click and collect again, I’m going to wait outside to get it, actually I did this the other day, I said I will phone you when I'm outside, and you can get somebody to bring it out to me. And it really meant that, and I also did a click and collect from pets at home, because I couldn't get something I wanted online. And again, I was just so terrified by the complete lack of any, er even basic common sense. We're still in the middle of a pandemic. I did, I was absolutely horrified and terrified. And it has been hugely detrimental for my mental health, and my feelings of security. Why couldn't we of just kept some of the basic mitigation measures? (Julia)  I mean not, getting rid of masks in shops, I wasn't comfortable with. And I’d say, and in fact I actually started, I started going to go for groceries, but also to the wholefoods shop. And I pretty near stopped going for whole foods, because I knew that nobody would wear a mask in there, or hardly anyone would wear a mask in there. Whereas in the groceries, most people will still wear masks. In the whole foods shop, I mean even the staff wouldn’t wear masks, at all, so I was like I don’t really feel comfortable going in, I still go in occasionally but only if it’s something I could not get anywhere else (Joanna)  It's strange because you feel that some people feel there's nothing, feel that it's all over, and it’s not. So I feel nervous that people feel that it’s all over and are not being cautious. And even more recently I went to, erm I went to a thing at local theatre in Hampton, because it was a concert, I took my son it was children with additional needs, a special needs concert. and I felt a bit nervous doing that even, but we all had to test beforehand, and anyone who could wear a mask did. There were a lot of genuinely mask exempt people there, and I was aware that that was going to be the case. So that did not feel too, I felt it’s a bit risky but at the same time it's probably okay, I’m not too worried for me. I think there's more other people there who are probably at a much greater risk than I am. So I didn't feel too worried about it. (Joanna)  Definitely not ready. erm I think it was a crazy decision to remove all the restrictions at once, like that. Erm It was a real concern (Olivia)  **Anger/frustration**  Um, it did, it affected it negatively I'd say because erm with certain friends it was the case of, will you come to the pub? Well I don't want to. Why? because there's a fucking pandemic going on. Oh well you know the restrictions have been eased blah blah, and I said well I don't want to, you know, simple, I've taken the choice not to, you know, and it was a case of well they didn't understand. You know, I've got a friend who has asthma like me all his life, and he was out to the pub and you know, you know, but he was, he was calling people erm hysterical. Oh they’re hysterical over here you know, it's ridiculous , you know, it’s only like a flu. And I was saying it's not like a flu they wouldn’t close the whole fucking economy down for fucking flu. (Chris)  annoyed. Whenever you sit on the bus with, you know, with somebody who's hacking with no mask and, sort of, you know, and then other people on the bus were all, getting up and moving further away. (Susan)  I sort of didn't like the idea of Freedom Day, either erm because it, you know, it's sort of, I guess it gets to the whole public health messaging around this. And to be fair, what I do, part of what I do is study health communication so I'm kind of biased. But it felt like when they said it was Freedom Day, it was all about, okay, we're all now free to do whatever we want, rather than thinking about, there are a lot of people who are not free. Who are still worried. and because of, you know, the vaccine was rolling out still, it hadn't, we still hadn't reached full coverage of the vaccine, and I felt like too many people sort of took the license to say, Oh well, it doesn't matter anymore (Susan)  I'm kind of infuriated because even though we have freedom day there were still people on the bus coughing and touching everything in sight, and not wearing masks. Erm even though I know a mask is not perfect, like you, you could at least try. (Susan)  actually there is one thing, you know, it was, it was basically that the government that we have, just wasn't trustworthy enough to be able to feel safe, I would say, you know. I didn't trust the government all along. You know, I still don’t trust them, obviously. But back then. I just I thought they were monstrous, what they were doing. I did. You know and it just it really, you know, my anxiety and depression because of that was was brought from, fear because of, you know, it was because we didn't have a government that had proper leadership, that could actually say look you know we're going to put people first. (Chris)  certainly back then, you know, 2020 summer, people were more understanding I certainly think winter 2020 a few of my friends and family were possibly less understanding and aware I think people were just sick of it, but I think a lot of people suddenly took the opinion of because of being vaccinated there was no risk, which is stupid. (Annie)    Freedom day was, freedom day was horrible. it was, it was really at that point where I felt like the government, the public, everybody was just giving the middle finger up to people who are vulnerable and just saying, your life does not matter. You know. And that’s what, that's what really upset me And it was going out when I'd have my mask on, and people would give me funny locks. And I was like yeah okay you know I’ll just walk down here and I went to a pub once, where we go outside, into the beer garden this guy stopped me was like, Oh, you've got erm, because, because I would wear a FFP3 three mask which is like associated with ICU. So I would wear another face covering over it just doesn't look as ominous. And he goes, oh you’ve got two masks on there don’t you think that’s a bit excessive? I just had to go like, I was I was on a ventilator two years ago, and he was like, oh. I was like, you just don't know, just, yeah. Because nobody would notice that walking past me on the street (Matt)  oh it's it's awful, well it’s the mantra isn't it, I don't know if I feel more annoyed at the government or the crap press who don’t seem to really do anything with the information they must have, you know, for these things because you do kind of go, why, I, I mean, even from Nichola Sturgeon the mantra is we must keep schools open at all costs. What, what’s she done about it? nothing, nothing, nothing’s happened. There's no extra mitigations, she didn't make teachers, a priority for boosters I mean. Fair enough, everyone should have them probably within the next two weeks but that wasn't, you know it's not as if she went teachers are first. Teachers go below healthcare. You know, none of the governments have done anything to make it safer for children and schools, high schools do have masks still up here that has been a mandate that’s not left. (Annie)  But my kids are still primary and they’re still at the age where they will share sweeties with each other, you know, they’re just young. They’re just gonna do it, It's not, it's not the best we also have, my children’s school’s an open plan school. So there's nothing stopping that going anywhere. there's no you know walls and doors between classrooms. So, there’s an added risk there, erm the school had done away with the staggered start and stop times. I had a fit at that because it's still in the government guidance with the head teacher’s response being it’s disruptive in an open plan school. However, she went, I know your situation and I’m happy to accommodate you to make sure you're not meeting everyone at the same time so she did, I picked the kids up 15 minutes early every day. Cos I said you're doubling my risk you went from half the school being packed up at the same time to the whole school being picked up so you're doubling my risk. (Annie)  The government you know it’s same with the council, the government, the government doesn't look good. It doesn’t look good if the people think the school's not safe. And they’re not, just, they're not safe. So the biggest concern for me has always been the kids in school, and also they have no jabs whereas eight countries around the world are jabbing, they’re given a choice there’s still a choice to jab them but the choice isn't here for us yet, even Ireland. Ireland’s giving 5-11s the choice  But I would rather the government come out and said, we don't have enough, and the priorities are older groups because they are more at risk than the children so that's why I would rather them be a bit honest, rather than, the children don’t get it. (Annie)  As you're saying to yourself, for those who have say, COPD, you'll die if you get this, that's why you should wear a mask. but you know it’s basically two camps now, it's always those that don’t want to know any more about it and those who are horrified and I, I still think I can't believe at the end of play yesterday it's still 183,000 cases in the UK and people are like okay, it's just mild and you’re like well about 3% of Delta were hospitalized, even say 1% of Omicron the hospital can't cope with another 1800 people getting added every day. And even in today’s press they’re going to open Nightingale’s hospital again, and you’re going, why are the press not jumping on that. Boris you said it was fine but you're opening hospitals here, come on, there's something going on here, there’s something. And yet the still no talk from any government about those at the highest risk. What do they do, are you going to give them a booster? A fourth booster? And when? are they going to tell us to shield because they can't give us a booster until they’ve given everyone else one? (Annie)  So, I booked, it was two weeks before I could get an appointment with him (GP) but by time I got to him I was actually feeling a good bit better. But that took from the middle of September to the start of December (Annie)  Yeah, I was probably thinking about it [Asthma] quite a bit and then thinking like how's it got worse, in the sense of, like, I do still seem to have symptoms as though I've got covid, which means I'm getting tests all the time. But yeah, and then I feel like it's, the doctors are quite dismissive of it because they keep just putting it down as long Covid it without really, they just keep saying you've got long covid, without really like investigating it further. erm it's frustrating really, and I don't know how like, how much to press that, like how much I can be like my asthma’s not managed. (Louise)  [masks not mandatory] … I mean I guess it's people's choices isn’t it but err, I suppose there’s a part of me that thinks that that is really irresponsible. And when I went to get a cup of tea I think I must have said this to the guy who was serving the teas like. He he’s like well it’s up to, to each and every one isn’t it. And I think I have to be really careful within the field I work in because a number of people who, if I chair meetings, I know that they won't have had any of their vaccinations because they think it’s some kind of conspiracy theory. (Daisy)  I felt it was irresponsible from the government, their science, it was contradictory to what WHO and other scientific bodies were saying, and the rest of the world. They'll wear a mask I mean I've been away overseas, and people are wearing masks in the street. Even in the summer, in hot conditions because that makes sense. I don't know why people, why the government's not taking such a precautionary approach, there are things, I understand you know they want to keep the economy going but then there are other things, that aren't going to hurt anyone. People are used to wearing masks, that was, I mean that was frustrating. I mean yeah, the government at the moment, I think they’ve lost respect of everybody. (Hadley)  I suppose anxious and Angry. Yeah, just a real mix of emotions. You know that that vulnerable, anxiety, but also, You know, take some responsibility. Erm but I think the thing is in those circumstances we can either be angry. Or we can do something about it so I choose to keep wearing the mask (Daisy)  **Stress vs relief**  The only I’d say, I'd say something I still wish they’d have done better was the fact they completely removed shielding and I understood they couldn't see everyone's got to hide away forever, but I certainly think all governments should have kept protections in workplaces for people. you know, if someone felt they couldn't go to work, they felt unsafe at work I feel all that, again didn’t affect me I think I’ve been very fortunate, but, if I'd been asked to go back to work I think I'd have really struggled with that mentally. I haven't, haven't cos it’s out of your control. You know, the restrictions ending was fine for me because I still had control over most things in my life. didn't have the control over the children going to school, but I kind knew at the time that I probably could’ve worked with the headteacher if I had something come up in my life. erm, but I definitely think that's the one thing that they got very wrong is the fact they still don't have any protections in the workplace for people that, if someone had said to you, you need to back to work tomorrow. I don’t know how I’d cope with that. I’d probably go off sick and it's not that I would want to go off sick, but I think I'd be very stressed by it, and especially knowing that I've managed to work at home for almost two years I think it would be harsh to say I couldn't continue to work at home. (Annie)  So, like you say the freedom day, I think luckily enough I’d had one jab, but if I hadn’t of got into that, I’d have been thinking, well I’m still not leaving the house (David)  I know you're saying it's fine. But, yeah, you haven't got my lungs which are pretty terrible sorta thing so it was kind of, I know there was a really stressful period for like 3 million asthmatics or something, and my mates were like, Oh yeah, you'll easy get, and cos he worked for the NHS, and he was like oh yeah you will definitely get it [vaccine], you will get yours in like March or something don't sweat it. And I had to like fight the GP write to is it the erm some clinical commissioners, just to get a jab (David)  it was a bit stressful sort of thinking, yeah, so there was this whole, like, so like someone I know who's asthma’s not as bad as mine and got theirs in March and they were like 21. And you’re like, well okay, but in Lincoln it's like no you're not allowed it, then in Manchester, they're just saying, anyone can turn up and it was just, I mean it felt like a bit of a lottery as to what was happening so yeah Freedom Day for me still came with a lot of caution. I was, I was, I was kind of like once I’ve got my two jabs in two weeks that, probably my anxiety levels will start to sort of think, you know, okay, we're back to a bit you know of norm. So yeah, I think, I'm thinking when I got mine but so yeah maybe like, May and then November or something so you're still pretty nervous (David)  So, it's just shocking I feel this, and people go on about oh this freedom day, what an insane thing to say. So it's, it's, it's curtailed my life. It's made me much more nervous and stressed but also I've stopped doing what I had started to do again. I've, I have seen my neighbour, but made sure that we're sitting a long way apart in the garden. And we went for a walk together and then when we came back again there's one point where it was quite narrow and of course a railway bridge so I put my mask, I said to her I'm going to put my mask on, So it's it's it's really it's been really detrimental. (Julia) | ***Vaccine***  **Danger/fear vs safety/calm**  I wouldn't have done it four months before so that was good, and we came out of it going, we all felt quite safe I think we would probably do that again. And then within what 3 weeks later omicron popped to tell you that you've got no immunity pretty much towards it, you know, but I had my boosters then as well. That was the end of October.. But, obviously, the worry is now they’ll tell you that lasts 10 weeks, my 10 weeks runs out on Saturday. That's 10 weeks since I had my jab this Saturday. And they’re saying the booster only lasts 10 weeks (Annie)  Honestly, and I think eventually, when I had all the Jabs, you start to feel. I don't feel 100% safe, not at all. And I'm very very cautious of things still. (Daisy)  Yeah. It definitely made me feel less, less concerned. and I think actually, that was probably part of being more relaxed about going out because I was sort of thinking well I've had the vaccine now. So you know, the likelihood of me being seriously ill is massively reduced now, erm. Yeah. (Amy)  Well, I was still nervous, but at the same time had a lot less nerves because we’d had the vaccine (Joanna)  but people are behaving as if it's all over, and then it’s like they’re saying, oh we’ve protected all the vulnerable people, well will they go on, will I go on being protected will I get when I get booster. (Joanna)  yes ive had the booster but I’m worry will I go on being able to have it. Now for me, what would help me mentally is to know either that everyone with asthma will be allowed to have the booster for free whenever they need do it. Or they come in with a paid for option like with the flu jab. That would almost set my mind at rest because then I know I'd have a backup. I mean, I don't, obviously I’d get it for free but, if not…a paid option like with the flu jab, yes I get it for free but if I can’t i know that I do have a backup. I know that there is a way of doing it (Joanna)  but then omicron hit, and everyone got them quick, and because I’m over 40 I actually was able to book mine for the 6 months, after but it was because of my age, not my asthma. It was because of my age and because of the omicron that they were rushing everyone through quite quickly. So I still have that worry, that what happens, you know, when the next lot are, you know will I get it? Might I get it, If they only do vulnerable people, and I still think, if I’m vulnerable to flu, surely I must be and I’m not a doctor, but if I’m vulnerable to flu, surely I’m vulnerable to covid (Sophie)  and my husband was like oh it’ll sort itself out it did the last couple of times. But it, you just don't know. I mean, I presume they're probably just gonna give it to everybody. and I guess at some point you'll be able to get it in the same way with the flu jab that if you don't get it free, you're probably going to pay for it. And that's fine, I don’t mind paying for if I have to. Erm, you know I’d pay for the flu jab if they wanted to me too. I get it free from the surgery, so I do it that way. But yeah, that's always there at the back of your mind, am I going to get the next one when I need it (Sophie)  It felt, it felt good to have had them, and I felt safer once I got them. I still don't feel safe, but that summer holiday, we did a lot more than we had the previous one. We started, we were still avoiding the big indoor things. But yeah, definitely, because you know they don't, I know people are giving them a bad press, and some people can't quite grasp the fact that just because it doesn't stop you catching it, that's not the whole point, you know there are some people that don’t seem to be able to make that leap that the important thing is you don’t die or become seriously ill, you know, and they're really really good at that and so, because I knew that it did, it did help me feel slightly safer, I think. And you know, I do things since I got vaccinated i'll go into shops a little bit more and that kind of stuff. I don't worry about shops as much now, I still wear my mask. Other people don’t wear theirs I’m still wearing mine I still avoid people. I'm like don't get near me thank you very much, and I pick really quiet times. I, you know I’ll nip over after the school run before anyone else gets in there. Erm I wouldn’t go over on a Saturday lunchtime, for instance (Sophie)  Oh, it's been a long time ago now did, did I meet more people? Did I go outdoors more? maybe I did in terms of going to restaurants and things like that, you did feel well, a bit more protected erm for a while, erm. I felt more relaxed about having my daughter in nursery … So erm, yeah, but I think that it was a feeling of relaxation a bit in terms of I had like some sort of defence system against it. (Stephen)  There's this weird period for asthmatics and as soon, probably the most stressful bit about it, you know the, the vaccine’s here. And then it's all to do with who gets it and in what order ... And so there's this weird thing of like, I was classed as vulnerable. So then I'd like isolated, been cautious, at work I’ve been cautious. And then there was like is it the, ah I can’t remember was it the JVCI. basically, basically it sounds bad but like basically they said if you got asthma, don't sweat it... Unfortunately, like, for asthmatics, we'd sort of been promised we're in it [priority group], and then just got left out  **Anger/frustration**  And I remember one of my friends, getting it before me. Erm and it was because they work in a nursing home, so they were in like priority group two, and I was in group three, but I just remember it really really annoying me because they'd been out like partying, and like to restaurants, and they're physically fit and well. It really annoyed me. Erm although I know, I know, actually from a medical scientific standpoint that, yes okay they did need it before me, they were more likely to get and it was more about protecting the nursing home residents than them themselves, erm but it still annoyed me, because I'd not been out at all. And, you know, I was, you know, I got my vaccine, about a week after that, so it wasn't it wasn't long after, but I was a bit like, that's really annoying (Matt)  Cos you do just feel forgotten about, completely forgotten about. And, erm with the current government messaging, you feel very expendable. And it's upsetting because it's like, you know, I’m spending my life training to be a doctor as well, like you know, and that shouldn't matter whether you know my life is worthwhile or not. But I think objectively you know I've got friends who are training to be engineers, they’re doing PhDs, and they just paint you as just kind of leeches on society, and it annoys me. It really annoys me. (Matt)  Yeah, I knew I was going to be getting it quite early on. Obviously I knew the healthcare staff were all going to be first, and but because then I was shielding I was put in with 70 year olds in Scotland, erm but I was very aware at that point that cos my husband has asthma, that he was a initially in group six I think I was group four. And then they suddenly took all asthmatics out of it, as you’re probably aware of. I, you know, we could still get the flu the flu one, but everything else was gone out of sight, another nutty decision they’ve made, but of course at that point I was quite aware of the fact that he wasn't gonna get his for a long, long time. So whereas my step-dad's 75+, he got his first, my mum and I had it about, two weeks later, he wasn't getting his for months and months and months so we were quite aware that we had some protection. (Annie)  Pfizer have never changed the leaflets, it still says, three weeks apart. And then they pushed our so we were 12 weeks. So we had to wait 12 weeks now I'm going aye okay, they've done studies on Astra for 12 weeks, they’ve done no studies on Pfizer, they’re just hoping for the best here. So that was a bit of a worry because you didn't know if they were making the right decision because countries around the world were following our stupid strategy. And then of course by time I got mine 12 weeks apart, then it became eight weeks. You know, and then similar when they decided when we were getting the booster erm I went on the day, erm I knew exactly when my six months was, and I booked it, I thought I’m not waiting for this. So I went and booked it for the very day my 6 months was up for myself and my mum to make sure it was done, and of course at that point they’re going well, to my husband well you’re six months you're not getting yours till Christmas Eve, then they changed it to three months. It definitely it's very difficult to know where their strategy comes from cos it’s very. when they keep changing it. logic would say I could do with another booster in the next two weeks. (Annie)  I didn't feel there was a community spirit, there was the, and then obviously, we were told we weren’t getting our vaccine. (Shauna)  Yeah, I mean, when it was when it was first talked about. I assumed because I get the flu vaccine every year that I would be in the priority, not the top, top priority because obviously there's people whose asthma was a lot worse than mine so you know obviously they would be higher up the list but i still expected to be on the list. Erm and then when I found out I was that that was that really really affected me. And we, when I say we, I mean the others have been campaigning, with over the vaccines. We've tried and tried and tried to get the media to take up what's happened. How asthmatic were excluded, so many were excluded. And they just haven't wanted to know, you know the media, just done nothing about it and then when they do report on erm who's getting it. Erm it was always the vulnerable people. We were affected, so therefore, were we not vulnerable? you know, its just like a kick in the teeth every time they said all vulnerable people would be called for the vaccine first, when actually, we weren’t (Olivia)  Erm and, you know, I tried calling people I tried lots of different routes saying my six months are running out... So I was just really, really frustrated by that. Erm. And, yeah, I didn't get my, I did get my booster, in the middle of December, but it was only because at that point they had opened it up to, you know, everyone over 30 so it was just because they they'd widened the age bracket. Erm and I spent, I think, two hours on the system online system just logging back in and out, just to see, you know, just trying to snatch up a cancellation. (Francesca)  [after deprioritisation] Abandoned, I guess, and anxious again. I've never been anxious in my life. I'm not someone who, I would say I'm really resilient. erm, and can see the positive in lots of things but it was really hard at that time, I remember going for a walk with my friends, who has type two diabetes. It’s just one of those things when you get to my age, and she was like going on saying she’s so relieved to get the vaccine, and the lack of empathy from my best friend was unbelievable, because she was sorted. And there's been a lot of friends have been, not quite got it either. You know they’d be like you’ll be prioritized, you get the flu vaccine, and you’re like, deprioritization you know that whole, well are we going to get knocked off the flu jab list now. So, just felt abandoned and not taken seriously for a respiratory condition. (Hadley)  as I remember though, I don’t know if you remember, one of the things, the guidance was strange when it came out for asthmatics, there was, erm you know we were told as asthmatics that we were high risk. And then I don't know at what point it was but we kind of were taken off the high risk list. I don't know if you remember that. And then all of a sudden they were saying well you’re not high risk now. Well how can we, one minute be high risk and then the next minute we’re not at high risk for a respiratory illness, you know (Chris)  I asked the nurse doing my smear, am I in group 6, and I remember feeling very relieved when she looked at my record and said yes you are. And then it all got changed, at the last minute. And it was like, I’d actually felt quite confident that this is going to be alright, you’re gonna get the vaccine and then it will be, well it won’t be normal but it’ll be much much better. and then it all changed. Oh you’re not vulnerable, oh asthmatics aren’t vulnerable unless it’s severe. And the way that changed. Was terrible (Joanna)  Which I knew that was on the booster which strictly I don’t meet the criteria, but I had, I had met the criteria for group 6 before, but only just. then they rewrote it. And yet, I thought I should have in group 6 as I was originally. And it’s like if people genuinely don’t want the vaccine that’s one thing but it’s the people who want it and cant get it who are worried. I'm worried that I won't be able to get it, so far I’ve been lucky but that wont necessarily be the case. And then I'm being made to feel because of that, that it’s all in my mind. Or is it, are we going back to the situation we had 20 years ago, when people who had asthma, some people thought it was all in the mind, and you couldn't request a no smoking area at university because it would offend people because smoking’s a social activity, and some people think it’s all in the mind. And there’s a lot of things like that, there’s things like well stay at home if you’re afraid or if you’re vulnerable. Well, I can’t. my son has to go to school. I need to go to the shop. There are things that need to be done. Wear a mask if you’re afraid, Well, it helps me a bit to wear my FFP2 mask, but the best thing for masks is if everyone wears them or if most people wear them, so being told wear a mask if you’re afraid that doesn’t, deal with the community side of it (Joanna)  But, luckily, I just hassled my, my GP and I also looked up the details and they've been this ridiculous criteria for what might still keep you in groups six and I said I am in group six according to their completely fictitious definition. So I did get my, my first vaccination with group six. I didn't group nine. But I was so angry and it's just continued to happen to asthmatics with the booster. Why not use the flu list, when they acknowledge that we're vulnerable to respiratory viruses because we are prioritized for the flu vaccination. And it just drives me mad and again I did get, because I'd had my vaccination in group 6 I got my booster. And actually I had my booster and I thought well this is looking, it looks so good for protection. When I'm about two weeks past the booster I’ll start doing a bit more again. And it was 10 days after my booster that the news of the omicron hit (Julia)  And then when I saw that most asthmatics are being booted out of group six, I was incensed. So even though, as I say, I was still within group six. When I phoned my GP about this they said oh no you're in group nine because of your age, I said read the definition, they said oh we don't have time to. So I was just horrified. And the other thing that was the media particularly mainstream media have been so bad about this, all people with underlying health conditions are being prioritized - Well no, we're not asthmatics, vast numbers og asthmatics were moved out of the priority group, and this was not being reported. made me so angry. Even though I got my vaccination in reasonably good time. It's one because I did fit those ridiculous criteria, but also because I make quite a bit of fuss about it. (Julia)  And it just incensed me that so many asthmatics were being left so vulnerable, sort of, you know (Julia)  Um, I looked on the NHS web pages, I was reading the newspaper, pretty carefully I was also erm reading explainer articles and things like the conversation. Erm, but as soon as it was available I was really anxious to get it. I'm a huge believer. Well, as someone who gets the flu vaccine every year as well. You know I just really wanted it, and then as soon as the flu shot became available in autumn of, 2021 I also, you know, erm my GP said we have them, you know, come down at this time and I got there, there was a long line of other worried people (Susan)  and I think at that point I just lost all faith in the government to be honest in how they handle this I felt completely let down, and it felt really, really, unfair, because you know you’re told for a year, you're going to die if you catch this and then you're like, oh no actually you’ll be fine, actually you’re less likely to die than someone who's healthy and I think all the way through, it was, the unfairness was that I didn't want to get it before people who were more vulnerable than me, and I’ve got friends family who are you know extremely vulnerable, and I wouldn't have wanted it before them. But I didn't think it was fair that my husband who is 3 years older than me, but completely healthy, and he didn't either, he was due to get his first. That felt so bad to me that felt so wrong. And that was the point that I, you know, and by the time I actually managed to get the vaccine it was only by chance, my doctors said No sorry you're not on the list we're not going to help you, and it was a bit of a postcode lottery, because I know other people were rushed through by their doctors whether they met the criteria or not. My doctors didn't let me. And a friend, it was a friend told me she was messaged me and goes, do you know at another doctors, they're doing a walk in and they’re taking anyone over 50 at the time in the vaccine groups or clinically vulnerable, and I was like, well, I’ll go chance it and I just went up there and I was like well, and I am asthmatic but I don't meet the criteria, and they just said, Okay, you're asthmatic, come on in, you can have it. But then I spent the next 3 months worrying whether i'd be allowed the second one. So I think it's like , I sort of snuck in and got it, but erm they actually went back 3 months later and redid every one they'd done first time. So it was fine. I got it (Sophie)  **Stress vs relief**  And this was when delta was surging, and I was like, you know I know that I've not got as robust an antibody response as other people would have done to two doses so I need this third primary dose. And it just so happened that my consultant, my asthma consultant, finally rang me, and I just said to him by the way you know can you please just give me a letter saying I need to have my third primary dose and he said oh yeah sure not a problem. And then like literally five minutes later had it in my email inbox, I printed it off and just went. And I was like, this was so much stress. for absolutely nothing. And when I went, I did it, they wrote it all down and did it on like paper and instead because the government system wasn't updated to register third primary doses. Erm and then, had it, got it. No problem (Matt)  I thought, as soon as I’d been injected I was like, thank goodness, I've made it. I've actually made it. Erm because I was just scared about, you know, when you see in the news, they’ll let the bodies pile high and things and it’s like they’re talking about me. (Matt)  I didn't feel there was a community spirit, there was the, and then obviously, we were told we weren’t getting our vaccine. that was really stressful, which did make my asthma 10 times worse, that was really stressful because, i well i thought I was alright, well I was annoyed for all asthmatics, erm but I thought I was okay, because I knew I'd been in hospital as a younger and I knew I'd had loads of pred when I was younger, so I was like well I should be alright, but nothing came and nothing came from the doctors, and then it was the weekend he said, said, oh we're going to stop, you know, they said they were going to have a break from giving first vaccines (Shauna)  I was in the priority group for the first lot because of having had hospitalization. But then they changed it for the second lot. So that was, that was quite stressful at that point. Erm, and this was before last Christmas, but because I’d been vaccinated early the first time my vaccination had been, I can't remember how long it had been then, but it it had been like 7 or 8 months and people were getting vaccinated who were slightly older than me but weren't high risk. And then, because I was under 40, I wasn't getting vaccinated for a long time, so that that was quite a worrying time, and I did cancel quite a lot, of social things then, I was basically unvaccinated because It'd been so long (Amy)  On my records, there's hardly any mention of my asthma, never mind, all the stuff I’ve had, time in hospital and what have you, none of that's in my records, so I’d been taken off the list. So my, the surgery couldn't verify it without the senior practitioner nurse to do to say I could have it and she was off that week. So I had to wait until the end of the week to find out, and I'm not joking I must have cried the whole week. Worrying about it, and I mean they were great in the end, they sorted it out and I got the vaccine as group six. And it must now be on the record because I could get my booster at the right time. Which I know a lot of people were struggling with. erm, but yeah that, and that must have happened to so many people, because my records are nowhere near up to date when I look at them that there's all sorts missing, erm not just about my asthma. so that that was very stressful and I think the whole then would I get my second dose, then would I get my booster dose, that's just added to my anxiety. I've tried not to let it, but it has. (Shauna)  erm and then on top of that all that, what happened with getting the vaccines and priority. Erm and, I mean I was one of the ones that was removed from the priority, and had to fight to get to get a vaccine, I mean going back to my surgery, they are really good and my GP put me forward. But the, the stress of it really did affect my asthma. (Olivia)  awful oh, I had a breakdown. Yeah, yeah, I think, I think the stress of the whole campaign everything that we've tried and been through and failed. And erm to be honest as asthma UK weren’t being helpful either. We wanted to work with them but they didn’t want to know. And, yeah, yeah, well I mean when, when the announcement came that we weren't included in boosters. Yeah, I had a breakdown and, yeah, just kind of went into a shell for several days and didn't want to talk to anybody or see anybody or say anything, yeah, pretty, pretty horrible (Olivia)  I didn't you know take the foot off the brake as far as you know, you know looking after ourselves, and making sure that we didn't go places we didn't really need to. But, um, I was excited because I thought well hopefully this one step to getting back to some sorts of normality, you know (Chris)  Um, well I've had my booster so that's helped. Um, but yeah I mean the stress is still there because whether you know if there's going to be future boosters for variants, or annual boosters. I feel quite stressed over whether or not I'm going to be eligible for those. So that, I think that's still having an impact definitely. (Olivia)  Overall, erm the camaraderie between all of us who were campaigning was fantastic and knowing that there were other people out there in the same situation where you had, you know, we were all supporting each other, you know, if somebody's got a vaccine we were all celebrating you know and just, just that that that sense of working together with lots of other people who actually understand what it's like to have asthma. And you know, and all the concerns. It was amazing. Yeah, just really frustrating that it didn’t get us anywhere (Olivia)  Initially, yes, I think I did there was, there was a huge sense of relief having it and thinking you know yeah now, now I'm safe or even if I get it, it won't be as bad and also knowing that everyone around me had gotten it erm you know, fairly recently and, yeah, I think I felt a lot safer but obviously that wore off as soon as there were reports of, you know, we don't actually know how long this is got, that immunity is gonna last and yeah I think I was, I was very happy for about three or four months and then the concerns started again. (Francesca)  wouldn't want to say I’m, what's the word, invincible now I've got my jabs but I've had the three. And like I say, I'd say my stress levels or anxiety, whatever you want to call it, is just like half is thinking, like I probably looking at, some point I'm probably going to get it, and it probably will not be over for a couple of days but at least I’m not gonna be looking at hospital. So there's, there's now, there's now more of, there's more of a balance is it from the physical, so like, protecting my lungs to the mental health wellbeing. Whereas before it was a bit like I'm going to stay indoors and my mental health’s rubbish. But now I'm like, well, you know what, it’s so and so’s birthday so I'm gonna go out and then I'll have a couple of days indoors and then like hopefully I've not got it and so, it sounds, like I'm now so prioritizing social stuff, but for my own like, wellbeing whereas like I say before it was, there’s danger outside the house so stay indoors. (David)  Just, just so much improved. It sounds, I think I'd say my rel- I think, as an asthmatic your relief level or if you had any form of, like a couple of people know had like recovered from cancer, just having the jab just gives you that sense of, just, I wouldn’t say joy but it's like you've been on edge for two years, or for 18 months. So to have to have that form of protection is bit like you know you're not immune or anything like that, but you’re just, you've got a bit of a fighting chance now. So I think, yeah, like immediately after that I slept better. Like my friend was saying you know you’re now more glass half full than glass empty. So just sort of like a general relaxing, slept better than I did, but still not great but yeah just more a positive outlook (David)  mentally, I felt much relieved to have had it done. I mean I was quite nervous the first time, cos it was, partly because the blood clots had just been in the news, but also I've heard horror stories of people being turned away at the vaccination centres, being told you’re not eligible yet, even though we’d been able to book the vaccine. Luckily that didn’t happen to me. the fact that when I bought my first dose I had a choice of booking it between [location] or [location], and I chose [location] because I thought that might lessen the probability of that happening. I thought it was going to probably be medical people in charge, there’d probably be people who've got the sense to know, hopefully they'll have some sense to not do something like that. Whereas I was scared, I was more scared that rugby ground vaccinators might be like that. I’d say for me I was just relieved to have it done (Joanna)  I will say something with the vaccine, is the first time I ever got my first dose. I picked my son up from school, and I was amazed at how moved he was to find out I’d had first dose of the vaccine. he said he could stop worrying about me now, and I didn't even know he’d been worrying. You know people have families people have children, they have to look after young children, it’s like stay at home, it’s just ridiculous. It’s just silly because you can’t, people have stuff they need to do or that they want to do. Well wanting is, that’s not what they actually want to do but it’s stuff that they… can’t put on hold indefinitely (Joanna)  if it [catching COVID] had been before then, then I just would have been absolutely terrified even more than I was. But because I knew that the chances were it probably wasn't going to kill me now, the, I’m so grateful for the vaccines. Lets put it that way. (Sophie)  And then suddenly and there's Boris Johnson on the telly going, oh, yes, everyone including the asthmatics will get it if you get the flu jab, and then they went, oh, no, actually, unless you're severe by this funny, made-up definition that they've plucked out of air, there you go I’m getting really political now, aren’t I, erm they were like, oh, no sorry you’re not having it and I’ve never had to have oral steroids, because It's well controlled. never had to go to the hospital again, because it’s well controlled because I take my medicines every day, but I have had to have high levels of steroids when I have a flare up. Really high, you know, inhaled but because it’s inhaled. So, though for some reason, although I have to get the free flu jab, every year, I suddenly, they went, Oh, yeah, if you get flu, you know asthma that's bad, but if you get covid, off you go you’ll be fine! And that was awful and stressful, and I felt, kind of to use one of the modern words that my teenager would use I felt gaslighted by it. because I’d been told for a year, you've got to stay home, you've got to stay safe you know even if you're not shielding, you need to be careful and then suddenly, it's like oh, actually, no, you're fine. (Sophie)  It’s, I think it has just been that the kind of lack of clear guidance has been more stressful. Not knowing if you’re gonna get the vaccine or not, not not being prioritized for a vaccine when you were the first time, things like that have kind of added to the stress more than anything else I think. (Amy)  **Loneliness vs companionship**  [after vaccine] kind of like okay I feel like I can, you know, I think I started going back into the office sort of thing. So from a mental health perspective work, work were noticing like, you know, people were you know struggling, sort of. We have like a big, we have like 150 people in an office quite a big loud boisterous place to be. So you can go from that to just sort of being on your own, had affected quite a few people so yeah I think, really positive to get the vaccine just more yeah less, you know better sleep better outlook. (David)  ***Long Term***  **Danger/fear to safety/calm**  But even now, you know, we're, we're supposed to be going out, you know, me and my partner, are meant to be going out for a meal for the first time in two years tomorrow night, together. Erm, and we're sitting inside. We've been sitting outside and for the first time we’re sitting inside. And we're really nervous about it, you know, because we know for so long now we've been on, you know, Red Alert as far as we’re concerned. And I'm thinking, Well, why are we doing this, why, you know. But, it's a case of, you know, we haven't got the government to kind of protect us, so we know what to do to protect ourselves, but we can't stop living, that's the thing, you know, you we wasted two years, two years have gone now, you know, and we can't really waste any more time we just have to try and step out there, a little bit. So, yeah, final thoughts I suppose (Chris)  Yeah, it's, It's made me realize I am in a vulnerable category and that that's something you know that I have no no idea what's really going to happen if I get it again or if there's a different variant. But I now have started to become quite frightened of it. And I keep sort of thinking I won’t bother going into the doctors next week because my asthma’s going away and then I'm like no, I've got to protect myself properly now, otherwise yeah another variant could come along that could completely wipe me out (Molly)  [when contracted COVID] Yeah, it's just that feeling of like abandonment really I think a lot of people are feeling the same way, just think like, okay, a lot of stuff that was never on repeat prescription for me they just put on repeat prescription during this whole pandemic and some of it’s supposed to be like asthma is supposed to be regulated, and it just felt like they, you know, didn't care anymore, what was going on. And you should never, in the past I’d never been allowed an inhaler without a regular kind of meeting with the nurse, and I just couldn't believe that they were just writing prescriptions for it. And I know that they were overwhelmed, but usually if you've got an extra prescription for inhalers like that it would trigger, like a meeting to say that you're not managing it very well and that just didn't happen (Molly)  your world becomes so much smaller. You know you're not sort of taking journeys. So, a few months ago, that was my first trip, since the start of lockdown. To Bristol, and I remember feeling so anxious getting on the train. You know, and I made sure I booked and then the three people in the section was sitting in. No one was wearing a mask. You know, and it's like, oh my god. and then the train coming back that was exactly the same. You know, and that feels really scary and you still have, I think it's stupid Boris, you know saying that people need to wear a mask, but then seeing that he doesn't, you know. But yeah, it does feel… (Daisy)  And then the Christmas 2020. Although it was annoying being in a lock- being in a tier 4 during Christmas, I was like, I didn't mind, it didn't bother me that much. In a way, it felt safer being locked down, although it was particularly annoying that it all came so suddenly, if people had been able to prepare beforehand for the fact that it might be happening, people wouldn't have been rushing around like headless chickens getting supplies, and not really worrying about transmission. For me it was mostly okay other than I had to organize postings and gifts for people I couldn't meet up with, because I only wanted to meet up outside anyway (Joanna)  I mean, I was to some extent that like up until end of November like, I was… not as much but like, I’d not really gone shopping in London. I've been into business meetings in London, I went to a function which I didn't realize how many people were there and I was really uncomfortable. So, but I guess it's that apprehension and anxiety again, still in the back of my mind and I want that gone (Hadley)  **Anger/frustration**  I was doing a therapy each week remotely via telephone appointment. And I've continued to do that throughout the, throughout the pandemic, which is, which which has been helpful just to give that release and then actually day to day. Although I say that, you know, I was angry about the politicians I'm certainly angry at the moment, erm I can, like I say compartmentalize and just deal with it at one point, and then not let it affect the rest of my day (Matt)  I mean I knew, without getting too political, I knew that this was about worst Prime Minister and worst government in history, I'm a historian trust me. And I didn't care, but I have been repeatedly horrified terrified, and incandescent with rage, about the lack of care for the general population, and particularly the lack of care for public health. And erm the tribal apportioning of blame the way we've wasted so many billions of pounds, and how much better it could all have been if we’d actually had a decent government and leadership, acting responsibly, and the money that's been put into people's pockets had actually gone into the NHS and public health. And if we done a New Zealand. you know, and I try not to let myself get too wound up by it. I'm only on twitter and I was only going that last February because I was so angry about the asthmatic being kicked off the priority list. And I try and limit my Twitter activity to short, a couple of short sessions a day. I try to not let myself get wound up, but I just can't help it, you know, and I feel asthmatics in particular have constantly been thrown under the bus, either ignored or deliberately put at avoidable risk. And it's something I'm not gonna forget and not going to forgive, erm if any good comes out of it. Well first of all, I found the asthmatics at risk group. I've actually virtually met some really nice people. And sharing experiences and sense of solidarity so if any good comes out of any of this, it will be things like your study. And asthmatics generally because we know what it's like to have a chronic respiratory disease we are going to behave more sensibly than most people in a pandemic of a respiratory disease. And it hopefully, some of the drugs that are being developed might also help with asthma. But I really desperately want this turned into something that will help asthmatics and help make people take asthma a little bit more seriously. Maybe a campaign that says you know you or your friend or your relative for somebody who had from was telling you how terrifying it was to be very short of breath, not be able to breathe with covid well that's the experience of every asthmatic. take it more seriously. So I try not to let myself get too wound up, where there's very little I can do about other than signing er, signing petitions and emailing whenever possible, and I do donate to some of their campaigns, keeping myself as safe as possible so I'm not a burden on the health service. But I also try and try and keep that positivity that may be something good will come out of this (Julia) |
| Being left behind |  | **Danger/fear vs safety/calm**  I just felt more free to just go outside, even on my own erm for a long walk and then maybe even quickly going to a coffee shop, get a takeaway coffee and just just do something like that for myself, which is erm, which I really missed. So, so I think there was a little sense of liberation, but I was still very very cautious and I don't want to say anxious that's the wrong, that’s too strong, but I was very cautious about the outside world. (Francesca)  It seems crazy to say it but but yeah I'm really, I think with my close friends, I just wasn't that frightened about having them round in the garden or like going out with them and doing stuff that just it didn't, didn't scare me and I have absolutely no idea why it's probably just part of my nature, but I think I was just pissed off with it all by then and then just thought, yeah, like I've got I've gotten this far without getting it and, you know, incrementally you make little risks, and then then if you don't get it, you start to think, well, I'm, why haven’t I got it, am I just one of those people that isn't going to get it and then I just got too cocky about it and thought, you know, me and all my friends were being really careful so what's the likelihood of me getting it but, yeah. (Molly)  as time progresses, it progressed, it was obvious that young people were reasonably safe compared to old people and since I'm more on the younger side, I thought that would probably compensate and because my asthma isn't very, or is very well controlled and I didn’t really have any issues with it, I started to feel quite safe. (Francesca)  That was as hard as going into lockdown, I think, coming back out of it, because once I got used to the lockdown I felt quite safe at home. .. Obviously, it was, you know you could stay safe. You could make your own decisions about who you saw and what you did. the big thing for me was the kids went back to school at the end of that first lockdown for 3 weeks… and I kept them off. Erm because I was just terrified with them going back to school at that point. I was like well it's holidays summer holidays in 3 days, so you can stay home. That was one of the tough points, because some of their friends were going back, and I was, I was just constantly having the conversation with other parents going i'm keeping off because i'm vulnerable. (Sophie)  I was just more grateful to be able to socialize. So I wouldn't say I was like overly worried about it. But I was more like okay I need to keep taking my inhalers and I booked an asthma review as well like when my chest was wheezing which I don’t think I would have ever done really. Erm, yeah, but just things like that but I wasn't like overly concerned but probably more cautious, than I would be.. I think because I was being told it’s severe and that you’re vulnerable and things (Louise)  I wouldn't say, like, it was too much, of a mental strain really. It was more just me being like overly cautious. maybe not even overly cautious just like thinking about it like the effect that it has on my asthma, and not even just covid but like anything really (Louise)  like most people we were ready to get out and see folk, but still for me it was very much a concern that not picking up covid was the primary determinant in what we did, so we very much kept to the rules. erm Didn't go into people's houses met folk in the gardens erm. I think that was the time of eat out to help out as well. And there was again, that was like, Well, we need somewhere outside, so we were still very, very careful. (Stephen)  more nervous, partly because they've always been presented as restrictions rather than safeguards. Erm.. mask wearing was never that great on public transport. For example, about here, even during the height of it. Erm and most I would say a lot of people, would take up an opportunity to abandon it. Erm. Which is concerning, it was very concerning, but certainly as the advice then was still very, it modelled it was still, there was still too much emphasis on hand washing and that sort of business as opposed to wearing a mask and keeping your desk and keeping places ventilated (Stephen)  the director at time was very much keen for everybody to be getting back in as soon as possible, because she was a control freak. So but. Well, if I’d had to go back into the office, I was very much scared of having to go back into the office then (Stephen)  erm, yeah, I was ready for it, like the social side of it. But then I guess there was like a bit of anxiety. I guess my anxiety was more like, how would my immune system respond like not just in terms of covid like spending so long inside, and then I just thought everything was going to make me ill and make my asthma flare up again. (Louise)  But I used to work out at the gym fairly regularly, and I don't anymore, not just because well lockdown was part of that in the thick of it, but well it's an environment where people are sweating and breathing out hard, and it's not always that well ventilated. So my physical well-being has gone down in the regard. also, if I'm at home I comfort eat, I stress eat I will be fat. I'm moving house soon and I've had to chuck out quite a lot of shirts that just don't do up around my tummy anymore. Which is sad erm physically and mentally and financially (Stephen)    Yeah, it was strange my husband made me go out to which is a good thing. erm because I'd become very insular erm and I think that's the resounding thing from all of this is I used to be the life and soul of the party. And now you have to push me out the door. Erm and I think that started then, that I could, especially before they started putting masks in place. I was literally, I literally was shaking going into a shop. I was, I would dip in and out and even, this is even now, I only go in the shop, if I need something. My husband will pop in otherwise. And so, yeah so I was, I wasn't in a good place and at all. That was harder than the lockdown. And that, cos anxiety and that and that does affect my asthma, I was I was up and down like a yo yo with my asthma, erm I didn't raise any medication or anything because I was too scared to contact the doctors. Erm, yeah, it was… that that was a really hard time (Shauna)  I do go out more now, and do things but at that point, I literally was dragged, literally dragged into the shop. dragged to do anything, we went to, and I wouldn't go near my mum and dad we, we've all been really strict in our family. We've, like if they came around, and we were planning for a day when it was sunny outside they only came in the house to use the toilet and I had everything cleaned. That's one thing that has, we were thinking, my asthma nurse, since covid started I clean everything, top to bottom all the time, and we think that's what's affecting, because I'm now using lots more chemicals. (Shauna)  And when I did go back out again because my husband was like, this is not good for your mental health you need to go out. erm it was like, I was a scared rabbit, I'm still a scared rabbit in headlights. I literally shook erm, but when the mask wearing wasn't in and people could go in shops and stuff, I wouldn't go in. It's only since that mask wearing and I've kept going, obviously, the fact that everybody was locked down. And we were kind of all in it together got me through that one (Shuana)  So, you know, so, a friend of mine would maybe you know, come down and sort of yeah we'd say go for a walk and keep a metre distance and things and just, yeah, I'd say, super cautious until I got those two jabs in my arm which sounds bad but it just, it was sort of like, I think it was, if it was anything else I'd be like, I can be like I could bat it off but it's a bit like, at the time, it was said asthmatic need to be cautious and yeah you just have that drummed into you. Yeah, so it was kind of like well, and I'll be I'll be honest I'm quite a, I'd say a cautious sensible person. I try and limit what I drink, I wouldn’t say I never let my hair down I'm quite a, I just like to be in control. (David)  I was pretty worried. And there were actually two swimming lessons, either side of November lockdown where I actually refused to take him. I said, I emailed the swimming people and I don't feel safe to do this, and I’m not going, I don’t feel safe. (Joanna)  Um, I was fine with it erm I remember... Yeah, I was fine with it. I did think that some people weren't really, you know, people were so busy trying to get out and be free, with their lives, which is understandable that, you know, I think they were forgetting a lot of the people who were clinically vulnerable because I had a close friend who was going through chemo who was absolutely terrified the whole time. Because once restrictions were lifted. She couldn't really count on people she encountered, having, not, gotten it from someone else. (Susan)  But it was like I do, I want to still do that [go to charity shop], and I felt very much that I couldn't give up everything. And it was driving me mad not being able to do that. And I felt I had to do that And, but it was still very limited and I felt scared because, we weren’t back to normal. It was scary. But I felt I had to, I was sort of being very very limited in what I did. I think there were a few things that I did have to interact with because I was trying to deal with workman cos they were renovating a house, and I had to deal with them, and that was. There were occasions when I just had to do it and I’d know that they weren't in masks. But yeah, sometimes they were sometimes they weren’t. which happened during that, I think there was a bit of that going on during that time (Joanna)  you can’ t stop everything but it was, it was difficult and it was sort of just, I felt it’s nowhere near normal, and it's not going to be. I don't know how we’re going to do the new normal (Joanna)  erm I ambivalent, because looking back actually I was probably safer than I thought, but erm I was still wary, I thought not only did we lockdown far too late, and not holding off, but we released as, as has become the pattern we opened up too quickly and too much. I thought I’m not desperately happy about this. I did see more people but in the very careful situations, and more generally, I thought, well, if I can avoid going to anywhere that's crowded or inside or I don't have to that I’m going to (Julia)  Um, fine except that it's been a little strange in terms of, you know, I just feel like I’ve forgotten how to get along with a lot of people at once, and it suddenly made me really conscious of how much I'd taken crowds for granted and large groups of people. And that I was a little wary of being in a large crowd at that moment. (Susan)  **Anger/frustration**  I had to force myself literally out the door. erm, and at the beginning people weren't wearing masks and things, erm in shops. So I found that very stressful, and I just kept, and I still say it to myself now, I kept saying if you knew what it was like to live in a hospital bed and not breathe, you would have a mask on. erm, and I still say that. I know there's lots of people who can't wear masks for medical reasons and that's fine, but it really winds me up when there's people just take the mickey. Because if you do, you would wear a mask unless you absolutely couldn't. So, yeah, so that was really worrying. erm, and I still think that we didn't know really about what was happening with vaccines and treatment. (Shauna)  it's gonna happen you know enjoy seeing other people but please be safe, because you do not want that, and it's just really annoying because my predictions ended up being true. And then everyone was like oh yeah you were right. And a lot of these people I was speaking to were medical students. And I was like i know that it's, it's, some of the mental gymnastics that people do, to say that, oh it's completely fine for us to go to a nightclub or, I mean, nightclubs weren’t open at that point but, it's completely fine for me to go out and have like a house party etc. erm because you know it's more important for mental health. That used to really annoy me. Really really annoy me, because I've been, I've been diagnosed with complex PTSD, you know, I've got a severe mental health issue which I manage. And these people who've never given a toss about mental health before, have always been quite offensive and quite invalidating, are now saying, oh well mental health is so much more important than stopping the spread of an infectious and fatal illness. So, I was angry, quite a lot at that point, and I just thought, you know with these people, I'll speak to you, and we can have, you know, great chat and you know banter and things, but I'm not speaking to you about covid. Like that’s it. you know my opinions on covid. So. that's kind of what happened with that (Matt)  **Loneliness vs companionship**  erm again, I just kept myself, kind of limited and didn't really go out with like my social group, unless it was outside. erm it took a good few months for me to be able to ease my way in (Robin)  erm. I think just really limited our interactions with other people, any kind of mother and baby things that we went to, they were all the ones in parks and outdoors that. We kind of really just deselected anything that was indoors even if it was a rule of six in inverted commas. So. So yeah, there was. There was still that restriction. And I know that I found it hard my wife found it really hard just that sense of isolation and also that feeling that you were, that I was causing other people's lives to be more difficult because we had to do things to take into account my asthma (Stephen)  erm. It was tricky because we met up with some of my wife's friends who've also had children at the same time as us and it was, they were all kind of ignoring the 2 meters, hugging about, swapping babies around. It was hard because you start to judge your friends, and not in a good way. Erm and people judge you they think, Why are you bothering with 2 meter crap? Why are you wearing a mask outdoors if you’re meeting a group of people? so it was all a bit weird. (Stephen)  Erm to be honest I’ll look forward to, I've just been meeting up with people that some of them are my mates from the football team, that were meeting lasses off of tinder and things like that and it’s you know it's like erm well it's kind of your decision, but one the longer it’s, like am I at am I at risk for having met you, have you put me at risk because of your behaviour .. : it's lockdown gonna go on longer because of your behaviour and I I that found that really affected how I viewed people (Stephen)  That was, that was really that was a real low point I think of the whole thing, that's probably worst I felt because you know I felt bad that I wasn't letting her go and see her friends [by keeping her off school], and they were both getting a bit lonely and my youngest all their friends went back. So I was keeping them at home. We couldn't do all the face time to friends that we'd done before, that kind of stuff. So that was that it was the worst bit (Sophie)  the main thing was stressful and lonely knowing that everyone else is, can just, like everyone just took their masks off at midnight didn't they so, it was like do I keep mine on do I keep myself safe? Erm yeah, and especially like, my partner works in hospitality as well as it was quite, like for me it was quite like I don't, I don't know like daunting, like you know, coming in contact with loads of people and then coming home. Yeah. (Robin)  nervous, very nervous, because at some point, ultimately, we can't afford to stay in a lockdown for three years, and get the cases down to zero, because errm the NHS has still got to carry on, the police, we need food we need heat and light, so it's a bit it's sort of a bit like, “well we're going have to, as long as 80% of the people are okay the other 20 well, you just have to look out for yourself a bit.” And I always felt like in the in the weaker 20 or 10 thinking, yeah it’s alright for my two mates because like say they're at the gym every day and fit and healthy, but I need to be just cautious. So yeah, I turned down a lot of social things even though you were allowed (David)  I said to my mate, and he was like “ah you’re allowed you’re allowed”. I’m like “yeah, I don't care. Yeah if you want to meet up and chat we’ll go for a walk and go grab a coffee. If not, it’s alright” and so we were doing obviously a lot of the, I think, we were using zoom back then we were doing like zoom quizzes and Pictionary and just a lot of online gaming and stuff so like your play stations and websites that you could do quizzes on and escape rooms and stuff so like tryna be sociable and like Facebook Live video calls and stuff. (David)  I think it was hard I didn't, I, I didn't really like any of that. I found the whole thing, just divisive and sort of socially hard and it made me feel very isolated and erm I dunno, a lot of my friendships became difficult. Yeah, I think it was mainly down to my sort of view of it all, but it felt horrible at the time. Erm wasn't a really, you know, made me feel very uncomfortable and paranoid about people and relationships and things and see I didn't enjoy that period of time but it doesn't, it didn't sort of, I think I got through it, like, like everybody else did. (Molly)  I think it's just a case of sort of, you know, not seeing people as much as you normally would do, the rule of six made it difficult to be involved with all the different kind of crowds that you normally interact with, and you might not be involved in one crowd, it was like being in the playground again. Erm I have quite a lot of different groups of friends, and erm it just became. I didn't like it, either way, whether it was me that was being excluded or whether we had to exclude somebody else or, I hated all of it like that kind of thing I just found that really difficult. And, yeah, I think that was that was the reasons why I wasn't happy with, around that time. (Molly)  but I think a lot of the social connections had been, had changed quite significantly, as a result of all of those things. So it was, it was a long-lasting effect and it kind of made me much more cautious about organizing things which sounds ridiculous but I don't know like it just changed the way I viewed, my friendship groups, which was quite difficult. (Molly)  But my social life really took a dip at that point. Reason being, everybody else was going out and had the rules of six and you know, they weren’t in a lockdown anymore. So it was very, I thought, you know we’ll forget about him now, so that, yeah, that's kind of what happened around that time (Matt)  it was a different situation from freedom day of 2021, because at that point I'd had both of my vaccine doses. erm so at that point I was feeling okay to meet up with a very limited number of people like in a park. so, like we had drinks after, after our exams are finished, erm that was quite nice. (Matt) | **Danger/fear vs safety/calm**  I felt quite vulnerable and exposed outside and I didn't understand like when I saw pictures of people mingling if it was in a park or outside a cafe, it just, it just seemed wrong to me (Francesca)  **Anger/frustration**  because I did it in my own time I felt like, I was more comfortable but if I went into a restaurant and seeing somebody without a mask on it, erm it, I’d find it quite frustrating, erm but I wouldn't have, if the bar was too busy or anything I'll just go somewhere a lot quieter. Erm, being around other people was not as scary as it was in the beginning but, it was just still a concern that, I didn’t wanna be in a crowded space. just kind of being a bit more like aware of where you’re going and that kind of thing. (Robin)  I remember it was Christmas we had the most unholy row, because my oldest daughter wanted to go out at Christmas. And we would, and it was the most. It was so, it's like not only - and I don't think I ever think I thought about oh you could pass something on to me. It wasn't like it was like you could get the virus (Daisy)  I was trying to protect myself but I was also trying to protect my kids, you know we home-school basically so we were able to keep our kids away from things a lot, erm the we did it ourselves you know, so they just zoom called their friends who are also home-schooled you know so they kind of used to that type of thing. And that's what I was doing, I was protecting my kids as well I was thinking yes, okay, if I get it. It could be very very bad. But if kids get it could be very very bad too, you know. Erm, so, my friends didn't understand that to a certain degree because, you know, two of them specifically don't have kids, you know. Erm. So, life just carried on as normal for them (Chris)  **Loneliness vs companionship**  I felt positive because I could do more, and I could see more people, like we could go back to, because I was allowed to go back to volunteering, which was very nice, like we had to wear masks there as well. But it was more manageable and, yeah, it's, but it just worried me about being around people, and like being around so many people and just like the worry of catching it because I went to a concert for the first time in like two years and it was quite frightening to go, it like going in and being around, because there was no like gaps between the seats or anything we were just sat with people … But it was worrying because by the end of it, everyone got so close, and you get like, keep away! (Millie)  Erm, again, it wasn't like I started socializing a lot because actually the, the few friends that we had down here, moved away during the pandemic so by the time we came out of all of the lockdowns there wasn't really much socializing left that was, like that could be done locally so it was either a big undertaking like a day trip going to see someone, or just me and my partner. So, it was, life didn't change too much erm you know I think I just tried to live a normal, more or less normal life, still working from home, because it’s convenient (Francesca)  Yeah. Very much so again it’s like this leaves me so much more vulnerable than other people… er it was good to see people, I would I would be worried. Erm. I still am when I meet people if im honest but the need, need for companionship and the need for my wife and I to see someone that wasn't just each other in person! (Stephen)  as soon as we, the, asthma was lifted from being sort of like really high risk. Erm once you start learning if you’ve got it, it's okay, then you start socializing a little bit more and, yeah, it's kind of like disappeared, the kind of stress of people with asthma, even though it was lung related, but a lot more easier to socialize, erm after a few weeks but at the start it was still limiting contact and doing things outside. (Robin)  In fact, we still haven't done a restaurant or a cinema, or theatre, or anything like that. Even now (Sophie)  yeah. Well. It was good. it was good for us to be able to do stuff, for example, like we were finally able to take my daughter to go and see my parents, to go and have a small holiday, erm and to be able to get away from just our local area was great because we felt very closed in. (Stephen)  Yeah, erm it was nice, after so long, to be able to see people again. And I think when you start to see people again, you sort like, like I say at the time, I thought I was coping with it quite well. But then, when you do see people again, you think oh, actually, I have really missed this. I have missed being able to see people. so yeah it was nice when you were able to do that, despite kind of the stresses that go with it, erm yeah (Amy)  my therapist had said to me, you need to do it little by little because you know you might get really overwhelmed and things. No, it just felt like going straight back to normal. It was fine you know i think that's, that's because the friends that I was trying to meet up with were very understanding of where I’d been, and so there was no comment about, you know, when we'd like, that I’d have my own bottle of alcohol, and they'd have theirs, so you know, we weren’t sharing and things, or that I was using quite a lot of hand sanitizer. erm there was no comments about that, you know that that was just seen as completely normal. erm, and so it felt like you know just going, going back to pre pandemic life, to be honest. (Matt)  I didn't do anything enormous like go to the Christmas markets or go to a big outdoor concert or a concert for that matter. Erm, I was still relatively cautious about, you know, erm, I'm happy to go to restaurants I'm happy to go see friends and erm sort of ordinary socializing. Erm but I'm not ready to be, I did travel to London and I wasn't thrilled with being on the train, or in a crowded train station. I was still more cautious about it. But I think, I don't know how much of that is worrying about my health and about asthma and how much of it is just that it felt weird after 18 months, to suddenly be in a big crowd again. So yeah (Susan) | **Vaccine**  **Loneliness vs companionship**  I felt much better. I felt like I could be safe seeing my friends who were more vulnerable, which was pretty important at that point. Erm, And it, erm yeah. It made a huge difference for feeling safer because, also because I'd had covid so I was like by that point, okay, I'm not going to get it again anytime soon. I felt like I could go out more, I think, also, I felt like again I could go see friends who were more vulnerable. Yeah, and my friend who was going through chemo was very vulnerable at that point, and it was a relief to be able to go see her and help her. So, I felt safer from other people but I also was less worried about catching it and taking it, and giving it to someone else. (Susan) |

COREQ Checklist
